# Metabolic pathways of the wheat (*Triticum aestivum*) endosperm amyloplast revealed by proteomics

**DOI:** 10.1186/1471-2229-8-39

**Published:** 2008-04-17

**Authors:** Frances M Dupont

**Affiliations:** 1 Western Regional Research Center, United States Department of Agriculture, Agricultural Research Service, 800 Buchanan Street, Albany, CA 94710K, USA

## Abstract

**Background:**

By definition, amyloplasts are plastids specialized for starch production. However, a proteomic study of amyloplasts isolated from wheat (*Triticum aestivum *Butte 86) endosperm at 10 days after anthesis (DPA) detected enzymes from many other metabolic and biosynthetic pathways. To better understand the role of amyloplasts in food production, the data from that study were evaluated in detail and an amyloplast metabolic map was outlined.

**Results:**

Analysis of 288 proteins detected in an amyloplast preparation predicted that 178 were amyloplast proteins. Criteria included homology with known plastid proteins, prediction of a plastid transit peptide for the wheat gene product or a close homolog, known plastid location of the pathway, and predicted plastid location for other members of the same pathway. Of these, 135 enzymes were arranged into 18 pathways for carbohydrate, lipid, amino acid, nucleic acid and other biosynthetic processes that are critical for grain-fill. Functions of the other proteins are also discussed.

**Conclusion:**

The pathways outlined in this paper suggest that amyloplasts play a central role in endosperm metabolism. The interacting effects of genetics and environment on starch and protein production may be mediated in part by regulatory mechanisms within this organelle.

## Background

Wheat grains are a major source of human food. Desirable grain-fill traits for bread wheat include high rates of starch production for yield, and high protein content for bread-making. However, there is an undesirable trade-off between yield and protein content that is determined by genetics and environment [1-5]. A better understanding of the role of the amyloplast in endosperm metabolism may help to understand and ameliorate this trade-off [6].

Some aspects of the regulation of starch and protein accumulation in cereal grains are understood. Prior to the grain-fill stage, yield is increased by increasing the number of tillers, heads and kernels through genetics, fertilization and water supply. During grain-fill, yield is determined largely by starch accumulation. Starch accumulation appears to be mainly sink-limited, whereas protein accumulation is source-limited [7-9]. Manipulation of the sucrose supply to the grain does not have a big effect on starch accumulation, and CO_2 _fixation apparently is more than adequate to maintain good rates of grain-fill. In contrast, the nitrogen supply greatly affects protein accumulation and kernels are flexible in the amount of nitrogen and the amount and type of protein that is accumulated [9-11]. Breeding for increased grain yield tends to increase starch more than protein, resulting in flour with a lower protein percentage, and there seems to be a limit to maximum grain protein content in high yielding varieties [3-5,12] although there are exceptions [7]. When wheat is grown under cool, well-watered conditions, it is possible to obtain high grain yield together with high protein concentration by supplying nitrogen after anthesis [10,13,14]. However, additional nitrogen alters grain amino acid content and there is a negative relationship between protein concentration and the concentration of essential amino acids such as lysine, cysteine and methionine [12].

There is also a trade-off between grain yield and protein content under conditions of drought and/or high temperature, which reduce accumulation of starch more than protein [10,15]. This is partly because high temperature reduces transcript levels and enzyme activity of starch synthase [15,16]. High protein flours produced under conditions of high temperature were also reported to have increased proportions of glutamine- and proline-rich proteins and reduced proportions of cysteine- and methionine-rich proteins [11]. Understanding the molecular mechanisms that regulate the balance between starch and protein synthesis could aid in efforts to uncouple the inverse relationships between starch and protein synthesis and between desirable amino acid composition versus high protein content.

Grain-fill depends upon import of sugars and amino acids from the leaves and stem. Nutrients are unloaded from the phloem into the endosperm cavity and taken up by endosperm cells in the region of the crease [17]. Carbohydrate supply to the grain is mainly in the form of sucrose [18]. Amino acid supply is mainly as glutamine, alanine, serine and glycine [19,20] and sulfur is imported as sulfate, glutathione and S-methylmethionine [21-23]. Within the endosperm, these molecules are converted into the other sugars, complex carbohydrates, amino acids, nucleic acids, enzymes and structural proteins, lipids, and membranes needed for cell development as well as the abundant starch and storage proteins that will support the embryo upon germination. Molecules synthesized in the endosperm also are transported into the scutellum of the growing embryo [24,25].

Many of the metabolic pathways for synthesis of amino acids, isoprenoids, lipids and other products are located within plastids [26,27], so it is logical that these essential molecules are synthesized within the amyloplasts. At 10 to 12 DPA the amyloplasts are large amorphous organelles. Outer and inner envelopes surround an extensive matrix within which a starch granule gradually enlarges [28,29]. Proteomic studies provided lists of proteins found in fractions highly enriched in these amyloplasts, but did not clearly distinguish between true amyloplast proteins and contaminants from the cytoplasm and other organelles [6,30,31]. However, two of the studies [6,31] suggested that amyloplasts are involved in many metabolic functions in addition to starch production. This paper reduces the number of putative amyloplast proteins in [6] from 90% to 62% of those detected and then organizes them into proposed metabolic and biosynthetic pathways. The enzymes are arranged into maps that include enzyme and compound names in a single view. The paper summarizes the evidence for each pathway and its role in grain development. Links to the original MS/MS data are provided. The analysis should be useful in eventual on-line maps of wheat metabolic enzymes in the manner of AraCyc [32] or RiceCyc [33]. Unlike AraCyc or RiceCyc it summarizes the pathway evidence from the point of view of a single organelle.

## Results and Discussion

### Categorization of proteins in the amyloplast preparation

Analysis of the 288 proteins detected in the amyloplast fraction [6] indicated that 177 originated in amyloplasts (62%) (Tables 1, 2, 3, 4). Also see Additional file 1. Another three proteins of unknown function were equally likely to be from plastid or mitochondrion, and 10 were of unknown function or location. It was determined that the remaining proteins most likely were not amyloplast proteins. The table in Additional file 1 gives the sequence that was the best fit to the tandem MS/MS data for the tryptic peptides from each protein. For the 288 proteins, only 33 best matches were to proteins from the NCBI non-redundant database [34], whereas 255 were contigs from wheat EST databases [35-37]. Although this indicates the excellent coverage of wheat sequences in current EST databases, using these sequences to predict a plastid transit peptide was problematic. Many of the wheat contigs were partial, composed of ESTs from multiple varieties, lacked the coding region for the N-terminal protein sequence, or had other potential problems. It requires considerable analysis to assemble a reliable contig with the best fit for an individual protein, as illustrated in studies of omega gliadins and lipid transfer proteins by Altenbach et al. [38,39]. Therefore, the decision was made to use the predicted peptides for a BLAST [40] search of the NCBI nonredundant database to find the most closely related homolog for which a complete protein sequence could be deduced. For the 180 likely amyloplast proteins, 21% of the closest homologs in the nonredundant database were predicted products of wheat (*Triticum aestivum*) genes or wheat cDNA clones and 68% were the predicted products of rice (*Oryza sativa*) genes. These were analyzed using Target P to predict organellar transit peptides [41]. The analysis does not prove whether the actual wheat protein had a transit peptide, but indicates whether the closest known homolog did.

**Table 1:** Proteins of carbohydrate metabolism that were detected and proposed to be wheat amyloplast constituents^1^

**Carbohydrate metabolism**	**Plastid Criteria**^1^
**Glucose metabolism**	

1 Glucose-6-phosphate isomerase EC 5.3.1.9	1, 2(ch), 3(ch), 4
2 Hexokinase EC 2.7.1.1	2 (ch/cy), 3 (ch/mi), 4

**Glycolysis**	

3 Fructose-bisphosphate aldolase EC 4.1.2.13	1, 2(ch/cy), 3(ch), 4
4 Glyceraldehyde-3-phosphate dehydrogenase EC 1.2.1.12	2(ch/cy), 3(ch), 4
5 Phosphoenolpyruvate mutase-like EC 5.4.2.9	2(ch), 3(ch)
6 Phosphoglycerate dehydrogenase EC 1.1.1.95	1, 2(ch), 3(ch), 4
7 Phosphoglycerate kinase EC 2.7.2.3	1, 2(ch/cy), 3(ch), 4
8 Phosphopyruvate hydratase EC 4.2.1.11	2(ch/cy/mi), 3(mi/ch), 4
9 Pyruvate kinase, putative EC 2.7.1.40	2(ch/cy), 3(ch), 4
10 Triosephosphate isomerase EC 5.3.1.1	2(ch/cy/pl), 3(ch), 4

**Pyruvate dehydrogenase complex**	

11 Dihydrolipoyllysine-residue acetyltransferase EC 2.3.1.12	2(ch/pl), 3(ch), 4
12 Lipoamide dehydrogenase EC 1.8.1.4	2(ch/mi/pl), 3(ch), 4
13 Pyruvate dehydrogenase E1 alpha subunit EC 1.2.4.1	2(ch/mi/pl), 3(ch), 4
14 Pyruvate dehydrogenase E1 beta subunit EC 1.2.4.1	2(ch/mi/pl), 3(ot), 4

**Citrate and malate**	

15 Aconitate hydratase, putative EC 4.2.1.3	2(ch, cy, mi), 3(ch)
16 Malic enzyme (NADP-dependent) EC 1.1.1.39	2(ch, cy, mi), 3(ch), 4

**Starch metabolism**	

17 4-alpha-glucanotransferase EC 2.4.1.25	1, 2(am, ch, cy, pl), 3(ot), 4
18 Alpha 1,4-glucan phosphorylase EC 2.4.1.1	1, 2(am, ch, cy), 4
19 Glucose-1-phosphate adenylyltransferase large subunit (ADP-G) EC 2.7.7.27	1, 2(am, ch, cy), 3(ch), 4
20 Glucose-1-phosphate adenylyltransferase small subunit EC 2.7.7.27	"
21 Glucose-1-phosphate adenylyltransferase, small subunit EC 2.7.7.27	"
22 NDP-glucose-starch glucosyltransferase (granule bound starch synthase) EC 2.4.1.242	1, 2(am), 3(ch), 4
23 1,4-alpha-glucan branching enzyme (starch branching enzyme 2) EC 2.4.1.18	1, 2(am, pl), 4
24 Starch synthase II EC 2.4.1.21	1, 2(am), 3(ch), 4

**Pentose phosphate cycle**	

25 Glucose-6-phosphate dehydrogenase EC 1.1.1.49	2(ch, cy), 3(ch), 4
26 Phosphoribulokinase EC 2.7.1.19	2(ch), 3(ch), 4
27 Ribose-5-phosphate isomerase EC 5.3.1.6	2(ch), 3(ch), 4
28 Ribulose 1,5-bisphosphate carboxylase, large subunit EC 4.1.1.39	1, 2(ch), 3(ot),4
29 Transaldolase, putative EC 2.2.1.2	2(mi, pl), 3(ch), 4
30 Transketolase EC 2.2.1.1	2(ch, cy), 3(ch), 4

**1-Carbon metabolism**	

31 Formate-tetrahydrofolate ligase EC 6.3.4.3	2(ch, cy, mi, pl), 3(ch), 4
32 Glycine hydroxymethyltransferase EC 2.1.2.1	2(ch, cy, mi, pl), 3(ch), 4

^1^Criteria: 1) Known amyloplast or chloroplast protein in wheat; 2) annotated as amyloplast, plastid or chloroplast protein for other plant species in the BRENDA [100] or NCBI [34] databases, or identified as such in a reference in this paper; 3) Target P [41] predicts a plastid transit peptide for the protein product of the wheat gene or homolog; 4) other members of the same pathway or protein complex are known plastid proteins. The following abbreviations are used: am, amyloplast; ch, chloroplast; cy, cytoplasm; en, endomembrane; gl, glyoxysome; mi, mitochondria; nu, nucleus; ot, other; pe, peroxisome; pl, plastid;

**Table 2:** Proteins of amino acid metabolism that were detected and proposed to be wheat amyloplast contsituents^1^

**Amino acid synthesis**	
**Aromatic amino acids**	

33 Anthranilate synthase, beta subunit EC 4.1.3.27	2(ch, cy, pl), 3(ch), 4
34 3-dehydroquinate dehydratase/shikimate dehydrogenase EC 4.2.1.10/EC 1.1.1.25	2(ch), 3(ch), 4
35 3-dehydroquinate synthase EC 4.2.3.4	2(ch), 3(ch), 4
36 3-phosphoshikimate 1-carboxyvinyltransferase EC 2.5.1.19	2(ch), 3(ch), 4
37 Phosphoribosylanthranilate isomerase 1 EC 5.3.1.24	2(pl), 3(ch), 4
38 Tryptophan synthase alpha chain EC 4.2.1.20	2(ch), 3(ch), 4
39 Tryptophan synthase beta subunit EC 4.2.1.20	2(ch), 3(ch), 4

**Aspartate, alanine, lysine and threonine**	

40 Aspartate transaminase EC 2.6.1.1	2 (am, ch, cy), 3(ch), 4
41 Aspartate kinase-homoserine dehydrogenase EC 2.7.2.4/EC 1.1.1.3	2(ch, pl, cy), 3(ch), 4
42 Aspartate-semialdehyde dehydrogenase EC 1.2.1.11	2(ch, mi), 3(ch), 4
43 Diaminopimelate decarboxylase EC 4.1.1.20	2(ch), 3(ch), 4
44 Diaminopimelate epimerase-like protein EC 5.1.1.7	2(ch), 3(ch), 4
45 Dihydrodipicolinate reductase-like EC 1.3.1.26	2(ch), 3(ch), 4
46 Dihydrodipicolinate synthase 1 EC 4.2.1.52	2(ch), 3(ch), 4
47 Threonine synthase EC 4.2.99.2	2(ch), 3(ch), 4

**Branched chain amino acids**	

48 Acetolactate synthase EC 4.1.3.18	2(ch), 3(ch), 4
49 Branched-chain amino acid aminotransferase EC 2.6.1.42	2(ch, cy, mi, pl), 3(ch), 4
50 Dihydroxy-acid dehydratase EC 4.2.1.9	2(ch, mi), 3(ch), 4
51 3-isopropylmalate dehydratase, large subunit EC 4.2.1.33	2(ch), 3(ch, mi), 4
52 3-isopropylmalate dehydratase, small subunit EC 4.2.1.33	2(ch), 3(ch), 4
53 3-isopropylmalate dehydrogenase EC 1.1.1.85	2(ch), 3(ch, mi), 4
54 2-isopropylmalate synthase A EC 2.3.3.13	2(ch), 3(ch), 4
55 Ketol-acid reductoisomerase EC 1.1.1.86	2(ch), 3(ch), 4

**Cysteine and sulfur metabolism, sulfur assimilation**	

56 Cystathionine beta-lyase EC 4.4.1.8	2(ch, cy), 3(ch), 4
57 Cysteine S-conjugate beta-lyase EC 4.4.1.13	2(mi), 3(ch),4
58 Cysteine synthase 1 EC 2.5.1.47	2(ch, cy, mi, pl), 3(ch), 4
59 Glutamate-cysteine ligase EC 6.3.2.2	2(ch, cy), 3(ch, mi), 4
60 Phosphoadenylyl-sulfate reductase EC 1.8.4.8	2(ch), 3(ch), 4
61 Sulfate adenylyl transferase EC 2.7.7.4	2(ch, cy), 3(ch), 4
62 Thiosulfate sulfurtransferase EC 2.8.1.1	2(ch, cy, mi), 3(ch), 4

**Glutamine, glutamate and asparagine**	

63 N-acetyl-gamma-glutamyl-phosphate reductase EC 1.2.1.38	2(ch), 3(ch), 4
64 Acetylglutamate kinase-like protein EC 2.7.2.8	2(ch), 3(ch), 4
65 Acetylornithine aminotransferase EC 2.6.1.11	2(ch, mi), 3(ch), 4
66 Argininosuccinate lyase EC 4.3.2.1	2(ch), 3(ch), 4
67 Argininosuccinate synthase EC 6.3.4.5	2(ch), 3(ch, ot), 4
68 Ferredoxin-dependent glutamate synthase (FD-GOGAT) EC 1.4.7.1	2(ch), 3(ch), 4
69 Ornithine carbamoyltransferase EC 2.1.3.3	2(ch), 3(ch), 4

**Histidine**	

70 ATP phosphoribosyl transferase EC 2.4.2.17	2(ch), 3(ch), 4
71 Histidinol dehydrogenase EC 1.1.1.23	2(ch), 3(ch), 4
72 Imidazoleglycerol phosphate synthase hisHF EC not available	2(ch), 3(ch), 4
73 Phosphoribosyl-ATP diphosphatase EC 3.6.1.31	2(ch), 3(ch), 4
74 1-(5-phosphoribosyl)-5-((5-phosphoribosylamino) methylideneamino) imidazole-4-carboxamide isomerase EC 5.3.1.16	2(ch, mi), 3(ch, mi), 4

^1^Criteria: as in Table 1.

**Table 3:** Proteins involved in synthesis of nucleic acids, porphyrins, isoprenoids, vitamins and fatty acids that were detected and proposed to be wheat amyloplast constituents^1^.

**Nucleic acid synthesis**	
**General**	

75 Inorganic diphosphatase EC 3.6.1.1	2(ch, ot), 3(ch), 4
76 Quinolinate synthase A EC not available	2(ch), 3(ch), 4
77 Nucleoside diphosphate kinase EC 2.7.4.6	2(ch, cy), 3(ch), 4

**Purines**	

78 Adenylate kinase A EC 2.7.4.3	2(ch, cy), 3(ch), 4
79 Adenylosuccinate synthetase EC 6.3.4.4	1,2(ch), 3(ch, mi), 4
80 Phosphoribosylaminoimidazolecarboxamide formyltransferase EC 2.1.2.3	2(ch, mi, nu), 3(ch), 4
81 Phosphoribosylformylglycinamidine cyclo-ligase EC 6.3.3.1	2(ch, cy, mi, pl), 3(ch), 4
82 Phosphoribosylaminoimidazolesuccinocarboxamide synthase EC 6.3.2.6	

**Pyrimidines**	

83 Aspartate carbamoyltransferase EC 2.1.3.2	1, 2(ch), 3(ch, mi), 4
84 Carbamoyl phosphate synthetase, large subunit EC 6.3.5.5	2(ch, cy), 3(ch), 4
85 Carbamoyl phosphate synthase, small subunit, glutamine dependent form EC 6.3.5.5	2(ch, cy), 3(ch), 4
86 Dihydroorotate dehydrogenase EC 1.3.99.11	2(ch, cy), 3(ch), 4

**Porphyrin synthesis**	

87 Coproporphyrinogen oxidase EC 1.3.3.3	2(ch), 3(ch), 4
88 Ferritin (no EC number)	2(ch), 3(ch, mi), 4
89 Ferrochelatase EC 4.99.1.1	2(ch, mi), 3(ch), 4
90 Glutamate-1-semialdehyde 2,1-aminomutase EC 5.4.3.8	2(ch), 3(ch), 4
91 Heme oxygenase 1 EC 1.14.99.3	3(ch), 4
92 Hydroxymethylbilane synthase EC 4.3.1.8	2(ch), 3(ch), 4
93 Porphobilinogen synthase EC 4.2.1.24	2(ch, cy, pl), 3(ch), 4
94 Uroporphyrinogen decarboxylase EC 4.1.1.37	2(ch, pl), 3(ch), 4

**Isoprenoids**	

95 2-C-methyl-D-erythritol 4-phosphate cytidylyltransferase EC 2.7.7.60	2(ch, pl), 3(ch), 4
96 4-hydroxy-3-methylbut-2-en-1-yl diphosphate synthase EC 1.17.4.3	2(ch), 3(ch), 4
97 Isopentenyl diphosphate DELTA DELTA 2 isomerase EC 5.3.3.2	2(ch, mi, pe, pl), 3(ch), 4
98 Phytoene synthase EC 2.5.1.32	2(ch, pl), 3(ch), 4
99 Phytoene desaturase EC 1.14.99.3?	3(ch),4

**Vitamins and cofactors**	

100 Pyridoxamine 5'-phosphate oxidase-related domain containing protein EC 1.4.3.5 (related)	3(ch, mi), 4
101 Riboflavin synthase, alpha chain EC 2.5.1.9	3(ch), 3(ch), 4
102 Thiamine biosynthesis protein ThiC EC not available	2(ch), 3(ch), 4
103 Tocopherol cyclase EC not available	2(ch), 3(ch), 4
104 Tocopherol methyltransferase EC 2.1.1.95	2(ch), 3(ch), 4

**Fatty acid synthesis**	

105 Acetyl-CoA carboxylase EC 6.4.1.2	2(ch, cy, mi, pl), 3(ch), 4
106 Acyl-[acyl-carrier-protein] desaturase EC 1.14.19.2	2(ch, cy), 3(ch), 4
107 [acyl-carrier-protein] S-malonyltransferase EC 2.3.1.39	2(ch, pl), 3(ch), 4
108 Beta-ketoacyl-acyl-carrier-protein synthase I EC 2.3.1.41	2(ch, mi), 3(ch), 4
109 Enoyl-[acyl-carrier-protein] reductase (NADPH, B-specific) EC 1.3.1.10	2(ch), 3(ch), 4
110 3-Hydroxydecanoyl-[acyl-carrier-protein] dehydratase EC 4.2.1.60	2(ch), 3(ch), 4
111 Oleoyl-[acyl-carrier-protein] hydrolase EC 3.1.2.14	2(ch), 3(ot, ch),4
112 3-Oxoacyl-[acyl-carrier-protein] reductase EC 1.1.1.100	2(ch), 3(ch), 4

^1^Criteria: as in Table 1.

**Table 4:** Proteins involved in redox systems, photosystems, signalling, transport, protein synthesis, assembly and turnover, plastid division, or unknown functions that were detected and proposed to be wheat amyloplast constituents^1^.

**Redox systems**	
**Ferredoxin-thioredoxin system**	

113 Ferredoxin III (no EC)	2(ch), 3(ch), 4
114 Ferredoxin-NADP+ reductase EC 1.18.1.2 – non-green isoform	2(ch, pl), 3(ch), 4
115 Ferredoxin-thioredoxin reductase, variable subunit EC not available	2(ch), 3(ch), 4

**Free radical scavenger system**	

116 Ascorbate peroxidase EC 1.11.1.11	2(ch, cy), 3(ch), 4
117 Cu/Zn superoxide dismutase EC 1.15.1.1	2(ch, cy, mi), 3(ch), 4
118 Glutaredoxin EC 1.20.4.1	2(ch), 3(ch), 4
119 Monodehydroascorbate reductase EC 1.6.5.4	2(ch, cy, mi), 3(ch), 4
120 Peroxiredoxin EC 1.11.1.15	2(ch), 3(ot), 4
121 Peroxiredoxin (Type 2) EC 1.11.1.15	2(ch), 3(ch), 4

**Photosystem I and II thylakoid proteins**	

122 ATP synthase alpha chain (encoded by chloroplast DNA) EC 3.6.3.14	2(ch), 3(ot), 4
123 Apocytochrome f precursor (encoded by chloroplast DNA) EC not available	2(ch), 3(ot) but known plastid protein)
124 Chlorophyll a/b binding protein of photosystem II EC not available	2(ch), 3(ch), 4
125 Chlorophyll a/b binding protein of photosystem II EC not available	2(ch), 3(ch), 4
126 23 kDa oxygen evolving protein of photosystem II EC not available	2(ch), 3(ch), 4
127 33 kDa oxygen evolving protein of photosystem II EC not available	2(ch), 3(ch), 4
128 Oxygen-evolving enhancer protein 2 EC not available	2(ch), 3(ch), 4
129 Photosystem I reaction center subunit II EC not available	2(ch), 3(ch), 4

**Signaling**	

130 Inositol phosphate phosphatase EC 3.1.3.25	2(ch), 3(ch), 4
131 Inositol monophosphatase family protein	2(ch), 3(ch, mi) 4

**Substrate transport**	

132 ABC-type transporter, ATPase component, EC 3.6.3.*	2(ch), 3(ch), 4
133 ABC-type transporter, putative EC 3.6.3.*	2(ch), 3(ch), 4
134 ADP-glucose transporter	2(ch, mi), 3(ch, ot) 4
135 Amino acid selective channel protein	2(ch),3(ot),4

**Protein synthesis, import, assembly and turnover**	

**Ribosomal Proteins**	

136 29 kDa ribonucleoprotein A	2(ch), 3(ch), 4
137 40S ribosomal protein S20	2(ch), 3(ch, ot), 4
138 Hypothetical protein OSJNBa0071K18.7 – unk, possible chloroplast ribosomal 52	2(ch), 3(ch, mi), 4
139 Cp31BHv (31 kDa ribonucleoprotein, chloroplast)	2(ch), 3(ch), 4
140 Ribosome recycling factor	2(ch), 3(ch), 4
141 Translational elongation factor Tu	2(ch), 3(ch), 4
142 Translational inhibitor protein, putative	2(ch), 3(ch), 4

**Protein import**	

143 Translocon Tic40-like protein	2(ch), 3(ch), 4
144 Translocon Tic40	2(ch), 3(ot), 4
145 Translocon Tic110	2(ch), 3(ot), 4
146 Translocon Toc34-1	2(ch), 3(ot), 4
147 Translocon Toc75	2(ch), 3(ch), 4
148 Translocon Toc75	2(ch), 3(ch), 4

**Assembly, folding, proteolysis and turnover**	

149 Aminopeptidase C, putative (Acyl-peptide hydrolase-like) EC 3.4.22.40	2(ch), 3(ch), 4
150 Chaperonin, 20 kDa, chloroplast	2(ch), 3(ch), 4
151 Chaperone GrpE	2(ch), 3(ch), 4
152 Clp C protease, ATP-binding subunit EC 3.4.21.92	2(ch, mi), 3(ch, mi), 4
153 Clp C protease, ATP-binding subunit EC 3.4.21.92	
154 ClpB heat shock protein, putative	2(various), 3(ch)
155 Heat shock cognate 90 kDa protein, putative	2(various), 3(ch)
156 Oligopeptidase A-like EC 3.4.24.70 – mito?	2(ch), 3(ch), 4
157 Peptidyl-prolyl isomerase EC 5.2.1.8	2(unk), 3(mi), 4
158 Peptidyl-prolyl isomerase, Cyclophilin-like – mito?	2(ch), 3(ch), 4
159 Peptidyl-prolyl cis-trans isomerase-like protein	2(ch), 3(mi), 4
160 Plastid-lipid associated protein EC not available	2(ch), 3(ch), 4
161 Rubisco subunit binding-protein alpha subunit	2(ch), 3(ot, mi), 4
162 Rubisco subunit binding-protein beta subunit	2(ch), 3(ch), 4
163 Zinc metalloprotease EC 3.4.21.74	2(ch, mi), 3(ot), 4

**Plastid division**	

164 FtsZ protein	2(ch), 3(ch), 4
165 Plastid division protein [Arc6]	2(ch), 3(ch), 4

**Function is unknown or not verified, but probably located in plastid**	

166 Coenzyme F420 hydrogenase-like EC 1.12.99.1	2(pl), 3(ch)
167 Cytochrome b5 domain containing protein	2(ch), 3(ch)
168 Isoflavone reductase related protein EC 1.3.1.45 related	2(ch), 3(ch)
169 Hypothetical protein At1g26160 – putative metal-dependent phosphohydrolase; biological function unknown	2(pl), 3(ch)
170 Hypothetical protein At2g43940	2(ch), 3(ch)
171 Hypothetical protein At5g08540	2(pl), 3(ch)
172 Hypothetical protein F24D7.19	2(ch), 3(ch, mi)
173 Hypothetical protein OSJNBa0038O10.22	2(ch), 3(ch)
174 Hypothetical protein OSJNBa0054L14.15, DUF573 family protein of unknown function	2(unk),3(ch)
175 Hypothetical protein OSJNBa0095E20.4 – CBS domain containing protein; predicted transcriptional regulator. CBS domains are found in otherwise unrelated proteins and enzymes however	2(unk), 3(ch)
176 Hypothetical protein Os06g0217700 – function unknown	2(unk), 3(ch)
177 Hypothetical protein Os08g0254900 – Fiber protein FB4	2(ch), 3(ch)
178 Hypothetical protein Os08g0254900 – Fiber protein FB4 same gene as above but different peptides and different 2DE spot	2(ch), 3(ch)

**Function is unknown or not verified, but may be located in plastid and/or mitochondrion **	

180 Hypothetical protein At3g55760	3(ch, mi)
181 Hypothetical protein P0450E05.20	3(ch, mi)
182 Glycine-rich RNA-binding protein 2, putative	2(mi), 3(ch)

**Probably non-plastid but mentioned in paper**	

183 Phosphoglucomutase EC 5.4.2.2	2(ch, cy), 3(ot),
184 Phosphoglycerate mutase EC 5.4.2.1	2(pl, cy), 3(ot)
185 Lactoylglutathione lyase EC 4.4.1.5	2(cy, mi), 3(ot)
186 6-phosphogluconate dehydrogenase EC 1.1.1.44	2(am, ch, cy, pl), 3(sp)

^1^Criteria: as in Table 1.

The predicted amyloplast proteins were grouped by function, and 112 enzymes were arranged into maps of proposed metabolic and biosynthetic pathways. Protein numbers refer to Tables 1, 2, 3, 4 and Figure 1. Details are in Additional file 2, Figures 2-17. Also see Additional file 3.

**Figure 1:**
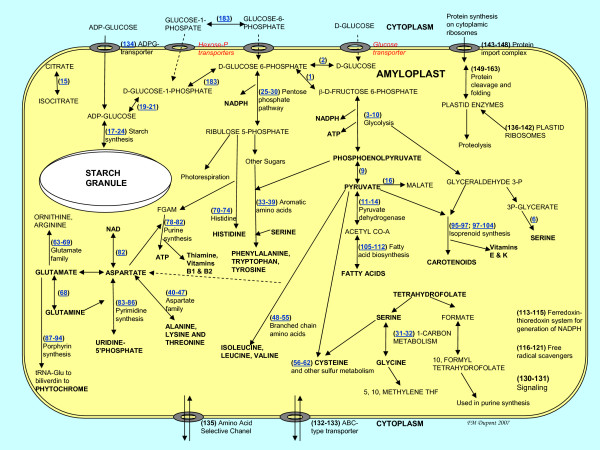
**Summary of metabolic pathways predicted from a proteomic analysis of 10 DPA amyloplasts from the wheat endosperm.** The numbers correspond to the proteins listed in Table 1. Red font indicates that the protein was not detected in the amyloplast preparation. FGAM is formylglycinamidine-ribonucleotide.

In a previous study [42] salt-soluble endosperm proteins were separated from storage proteins by extraction with KCl, and separated from major albumin storage proteins by precipitation with methanol and ammonium acetate. Amyloplast proteins appear to be of low abundance compared to the proteins in the KCl extract, which may be enriched in abundant cytoplasmic enzymes [6]. For comparison with other studies, those identified as thioredoxin targets [31] or found in the KCl extract [42] are indicated (See Additional files 1, 2, 3).

### Carbohydrate metabolism (Proteins #1–32)

Although amyloplasts are known mainly for starch production, this set of 32 enzymes represented only 18% of the amyloplast proteins that were detected (Table 1). Sucrose is converted to fructose and UDP-glucose in the cytoplasm by sucrose synthase and then converted to glucose 1-P and glucose 6-P prior to uptake into plastids [17,26,43,44]. Plastids also take up carbohydrate precursors in the form of triose phosphate, glucose, pentose phosphates, other hexose phosphates, and ADP-glucose [27]. Glucose-6-P and ADP-glucose are the primary source of carbohydrate for wheat amyloplasts, however, and uptake of triose phosphates is less significant for cereal grain amyloplasts than for other types of plastid [27,44]. In addition to enzymes required for starch biosynthesis, most enzymes of glucose metabolism, glycolysis and the pentose phosphate pathway and two of 1-C metabolism were detected.

#### Glucose Metabolism (Proteins 1–2)

Proteins #1 and #2 are enzymes essential to glucose metabolism. Hexokinase (#2) is the entry point for glucose into sugar and starch metabolism. It is reported have an isoform in the chloroplast outer envelope [45], but its role in import or export of glucose-6-phosphate is uncertain. The homolog related to the wheat protein had a low Target P score of 0.39 for plastid. Glucose-6-phosphate isomerase (#1) is at the branch point between starch production, glycolysis, and the pentose phosphate pathway. A plastidic isoform was detected. Two enzymes that convert fructose 6-phosphate to fructose 1,6-phosphate were not detected. Both 6-phosphofructokinase [2.7.1.105] and fructose bisphosphatase [3.1.3.11] have plastid and cytosolic isoforms [43,46]. Fructose bisphosphatase was reported to be missing from endosperm amyloplasts, in agreement with the lack of import of triose phosphates [46]. The other, 6-phosphofructo kinase, was previously identified in the KCl-extract, perhaps as an abundant cytoplasmic form. Either the conversion from fructose 6-phosphate to fructose 1,6-phosphate takes place in the cytoplasm, or the amyloplast enzyme exists but was not detected.

#### Glycolysis (Proteins #3–10)

Seven enzymes of glycolysis were detected along with an enzyme that phosphorylates phosphoenolpyruvate. All seven were predicted to be plastid located. As starch accumulates in the endosperm, O_2 _levels decrease. Therefore, it was suggested that glycolysis is a more significant source of energy in maize endosperm than is mitochondrial oxidation and the TCA cycle, which require O_2 _[47]. Glycolysis might be particularly important in the wheat endosperm, which becomes densely packed with protein as well as starch. Fructose-bisphosphate aldolase (#3) is essential for generating triose sugars. Phosphoglycerate dehydrogenase (#6) and phosphoglycerate kinase (#7) are key to ATP and NADH production. Although phosphoglycerate mutase (#184) was detected, the protein appeared to be the cytoplasmic isoform, based on the Target-P prediction for the most closely related rice gene. It is identified as a plastid or cytosolic enzyme in other plants. If the wheat protein #184 is actually plastidic, then all of the proteins in the pathway from fructose 1,6-phosphate to pyruvate were detected. Lactoyl glutathione lyase (#185) was also detected but predicted to be of cytoplasmic origin. Mechin et al. [47] stress the importance of cytoplasmic pyruvate orthophosphate dikinase [EC 2.7.9.1] in maize endosperm. That enzyme was not found in the amyloplast fraction but was detected in the KCl-extract. Phosphoenolpyruvate mutase (#5) is an enzyme involved in resistance to herbicides.

#### Pyruvate metabolism (Proteins #11–14)

Pyruvate is the starting point for synthesis of alanine and aspartate, branched chain amino acids, isoprenoids, carotenoids and other vitamins, and fatty acids. Proteins #11–14 are components of pyruvate dehydrogenase, the entryway to production of acetyl Co-A for fatty acid biosynthesis [48]. Dihydrolipoyllysine-residue acetyltransferase (#11), lipoamide dehydrogenase (#12) and the pyruvate dehydrogenase E1 alpha (#13) and E1 beta (#14) subunits all have plastidic and mitochondrial isoforms in plants. Target-P predictions for the protein products of 3 of the 4 homologous rice genes indicated a plastid target sequence but the prediction for the pyruvate dehydrogenase E1 beta subunit was "other".

#### Citrate and Malate (Proteins #15–16)

Two proteins were involved in metabolism of citrate and malate. Plants have isoforms of malic enzyme in cytosol, mitochondria and plastids [49]. The malic enzyme (#16) detected was similar to a rice gene product that was annotated as a plastid protein and predicted to have a plastid transit peptide. Malic enzyme (#16) provides NADPH and pyruvate for lipid biosynthesis. The citrate cycle is primarily in mitochondria, but aconitate hydratase or a related protein (#15) had a predicted plastid transit peptide.

#### Starch biosynthesis (Proteins #17–24)

Five enzymes required for starch biosynthesis were detected. The essential substrate for starch formation is ADP-glucose, produced from glucose 1-phosphate and ATP in the cytoplasm and plastid of cereal endosperm cells. Wheat endosperm has cytoplasmic and amyloplast forms of glucose-1-phosphate adenylyltransferase [17,50]. Amyloplast forms of the large (#19) and small subunit (#20–21) were detected. The capacity for cytoplasmic synthesis of ADP-glucose may increase during grain development [26]. A subunit of the putative ADP-glucose transporter (#134) was detected (see below) suggesting that even at 10 DPA the amyloplasts also import ADP-glucose from the cytoplasm. Phosphoglucomutase (#183), which converts glucose 6-phosphate to glucose 1-phosphate, appeared to be the cytosolic isoform, based on a Target P prediction of "other". Enzymes for starch biosynthesis include NDP-glucose-starch glucosyltransferase (granule-bound starch synthase) (#22), 1,4-alpha-glucan branching enzyme 2 (#23), starch synthase II (#24), and 4-alpha-glucanotransferase (disproportionating enzyme) (#17). Basically the above enzymes transfer the glucose residues from ADP-glucose onto simple and branched glucose chains of amylose and amylopectin in a series of steps that includes trimming the chains so that layers of amylopectin interspersed with amylose are deposited onto the growing starch granule (reviewed in [51]). The role of alpha 1,4-glucan phosphorylase (#18) is uncertain. The enzymes for starch biosynthesis were detected in the membrane fraction, which included starch granules [6]. Not all enzymes of starch biosynthesis were detected, however, including starch synthase I, III or IV, starch branching enzyme 1, debranching enzymes, and alpha- and beta-amylases required for starch degradation.

#### Pentose phosphate pathway (Proteins #25–30)

Six proteins were detected in the pentose phosphate pathway that convert glucose 6-phosphate to ribulose 5-phosphate and then to other three-, five- and seven-carbon sugars. Nonoxidative reactions of the pentose phosphate pathway are confined to plastids, and plastids are the only source of ribose 5-phosphate for nucleic acid synthesis [26]. However, isoforms of glucose-6-phosphate dehydrogenase and 6-phosphogluconate dehydrogenase are found in cytosol and plastid. The plastidic form of glucose-6-phosphate dehydrogenase (#25) was detected, but the 6-phosphogluconate dehydrogenase (#186) was predicted to be the cytosolic form. The other enzymes were predicted to be plastidic. Although ribulose 1,5-bisphosphate carboxylase (#28) is mainly associated with CO_2 _fixation in chloroplasts, there are indications that it also plays a role in non-photosynthetic tissue [52,53]. Two additional enzymes of the pentose phosphate pathway, gluconolactonase [EC 3.1.1.17] and ribulose 5-phosphate 3-epimerase [EC 5.1.3.1] were not detected.

#### Folate 1 carbon (1C) metabolism (Proteins #31–32)

Two enzymes crucial to 1C metabolism were identified. In plants, there are 1C pathways in plastids, mitochondria and cytosol. The 1 formate-tetrahydrofolate ligase (#31) and glycine hydroxymethyltransferase (#32) proteins that were detected were predicted to be the plastid isoforms. Transfer of methyl groups by these enzymes is essential for interconversion of glycine and serine and synthesis of purine nucleotides [54]. Other members of the pathway were not detected. Synthesis of tetrahydrofolate in plants requires mitochondrial and cytoplasmic enzymes [55] so it is likely that tetrahydrofolate itself is imported into the plastid, and possibly imported into the grain from the leaves, although little is known about source/sink relations for this compound.

### Amino acid synthesis (Proteins #33–74)

The 42 proteins involved in amino acid synthesis represent 24% of the proteins identified as amyloplast constituents (Table 2). Almost all steps of amino acid biosynthesis occur within plastids [27] with the principal exception of methionine synthesis and some steps in synthesis of cysteine and proline. The high demand for energy and reducing power for amino acid biosynthesis is met by carbohydrate oxidation, including the oxidative pentose phosphate pathway. Enzymes were present for each of the plastidic pathways for biosynthesis of the amino acids, except that no enzymes involved in the metabolism of proline or asparagine were detected.

Although amino acids are imported from leaves, the compositions of the grain phloem sieve tube sap and endosperm cavity differ significantly from that of the endosperm, indicating that synthesis of many amino acids takes place within the endosperm. Seive tube sap contained more glutamine (64%) and serine than any other amino acid [19]. The most abundant amino acids in the endosperm cavity were glutamine (~43%), alanine (~20%), and serine (~13%) [19-21]. Asparagine [20] and glycine [21] were also reported to be abundant. Cysteine and methionine were virtually absent from the sieve tube sap and concentrations of proline and aromatic amino acids were low. Synthesis of proline, leucine, tyrosine, cysteine, and methionine take place largely within the endosperm. Highest rates of net synthesis in the endosperm were reported for proline, leucine, tyrosine, valine, isoleucine and phenylalanine, with an estimate of 170, 89, 42, 48, 44 and 44 nmole day-1, in that order [20].

#### Aromatic amino acids (Proteins #33–39)

All enzymes required for synthesis of the aromatic amino acids tryptophan, tyrosine and phenylalanine from phosphoenol pyruvate and erythrose 4-phosphate are located in the plastid [56]. Six of these enzymes were detected. Three were on the shikimate pathway from phosphoenolpyruvate and erythrosose 4-phosphate to chorismate (#34-36), including the dual-function 3-dehydroquinate dehydratase/shikimate:NADP dehydrogenase (#34) and three on the pathway from chorismate to tryptophan (#33,37–39). The Arabidopsis or rice homologs for these enzymes encode proteins with predicted plastid transit peptides. Four additional enzymes required for synthesis of phenylalanine and tyrosine from chorismate were not detected, even though tyrosine and phenylalanine ranked third and fourth for net synthesis during grain-fill [20]. The shikimate pathway is also the starting point for synthesis of other compounds that contain aromatic rings, many of which are derived from phenylalanine [56].

#### Aspartate family of amino acids (Proteins #40–47)

Eight enzymes in the pathways for biosynthesis of alanine, aspartate, lysine and threonine were detected. Plastid transit peptides were predicted for the homologs from Arabidopsis and rice and for dihydropicolinate synthase (#46), the one protein in this pathway for which a wheat gene has been described. In some grains, asparagine is one of the primary amino acids imported into the endosperm, where it serves as a source of aspartate, an essential intermediate in the synthesis of other amino acids [57]. However, asparaginase [EC 3.5.1.1] was not detected in this study or in [42]. Aspartate is the precursor of threonine and lysine, and is also involved in transamination reactions for production or deamidation of glutamate and glutamine [57]. Six of seven enzymes in the pathway from aspartate to lysine were detected (#41–46), and an additional enzyme in the pathway from aspartate to threonine (#47). Also detected was aspartate transaminase (#40), which catalyzes formation of glutamate from aspartate, and is an essential enzyme in synthesis of alanine. Alanine transaminase [EC 2.6.1.2] was not identified. It was present in the KCl-extract and may be a cytosolic enzyme. Alanine was reported to be present at a higher concentration in the endosperm cavity than in the phloem exudate, suggesting that additional alanine is produced as amino acids are transported into the grain [19].

#### Branched chain family of amino acids (Proteins #48–55)

All seven enzymes in the pathway for biosynthesis of isoleucine, leucine and valine were present, and thiamine is a cofactor in this pathway. Although there are two separate pathways, one from pyruvate and hydroxyl-ethyl-2-thiamine diphosphate to valine and leucine, and one from hydroxyl-ethyl-2-thiamine diphosphate and oxo-butyrate to isoleucine, four of the enzymes are found in both pathways (#48–50, 55). Five proteins were predicted to have plastid target peptide sequences. The prediction for the other two, the large subunit of 3-isopropylmalate dehydratase (#51) and 3-isopropylmalate dehydrogenase (#53), was ambiguous for plastid or mitochondrion.

#### Cysteine, methionine and other sulfur compounds (Proteins #56–62)

Import of sulfur into the grain is largely in the form of glutathione, S-methyl-methionine and sulfate [21,22]. Synthesis of cysteine and methionine takes place in the endosperm and may limit the synthesis of sulfur-rich endosperm proteins, thus affecting flour quality and nutritional value. Seven enzymes involved in biosynthesis and metabolism of cysteine and other S-compounds were detected. Enzymes for cysteine synthesis in plants are located in plastids, mitochondria, and cytoplasm [58]. Two were detected in the amyloplast preparation and predicted to be plastid located. Sulfate adenylyl transferase (#61) adds a sulfate to ATP to create adenylyl-sulfate, and cysteine synthase (#58) is a multienzyme complex that converts acetyl serine and hydrogen sulfide into cysteine. Adenylyl sulfate also is the sulfur donor for synthesis of the sulfo-lipid sulfoquinovosyldiacylglyceride. Although the enzyme 5'-adenylylsulfate reductase [EC 1.8.4.9] is a key enzyme in conversion of sulfate to cysteine, and is known to be a plastid enzyme [58] it was not detected, nor was the other enzyme required for reduction of sulfate, ferredoxin-dependent sulfite reductase [EC 1.8.7.1]. Serine acetyl transferase [EC 2.3.1.30] was not detected. It supplies the carbon backbone for cysteine and is located in plastids, cytoplasm and mitochondrion, but is not abundant and may be rate-limiting for cysteine production [58]. One of three enzymes needed for production of glutathione was detected, glutamate-cysteine ligase (#59). It joins cysteine to glutamate to produce gamma-glutamyl cysteine and had a target prediction of plastid or mitochondrion. Since sulfur is imported as glutathione, one pathway for cysteine production could be from glutathione to glutamate plus cysteine. Anderson and Fitzgerald [21] suggested this is energetically unfavorable, however, and that there may be an alternate route from glutathione to cysteine.

Cystathionine gamma-synthase [EC 2.5.1.48] is a plastid-located enzyme responsible for synthesis of cystathionine, on the pathway to methionine synthesis [58,59], but it was not detected. However, cystathionine beta lyase (#56) was detected. It is responsible for breakdown of cystathionine to pyruvate and homocysteine, a key intermediate in methionine production that is produced only in plastids. Other enzymes of cysteine metabolism that were detected included phosphoadenylyl sulfate reductase (#60) which transfers sulfate to thioredoxin, and cysteine conjugate beta-lyase (#57) which is required for synthesis of other sulfated compounds. Thiosulfate sulfur transferase (#62) is involved in breakdown of cysteine to pyruvate and sulfate. Methionine synthase and S-adenosyl methionine synthase are primarily cytoplasmic and were detected in the KCl-extract but not in the amyloplast preparation.

#### Glycine and serine (Proteins #6,32)

Serine is an abundant import but may also be synthesized in the amyloplast. Phosphoglycerate dehydrogenase (#6), an enzyme in the pathway from glycolysis to serine [60], was the only member of this pathway detected in the amyloplast preparation. The main route for synthesis of glycine is reported to be from serine by the action of glycine hydroxylmethyltransferase (#32) [61], which was detected. Net synthesis of glycine in the endosperm is unnecessary, however [20].

#### Glutamate family (Proteins #63–69)

Seven of nine enzymes in the pathway for biosynthesis of arginine, glutamate, and glutamine were present and predicted to have plastid transit peptides, even though glutamine is one of the major amino acids imported into the endosperm [19] and glutamine uptake was essential for protein production in maize endosperm [62]. Two essential enzymes for interconversion of glutamate, glutamine and aspartate were detected. Ferredoxin-dependent glutamate synthase (#68) is essential for interconversion of glutamate and glutamine, and aspartate transaminase (#40) for interconversion of glutamine and aspartate. The three enzymes in the pathway from glutamate to proline were not detected, even though proline is one of the most abundant endosperm amino acids and little proline is imported. Enzymes of the pathway from ornithine to proline also were not detected. One of these, pyrroline-5-carboxylate reductase [EC 1.5.1.2] is reported to be located both in plastids and in the cytoplasm but these enzymes are reported to be difficult to detect in plants [63].

#### Histidine (Proteins #70–74)

Four of five enzymes in the joint pathway for synthesis of histidine and purine nucleotides were detected (#70, 72–74), along with one of the three enzymes unique to histidine synthesis (#71). Imidazole glycerol phosphate synthase (#72) is at the branch point between histidine and purines. The homologs encoded proteins with predicted plastid target peptides, except that #74 had a prediction of plastid or mitochondrion.

### Nucleic acid biosynthesis (Proteins #75–86)

Twelve enzymes required for nucleic acid biosynthesis were detected (Table 3). Nucleic acids synthesized in the plastid may subsequently be used in the nucleus for synthesis of DNA and RNA. However, adenine nucleotides are also required for energy metabolism and NAD for transfer of reducing power in the plastid. Uridine is used for cytoplasmic processes requiring UTP, such as glycosylation of sugars. Five of eight enzymes required for purine synthesis were detected (#78–82) and three of six required for pyrimidine synthesis (#83–86). There is limited information on the cellular location of enzymes of purine and pyrimidine synthesis in plants. Nucleic acids are produced through de novo and salvage pathway and the enzymes are unstable and not abundant [64]. One report indicates that purine synthesis occurs in plastids and mitochondria [65]. Quinolinate synthase (#76) is one of four required for synthesis of NAD (#76) and was recently described in a study that indicates that the enzymes for synthesis of NAD are located in plastids but probably not abundant [66]. In this study, plastid transit peptides were predicted for 10 of the 12 protein detected, and plastid or mitochondrial transit peptides were predicted for the other two. This suggests that the amyloplast is a center for nucleic acid synthesis.

### Porphyrins (Proteins #87–94)

Seven of ten enzymes in the pathway from glutamyl tRNA to biliverdin were detected (#87, 89–94), as well as ferritin (#88), an iron donor (Table 3). Glutamyl tRNA is the cofactor in the initial conversion of glutamate to aminolevulinate at the start of the pathway for biliverdin biosynthesis [67]. In chloroplasts this pathway is regulated by light and cytokinin [68]. Biliverdin becomes the bilin chromophore that is an essential component of phytochrome. Plastid transit peptides were predicted for all seven enzymes, while there was a prediction of plastid or mitochondrion for the transit peptide of ferritin (#88).

### Isoprenoids, carotenoids and vitamin E (Proteins #95–99, 103–104)

Five of 12 enzymes in the isoprenoid pathway from pyruvate and glyceraldehyde 3-phosphate to carotene were detected, and plastid transit peptides were predicted for the rice homologs of all five (Table 3). The plastid is the site of the non-mevalonate pathway to isopentyl diphosphate and dimethylallyl diphosphate [69] and two of the seven enzymes in that pathway were detected (#95,96) as well as isopentenyl diphosphate isomerase (#97) which interconverts the two essential isoprenoid intermediates. Two essential enzymes for synthesis of geranylgeranyl diphosphate were not detected, but enzymes on the pathways from geranylgeranyl diphosphate to the carotenoids (#98–99) and vitamin E were detected (#103–104) [70,71].

### Other vitamins and cofactors (Proteins #100–102)

Three enzymes involved in vitamin B metabolism were detected (Table 3). Pyridoxamine 5'-phosphate is involved in metabolism of vitamin B6, although the exact function of the "pyridoxamine 5-phosphate oxidase-related domain containing protein" (#100) is not known. Two B vitamins are produced from metabolites generated by the pathways for biosynthesis of nucleic acids. Riboflavin synthase (#101) is a key enzyme in the synthesis of riboflavin (vitamin B2) [72]. Thiamine biosynthesis protein (#102) is an essential enzyme in the synthesis of thiamine (vitamin B1) which is needed for branched chain amino acid synthesis [73].

### Fatty acid biosynthesis (Proteins #105–112)

The complete set of enzymes required for fatty acid biosynthesis was detected (Table 3). Fatty acids are synthesized only in plastids [27], by successive addition of 2-carbon units. This requires ATP and reducing equivalents. Plastids import acetate from the cytoplasm, or use other carbon sources, depending on the plant. Pyruvate dehydrogenase (#11–14) produces acetyl-Co that is converted into malonyl-CoA by acetyl CoA carboxylase (#105) and incorporated into fatty acids through the fatty acid cycle by repetition of the reactions catalyzed by enzymes numbered #107–110 and #112. The first molecule of acetyl CoA is attached to the acyl-carrier protein, and subsequent molecules of malonyl CoA are added to form the fatty acid in two-carbon steps. The acyl-carrier protein was not detected. Subsequently, oleoyl-(acyl-carrier-protein) hydrolase (#111) catalyzes release of the fatty acid from the acyl-carrier-protein complex. An additional enzyme that was detected is oleoyl-(acyl-carrier-protein) desaturase (#106) which is essential for formation of unsaturated fatty acids. Plastid transit peptides were predicted with two exceptions. The Target-P program predicted a mitochondrial transit peptide for the rice gene for acyl-[acyl-carrier-protein] desaturase (#106), and the wheat gene oleoyl-[acyl-carrier protein] hydrolase (#111) was missing the sequence for the N-terminus. A similar Arabidopsis gene for oleoyl-[acyl-carrier protein] hydrolase did have a predicted plastid transit peptide.

### Red-Ox systems (Proteins #113–121)

Nine enzymes were detected that play roles in transfer of reducing equivalents, regulation of protein function through alteration of redox state, and protection from free radicals (Table 4).

#### Ferredoxin-thioredoxin system (Proteins #113–115)

Three proteins required for reduction and oxidation of thioredoxin were detected. All three are known to be located in plastids and had predicted plastid target sequences. The isoform of ferredoxin NADPH reductase (#114) from non-green plastids uses NADPH to reduce ferredoxin, whereas the chloroplast isoform transfers electrons from ferredoxin to generate NADPH [74]. The isoforms operate at a different redox potentials. The nearest homolog to #114 was a rice protein annotated as the "root isozyme", as were related proteins from maize and Arabidopsis, indicating that #114 is the isozyme found in non-green tissue. In amyloplasts NADPH would be generated by glycolysis and the pentose-phosphate pathway. Reduced ferredoxin would then provide reducing power for reduction of sulfite to sulfide in the synthesis of cysteine and for reduction of thioredoxin by ferredoxin-thioredoxin reductase (#115). Thioredoxin was detected in the amyloplast preparation using antibodies against thioredoxin m and reduced thioredoxin was proposed to modulate the activity of a number of amyloplast enzymes by transfer of reducing equivalents [31]. Candidates for enzymes regulated by thioredoxin are indicated in the pathways linked to Figure 1 and Additional file 1. Many are at pathway branch points or are enzymes involved in generation of ATP or reducing power.

#### Free-radical scavenger system (Proteins #116–121)

Six enzymes involved in protection against free radicals were detected (#116–121). All six are known chloroplast proteins and five were predicted to have plastid transit peptides. Superoxide dismutase (#117) converts oxygen radicals to peroxide. Subsequently, ascorbate peroxidase (#116) removes peroxide and generates monodehydrodoascorbate, which is then reduced by monodehydroascorbate reductase (#119) [75,76]. Peroxiredoxins (#120,121) catalyze conversion of peroxide to water by transferring reducing equivalents from thioredoxin or glutaredoxin (#118) to peroxide [75]. Although the presence of a chloroplast glutaredoxin is disputed [77], the Arabidopsis homolog with a good match to the wheat peptide sequences was predicted to have a plastid transit peptide.

### Photosystem I and II thylakoid proteins (Proteins #122–129)

Eight proteins were thylakoid proteins associated with photosystem I and II (Table 4). The three proteins of the oxygen-evolving complex of photosystem II are extrinsic thylakoid proteins found in the thylakoid lumen (#126–128). The ATP synthase subunit (#122) and apocytochrome f (#123) are two of only three proteins in this study that are encoded on the plastid chromosome [78-80]. The six nuclear-encoded proteins were all predicted to have plastid transit peptides, whereas the plastid-encoded proteins had predictions of "other". The thylakoid proteins could indicate contamination with chloroplasts from the green tissue around the endosperm. However, the amyloplast preparation was white, not green, and it is possible that these are actual amyloplast constituents, indicative of the common origin of amyloplasts and chloroplasts.

### Signaling (Proteins #130–131)

Inositol phosphate phosphatase (#130) and inositol monophosphatase (#131) are typically involved in signal transduction although in plants they may also be involved in metabolism of inositol for other purposes. Both had predicted plastid transit peptides and are found in chloroplasts (Table 4).

### Substrate transport (Proteins #132–135)

Protein channels and transporters are required to move molecules in and out of the amyloplast, crossing the outer and inner membranes. Only four were detected, the putative ADP-glucose transporter (#134), two ABC-type transporters (#132, 133) and one amino acid selective channel protein (#135) (Table 4). Two had predicted plastid target peptides and two had predictions of "other", not unexpected for membrane proteins that would be inserted into the envelope membranes rather than transported through them. This is a small number of transport proteins, considering the need to transport minerals such as Pi, Fe and SO_4_-, carbohydrates, folate, amino acids, vitamins and cofactors in and out of the amyloplast [26], and it is likely that many more wheat amyloplast membrane transport proteins will be discovered with improved techniques and increased sequence information. For example, 16 putative plastidic phosphate translocator genes were identified in a study of the Arabidopsis genome [81]. Kleffman et al. [82] identified 118 proteins with potential roles in transport in a proteomics study of Arabidopsis chloroplasts. They discovered many more transport proteins using a shotgun approach than by using the 2D-gel approach of [6]. The proteins and genes for all plastid transport proteins may not have been identified yet, in part because of the difficulty of measuring transport by membrane proteins to verify their function. Thus even the putative ADP-glucose transporter has not actually been proven to transport ADP-glucose [83].

### Protein synthesis, import, assembly and processing (Proteins #136–163)

Twenty-eight proteins were involved in protein synthesis, import assembly, folding, or turnover and represent 16% of the proteins assigned to the amyloplast (Table 4).

#### Ribosomal proteins (Proteins #136–142)

Seven proteins known to be associated with the chloroplast ribosomal complex were detected. All are encoded by nuclear DNA. Five were predicted to have plastid transit peptides, one had a prediction of plastid or mitochondrion, and one of plastid or "other". Plastid ribosomes are necessary for synthesis of proteins encoded by plastid DNA [80]. Only three such proteins were detected in this study (#25, 122, 123).

#### Protein import (Proteins #143–148)

Six proteins form the pores that cross the outer and inner plastid envelopes and facilitate the import of proteins synthesized on cytoplasmic ribosomes. The Toc-like proteins (#146–148) are associated with the outer envelope and the Tic-like proteins (#143–145) with the inner envelope [84,85]. Toc 34 (#146) does not require a transit peptide for insertion into the outer envelope. Transit peptides were predicted for the other five proteins.

#### Assembly, folding, proteolysis and turnover (Proteins #149–163)

Fifteen proteins required for protein synthesis and processing were detected. Ten were predicted to have plastid transit peptides and two to have mitochondrial transit peptides. The other three predictions were plastid/mitochondrion, "other"/plastid and "other". Functions of the chaperones and proteases that were detected in the amyloplast preparation are still being clarified. Nomenclature for this group of proteins can be confusing. The plastid Hsp90 family is equivalent to the Hsp100 family in other systems; some of the same proteins are also referred to as Clp proteins, some are chaperones that are also proteases, and vice versa [86-89]. Hydrolysis of ATP by the ClpC complex (below) is thought to provide energy for import of the proteins [86-88,90]. Binding by chaperones may also provide the driving force for import [81,82].

The Clp proteins form a chaperone complex that helps drive import of proteins into the plastid, cleave the transit peptide, and recycle plastid components [86,89]. The Clp protease complex may be the plastid equivalent of the proteasome [88]. An important role in green plastids is chlorophyll degradation and turnover. In amyloplasts it might be involved in regulating enzyme activity through selective degradation of plastid enzymes [86]. Clp protease holoenzymes in plastids from green and non-green tissue are reportedly composed of an identical set of 11 Clp proteins [88] of which three were identified in the amyloplast preparation (#152–154). The rice and Arabidopsis homologs of proteins #152 and #153 were annotated ClpA-like, but Clp-A is not found in plants. A BLAST search of these homologs against the NCBI green plant database indicated that they were probably members of the ClpC family.

There are only a few records in NCBI for a plant protein sequence matching the putative amino peptidase C, or acyl-peptide hydrolase (#149). These are for a rice gene and an Arabidopsis homolog of bacterial prolyl oligopeptidase. Also, little information was available for the 20 kDa chloroplast chaperonin (#150). The oligopeptidase (#156) cleaves the target peptide into amino acids so they may be recycled [90]. The plastid-lipid associated protein (#160) may function in thylakoid turnover in green plastids. The zinc metallopeptidase (#163) may be involved in removing the targeting peptide from proteins that are imported from the cytoplasm [87,90].

### Plastid division (Proteins #164–165)

Only two proteins involved in plastid division were detected (Table 4). FtsZ (#164) and Arc6 (#165) form part of a ring-like structure that constricts the plastid during division [85,91]. These may be important in pinching off amyloplast sections containing small B-type starch granules that form after the large A-type granules [28].

### Plastid proteins of unknown function (Proteins #166–178)

Only 13 of the predicted amyloplast proteins were of unknown function (Table 4). Of these, peptides from 10 proteins matched gene products from rice or Arabidopsis that were of unknown function but were annotated as plastid proteins in the NCBI database and encoded transit peptides predicted to target the protein to the plastid. The predicted target sequence for #172 was mitochondrial or plastid but the Arabidopsis homolog is annotated as chloroplast. Three proteins (#174–176) were assigned to this category based only on prediction of a plastid transit peptide and presence in the amyloplast fraction. Although five of the unknown proteins had some functional information, it was insufficient to assign them roles.

### Plastid or mitochondrial proteins of unknown function (Proteins #180–182)

Peptides from an additional three proteins (#180–182) matched gene products that were not annotated as to function or cellular location but had transit peptides with good scores for both plastid and mitochondrion (Table 4).

### Missing Proteins

Because of the large dynamic range of protein amount in biological tissues, proteomic studies mainly detect those abundant proteins for which tryptic digestion produces peptides within the optimal size range for analysis [92]. Tissue fractionation improves the range of proteins detected, and many proteins were detected in the amyloplast fraction that were not previously identified in a general extract of salt-soluble proteins. However, the 178 predicted amyloplast proteins are likely to be those that are most abundant and/or amenable to the method of analysis. For example, proteins of 1-C metabolism [54], serine acetyl transferase [58] and enzymes of NAD synthesis [66] are not abundant and are difficult to detect. Few proteins involved in signal transduction were detected, probably because they are less abundant. More transport proteins might be identified by a different technique [82].

Most pathways detected in this study are located exclusively in plastids and are essential for endosperm metabolism, including most steps in synthesis of amino acids, fatty acids, biliverdin and vitamins. Therefore, enzymes required for these pathways are likely to be present in the amyloplast even if they were not detected. For example the enzymes of the chorismate pathway for synthesis of phenylalanine and tryptophan seem to be required, since there is net synthesis of these amino acids in endosperm. In other cases, failure to detect a protein may indicate its absence from amyloplasts, as in the example of fructose bisphosphatase that is not required because wheat amyloplasts do not utilize triose phosphates [46]. Considerable study will be required to map the multi-compartment pathways such as those for sulfur metabolism or for salvage and de-novo synthesis of the nucleic acids.

### Accuracy of the target predictions

In many cases, the enzymes that were detected are from pathways specific to plastids, and the Target P prediction was in agreement with that knowledge. Similarly, in many cases the Target P prediction for a non-plastid compartment was in agreement with general knowledge about proteins determined to be contaminants, such as the storage proteins, Ras-related proteins of the ER and secretory system, and cytoplasmic ribosomal proteins (Additional file 1). In some cases, however, the evidence is not conclusive. Algorithms for prediction of organelle targeting are not perfect, as discussed in detail in [93]. Similar proteins may be targeted to different organelles [94] and in some cases single genes encode proteins targeted to more than one organelle [95]. In addition, genomic or cDNA sequences for many of the wheat proteins were not available, so many target P predictions were based on related genes, mainly from rice. Thus it is possible that a wheat protein was encoded by a gene for a plastid-specific protein but the homolog was predicted to be non-plastid or vice versa. Thus these assignments should be treated with caution.

### Further directions

To obtain higher grain yield with higher protein content and improve yield as temperatures rise it will be important to understand how the interconnected metabolic pathways of the amyloplast are regulated. Barneix [7] suggests that protein accumulation may be limited by remobilization and transport to the grain, and Mechin et al. [47] propose a key role for the cytoplasmic protein, pyruvate orthophosphate dikinase. However, as the carbon, nitrogen and sulfur building blocks that enter the grain are converted to starch and protein, many of the essential metabolic pathways that control these processes are within the amyloplast and may also play a roll in the tradeoff between yield and protein content. A number of key enzymes may be regulated by thioredoxin [31] and other regulatory mechanisms should be explored.

Multiple approaches are needed for further study of endosperm amyloplast development, composition, metabolism and regulation. A proteomics study is valuable in predicting the plastid location of specific gene products. However, this study was limited to the 10 to 12 DPA window in grain development when sufficient endosperm was available, starch granules were small, and little storage protein had accumulated. It is difficult to isolate amyloplasts free of the extensive network of ER used for storage protein biosynthesis, and after 15 DPA the accumulated starch and protein make it difficult to isolate amyloplasts at all. Techniques such as immunochemical localization or targeting of GFP-fusion proteins would be useful to study later stages in grain development and to provide additional proof of amyloplast localization. A detailed metabolomic study of the fluxes through various pathways is essential to evaluate their significance. Gene expression can be followed throughout grain-fill by using the proteomics data to identify candidate genes to follow in a transcriptomic study. A multidimensional metabolic map for the endosperm would take into account key sites of regulation in the amyloplast, cytoplasm, mitochondria and other organelles as grain-fill progresses.

## Conclusion

Cereal endosperm is a heterotrophic tissue that is highly specialized for accumulation of starch and protein. The wheat grain imports a limited assortment of molecular building blocks from the phloem. Subsequently the imported sucrose, glutamine, and a few other amino acids are transformed into the array of amino acids, lipids, nucleic acids and carbohydrates needed to sustain the intense accumulation of starch and protein in the developing grain. Amyloplasts play a central role in this process. Until recently they have been discussed mainly in terms of their role in starch biosynthesis, whereas their role in synthesis of other endosperm components has been ignored. This study presents evidence for the amyloplast location of at least 18 essential metabolic pathways. Early in grain development, the amyloplasts are large organelles with outer and inner envelope membranes surrounding an extensive matrix and a small starch grain. Research has centered on enzymes associated with the starch granule, but we propose that the matrix is also the site of significant metabolic activity during grain fill.

The wheat endosperm produces the starch and protein that become the bread, pasta, and numerous other foods that supply a large percent of the calories in the human diet. Although most genes for plastid proteins are now encoded in the nucleus, they seem to have originated from the original symbiotic organism that became the first chloroplast [78,82,85]. That cyanobacterial symbiont is the ultimate source of many of the key enzymes required for cereal grain food production.

## Methods

Proteomics data were obtained by Balmer et al. [6]. Amyloplasts were prepared from endosperm at 10–12 DPA, separated into soluble and membrane fractions, and analyzed by two-dimensional gel electrophoresis. Tandem MS/MS spectra were used to interrogate three databases of wheat ESTs and contigs [35-37] as well as the NCBI nonredundant green plant database, excluding rice and Arabidopsis [34]. The peptides that were identified by tandem MS/MS are listed in a supplementary table in Balmer et al. [6]. The complete data are now available as two XML files [96]. To view the data, ftp the XML file to your own computer, access the GPM viewer at the GPM web site [97] and use the "view saved xml file" function to browse and find the XML file and open in it in GPM. The protein of interest can be found within the XML file by searching with the sequence identifier given in Additional file 1.

For this paper, the peptides predicted by [6] were used to interrogate the 2007 version of the NCBI nonredundant green plant protein and translated nucleotide databases [34] using the pBLASTn algorithm with a setting of Expect threshold of 20000, word size 2, Matrix Pam 30, Gap cost Existence: 2, Extension: 1 [40]. For each protein, the closest homolog with a complete N-terminal sequence was identified. The Target-P algorithm was used to analyze the first 200 amino acids of the N-terminal sequence to determine whether a transit peptide was predicted [41,98]. Database information about the cellular location of homologs from additional species was also evaluated. The pBLAST algorithm above was used to interrogate the NCBI EST database, and one typical wheat EST representing each protein was selected, unless the gene or cDNA sequence picked from the NCBI nonredundant database was from *Triticum aestivum*.

Plastid enzymes were grouped into metabolic pathways, based on pathways shown in the ExPASy biochemical pathways [99,100], information from BRENDA [101,102], and many references cited in the text. The most recent EC number and preferred enzyme name was assigned based on information in the latest version of BRENDA 2007.1. Protein and gene databases are constantly being updated. To maintain consistency between this paper and the data in [6] the Additional file 1 includes the same number from the Swiss-Prot database [103] that was used in [6]. Some identifications have changed however.

## Abbreviations

DPA: days after anthesis.

## Supplementary Material

Additional file 1 Detailed proteomic data for proteins detected in the amyloplast fraction. The table gives the enzyme names, EC number currently listed as preferred in the BRENDA enzyme database [[101,,102]], identification numbers for the contig or the NCBI gi number for the sequence identified in the original MS/MS study, the Swiss-Prot [[103]] number used in [6], the number of nonredundant peptides identified, the GenBank accession number for the most closely related homolog in the NCBI nonredundant data base [34] for which a complete protein sequence could be deduced, the predicted organelle and score from the Target-P program [41,97], the known cellular location(s) of the protein in plants, and indicates which proteins were detected in an endosperm salt-soluble extract [42] or identified as thioredoxin-binding in [31]. Click here for file

Additional file 2 Figures 2-17. Click here for file

Additional file 3 Figure 2-17. Detailed hypothetical pathways mapping enzymes of biosynthetic pathways in the wheat endosperm amyloplast. Click here for file
