# Supplementary material for: Metabolic pathways of the wheat (Triticum aestivum) endosperm amyloplast revealed by proteomics
Source: BMC Plant Biol. 2008 Apr 17;8:39. doi: 10.1186/1471-2229-8-39 (PMC2383896; doi:10.1186/1471-2229-8-39)
Supplement: Additional file 1 — Detailed proteomic data for proteins detected in the amyloplast fraction. The table gives the enzyme names, EC number currently listed as preferred in the BRENDA enzyme database [[101,,102]], identification numbers for the contig or the NCBI gi number for the sequence identified in the original MS/MS study, the Swiss-Prot [[103]] number used in [6], the number of nonredundant peptides identified, the GenBank accession number for the most closely related homolog in the NCBI nonredundant data base [34] for which a complete protein sequence could be deduced, the predicted organelle and score from the Target-P program [41,97], the known cellular location(s) of the protein in plants, and indicates which proteins were detected in an endosperm salt-soluble extract [42] or identified as thioredoxin-binding in [31]. [file 1471-2229-8-39-S1.pdf]

Supplementary Table. Detailed proteomic data for proteins detected in the amyloplast fraction.

**Organelle, Pathway and protein**[a]	**Enzyme EC**[b]	**Sequences selected based on MS/MS**[c]	**Swiss Prot from [1]**[d]	**Pep-tides**[e] **#**	**Closest NCBI nr accession**[f]	**NCBI nr score**[g]	**Cov-er**[h] **%**	**Cellular location of homo-logs**[i]	**Signal peptide prediction**[j]	**NCBI wheat EST**[k]	**Wheat EST score**[g]	**Mem or sol**[l]	**KCl TRX**[m]
**Plastid**													
**Carbohydrate**													
**Glucose Metabolism**													
Glucose-6-phosphate isomerase	5.3.1.9	*Alb CT18209*	Q6YXI1	18	Os09g0465600	E-0	42	Var_Ch	Os_Ch 0.90	BQ744527	5.00E-141	S	TRX
Hexokinase	2.7.1.1	*Alb CT 17810*	Q8L5G8	2	Zm gene LOC542510	2.00E-06	6	Os, Zm_Ch	Zm_Ch 0.39	CJ603014	5.00E-05	M	
**Glycolysis**													
Fructose-bisphosphate aldolase	4.1.2.13	*Alb CT18237*	Q94JJ0	7	At gene AT2G01140	6.00E-47	32	Var_Ch	At_Ch 0.83	CJ829762	9.00E-47	M/S	TRX
Glyceraldehyde-3-phosphate dehydrogenase	1.2.1.12	*gi|18076096| Mp*	Q8VXD9	3	Os06g0666600	4.00E-26	13	Var_Cy	Os_Ch 0.93	CJ564398	4.00E-25	S	KCl
Phosphoenolpyruvate mutase-like	5.4.2.9	*rs3|CT2306|*	Q7F8Y3	2	Os04g0504600	3.00E-08	8	At_Ch	Os_Ch 0.98	CK210924	1.00E-11	S	
Phosphoglycerate kinase	2.7.2.3	*gi|129915| Ta*	P12782	12	Ta gene LOC543496	4.00E-87	41	Var_Ch	Ta_Ch 0.88			S	KCl TRX
Phosphoglycerate dehydrogenase	1.1.1.95	*rs3|CT15895|*	Q7XMP6	10	Os04g0650800	4.00E-75	24	At_Ch/Mi	Os_Ch 0.77	BQ744478	2.00E-62	M/S	KCl
Phosphopyruvate hydratase	4.2.1.11	*rs1|CT11399|*	Q9C9C4	8	Os09g0375000	4.00E-39	23	Os_Mi At_Ch	Os_Mi/Ch 0.67/0.65	BQ743868	1.00E-40	M/S	KCl TRX
Pyruvate kinase, putative	2.7.1.40	*rs2|CT3438|*	Q8SA22	2	At gene AAM61702	7.00E-07	6	Var_Ch	At_Ch 0.86	CV773338	7.00E-07	M/S	
Triosephosphate isomerase	5.3.1.1	*rs1|CT3111|*	P46225	10	Sc gene P46225	3.00E-110	52	Var_Ch	Sc_Ch 0.78	CJ705429	6.00E-110	M/S	KCl
**Glycolysis - pyruvate dehydrogenase complex**													
Dihydrolipoyllysine-residue acetyltransferase	2.3.1.12	*Alb CT9547*	Q6ZKB1	3	Os08g0431300	1.00E-28	16	At_Pl	Os_Ch 0.94	CJ667455	5.00E-14	M/S	KCl
Lipoamide dehydrogenase	1.8.1.4	*fr3|gnl|UG|Ta_ S16058021*	Q94CN9	15	Os05g0156700	1.00E-144	38	At_Pl	Os_Ch 0.84	CJ951264	3.00E-87	M/S	TRX
Pyruvate dehydrogenase E1, alpha subunit	1.2.4.1	*rs1|CT10296|*	Q7XTJ3	18	Os04g0119400	E-0	65	At_Pl	Os_Ch 0.90	Q838991 (Butte 86)	9.00E-116	M/S	TRX
Pyruvate dehydrogenase E1, beta subunit	1.2.4.1	*fr1|gnl|UG|Ta_ S17898273*	O65087	4	Os cDNA ABF97865	E-37	14	At_Pl	Os_Ot 0.95	BE592015	1.00E-37	M/S	
**Starch Synthesis**													
4-alpha-glucanotransferase (amylomaltase)	2.4.1.25	*rs2|CT3504|*	Q8LI30	2	Ta gene TADPE1	5.00E-14	8	Var_Pl	Ta_mi 0.90			M	
alpha 1,4-glucan phosphorylase	2.4.1.1	*fr1|gnl|UG|Ta_ S16833774*	Q6UZD6	22	Ta gene DPE1	E-0	40	Var_Pl	Ta_Ot 0.56			M	TRX
Glucose-1-phosphate adenylyltransferase, large subunit (ADP-G)	2.7.7.27	gi|1707923| Hv	P12299	21	Ta cDNA P12299	E-0	66	Var_Pl	Ta_Ch 0.91			M/S	KCl TRX
Glucose-1-phosphate adenylyltransferase, small subunit	2.7.7.27	*gi|1707940| Hv*	P55238	10	Ta cDNA AAU50665	4.00E-47	13	Var_Pl	Ta_Ch 0.93			M/S	KCl TRX

Glucose-1-phosphate adenylyltransferase, small subunit	2.7.7.27	*gi|1707943| Vf*	P40392	5	TA cDNA AAF61173 Zm gene LOC541902	6.00E-33	11	Var_Pl	Ta_Ch 0.50			M	
NDP-glucose-starch glucosyltransferase (granule bound starch synthase)	2.4.1.242	*gi|4588609| Ta*	Q9SYU0	11	Ta gene gss1	5.00E-64	43	Var_Pl	Ta_Ch 0.84			M	
1,4-alpha-glucan branching enzyme (starch branching enzyme 2)	2.4.1.18	*gi|13447952| Ta*	Q9FUU7	14	Ta gene Sbe2	7.00E-37	22	Var_Pl	Ta_Ot 0.95			M/S	TRX
Starch synthase II	2.4.1.242	*gi|23476265| Ae tau*	Q8H1Y9	15	Ta gene wSs2a-1	2.00E-83	27	Var_Pl	Ta_Ch 0.91			M/S	
**Pentose phosphate cycle**													
Glucose-6-phosphate 1-dehydrogenase	1.1.1.49	*gi|3334193| So*	O24357	1	Os03g0318500	6.00E-05	2	Var_Ch	Os_Ch 0.91	CA499146	1E-.001	S	TRX
Phosphoribulokinase	2.7.1.19	*rs3|CT32469|*	Q500U9	1	Os12g0601200	2.00E-05	6	Var_Ch	Os_Ch 0.60	CV774650	6.00E-09	S	
Ribose-5-phosphate isomerase	5.3.1.6	*gi|18076096| Mp*	Q6ZEZ1	2	Os07g0176900	0.035	8	Var_Ch	Os_Ch 0.82	CJ565380	0.64	S	
Ribulose bisphosphate carboxylase, large subunit	4.1.1.39	*gi|31087879| Hco*	O20243	3	Ta plastid gene rbcL	2.00E-06	8	Var_Ch	Ta_Ot 0.89			M/S	TRX
Transaldolase	2.2.1.2	*Alb CT18023*	Q8S1Y0	8	Os01g0926300	1.00E-47	29	At_Mi	Os_Ch 0.78	CJ686203	1.00E-30	M/S	
Transketolase	2.2.1.1	*Alb CT16466*	Q9FPB6	18	Os06g0133800	2.00E-133	22	Var_Ch	Os_Ch 0.90	CJ663722	3.00E-149	M/S	TRX
**Citrate and malate**													
Aconitate hydratase, or related protein	4.1.2.3	*rs-2|CT43861|*	Q6YZX6	6	Os02g0125100	2.00E-61	18	At_Ch	Os_Ch 0.91	CJ733361	2.00E-66	S	KCl
Malic enzyme (NADP-dependent)	1.1.1.39	*Alb CT18330*	Q9LDH7	18	Os01g0188400	1.00E-129	42	Os_Ch Var_Mi	Os_Ch 0.91	CJ658978	6.00E-88	S	
**Folate one carbon metabolism**													
Formate-tetrahydrofolate ligase	6.3.4.3	*Alb CT17080*	Q9SPK5	12	Os09g0446800	1.00E-114	25	Var_Cy/Mi/Pl	Os_Ch 0.95	CJ686527	6.00E-111	M/S	KCl
Glycine hydroxymethyltransferase	2.1.2.1	*fr1|gnl|UG|Ta_S17889437*	O23254	7	Os12g0409000 (no N-term); ABA97575 has N-term	3.00E-92	23	Var_Cy/Mi/Pl	Os_Ch 0.92	CK163197	3.00E-99	M/S	
**Amino acid synthesis**													
**Aromatic amino acid synthesis**													
Anthranilate synthase, beta subunit	4.1.3.27	*rs3|CT2302|-2*	Q7XUS2	1	Os04g0463500	E-0.16	3	At_Ch	Os_Ch 0.98	DR752134	2E-0.04	S	
3-dehydroquinate dehydratase/shikimate dehydrogenase	4.2.1.10	*rs2|CT45673|-1*	O65917	3	At gene EMB3004	1.00E-06	4	At_Ch/Cy	At_Ch 0.95	BQ842239	5.00E-10	S	
3-dehydroquinate synthase	4.2.3.4	*rs1|CT23703|-1*	Q69JT8	2	Os09g0539100	3.00E-14	6	At_Ch	Os_Ch 0.96	CN009578 (1 peptide)	3.00E-06	M/S	
3-phosphoshikimate 1-carboxyvinyltransferase	2.5.1.19	*Alb CT17022*	Q93VK6	7	Os06g0133900	2.00E-32	17	Lp_Ch	Os_Ch 0.77	CA500470	2.00E-33	M/S	
Phosphoribosylanthranilate isomerase 1	5.3.1.24	*rs3|CT26254|-1*	Q6ETX4	1	Os02g0266000	E-0.32	4	Var_Ch	Os_Ch 0.95	BT009532	0.005	S	
Tryptophan synthase, alpha chain	4.2.1.20	*rs1|CT42367|-1*	Q6ZL61	7	Os07g0182100	2.00E-52	24	At_Ch	Os_Ch 0.89	BG263682	1.00E-57	S	
Tryptophan synthase, beta subunit	4.2.1.20	*gi|18481702| Sbr*	Q8W0T4	6	Os08g0135900	4.00E-34	10	Var_Ch	Os_Ch 0.92	CJ529221	2.00E-17	S	TRX

**Aspartate, alanine and threonine**													
Aspartate transaminase	2.6.1.1	*Alb CT13349*	Q6VMN8	5	Os03g0299900	3.00E-41	18	At_Ch	Os-Ch 0.96	BJ244307	1.00E-27	M/S	KCl TRX
Aspartate kinase-homoserine dehydrogenase	2.7.2.4 1.1.1.3	*rs1|CT32286|*	Q69LG7	1	At gene AT1G31230	E-0.74	1	Var-Chl	At_Ch 0.47	CA679767	2.8	S	
Aspartate-semialdehyde dehydrogenase	1.2.1.11	*fr1|gnl|UG|Ta_S16058472*	Q93Y73	9	Os03g0760700	7.00E-64	40	At_Ch/Mi	Os_Ch 0.77	CJ625054.	1.00E-39	M/S	
Diaminopimelate decarboxylase	4.1.1.20	*rs3|CT4692|*	Q6ZG77	8	Os02g0440000	3.00E-32	24	At_Ch	Os_Ch 0.96	BF473073	2.00E-33	S	
Diaminopimelate epimerase-like protein	5.1.1.7	*Alb CT9173*	Q9LFG2	5	Os12g0567200	1.00E-21	20	Var Ch	Os_Ch 0.97	CV772371	3.00E-22	M/S	
Dihydrodipicolinate reductase-like	1.3.1.26	*fr2|gnl|UG|Ta_S17987222*	Q67W29	6	Os02g0436400	2.00E-62	32	At_Ch	Os_Ch 0.91	CD902233	4.00E-81	M/S	
Dihydrodipicolinate synthase 1	4.2.1.52	gi|118236| Ta	P24846	3	Ta gene LOC543218	2.00E-08	11	Var Ch	Ta_Ch 0.82			S	
Threonine synthase	4.2.99.2	*Alb CT9525*	Q6L492	5	Os05g0549700	2.00E-69	18	Os_Ch	Os_Ch 0.97	BM140321	2.00E-61	S	TRX
**Branched chain amino acids**													
Acetolactate synthase	4.1.3.18	*gi|28912436| Ta*	Q84U07	24	Ta cDNA AAO53551	E-0	65	Var_Ch	Os_Ch 0.96			M/S	KCl
Branched-chain amino acid aminotransferase	2.6.1.42	*rs3|CT6402|*	Q8H7T7	2	Os03g0106400	1.00E-10	7	Var_Ch	Os_Ch 0.61	CJ638676	8.00E-09	S	
Dihydroxy-acid dehydratase	4.2.1.9	*Alb CT16809*	Q6YZH8	14	Os08g0559600	2.00E-70	21	Var_Ch	Os_Ch 0.79	FGAS065340	8.00E-64	M/S	TRX
3-isopropylmalate dehydratase, large subunit	4.2.1.33	*rs-1|CT41092*	Q6Z702	4	Os03g0655700	7.00E-29	13	Var_Ch	Os_Ch/M 0.77/0.60	CD454413	5.00E-26	M/S	
3-isopropylmalate dehydratase, small subunit	4.2.1.33	*fr3|gnl|UG|Ta_S17989710*	Q6URQ0	9	Os02g0655300	6.00E-88	49	At_Ch	Os_Ch 0.93	CK209560	4.00E-87	M/S	TRX
3-isopropylmalate dehydrogenase	1.1.1.85	*Alb CT16961*	Q7Y096	8	Os03g0655700	4.00E-78	20	At,Os_Ch	Os_ Ch/Mi 0.30/0.32	BQ806458 (Butte 86)	5.00E-86	M/S	
2-isopropylmalate synthase A	2.3.3.13	*fr3|gnl|UG|Ta_S16057908*	O04973	15	Os12g0138900	2.00E-169	37	At_Ch	Os_Ch 0.98	CD905568	4.00E-117	M/S	KCl
Ketol-acid reductoisomerase	1.1.1.86	*Alb CT18634|*	Q8RZF3	17	Os05g0573700	1.00E+00	53	Os_Ch	Os_Ch 0.81	CK162990	1.00E-146	M/S	KCl TRX
**Cysteine, Sulfur metabolism, sulfur assimilation**													
Cystathionine beta-lyase	4.4.1.8	*rs-1|CT12302|*	Q9LWJ5	1	Os06g0175800	7.00E-09	4	At, Os_Ch	Os_Ch 0.97	BG604390	2.00E-10	S	
Cysteine S-conjugate beta-lyase	4.4.1.13	*Alb CT16249*	Q67UZ0	5	Hv gene IDI4	1.00E-28	17	At_Mi	Hv_Ch 0.79	BQ805052. (Butte 86)	4.00E-28	S	
Cysteine synthase 1	2.5.1.47	*Alb CT14538*	Q84SE4	2	Os01g0978100	2.00E-05	6	Os_Ch	Os_Ch 0.94	CJ710950	4.00E-04	M	
Glutamate-cysteine ligase.	6.3.2.2	*Alb CT17186*	Q8GU95	9	Ta gene GSH1 missing N-terminus	6.00E-61	18	Var_Ch/Cy	Os_Ch/Mi 0.67/0.63				KCl
Phosphoadenylyl-sulfate reductase (thioredoxin)	1.8.4.8	*fr2|gnl|UG|Ta_S16058247*	Q6Z4A7	3	Zm gene APRL1	3.00E-31	13	At_Ch	Zm_Ch 0.84	CJ692054	2.00E-31	S	
Sulfate adenylyl transferase	2.7.7.4	*Alb CT18074*	Q84MN8	12	Medicago cDNA ABE89666	8.00E-56	35	Os_Ch	Mt_Ch 0.57	CJ522645	2.00E-40	S	
Thiosulfate sulfurtransferase	2.8.1.1	*rs2|CT19320|*	Q9ZPK0	3	Os12g0608600	3.00E-19	10	Var_Ch, At_Cy/Mi	Os_Ch 0.68	BQ167501	3.00E-07	S	TRX
**Glutamine family**													
N-acetylglutamate kinase-like protein	2.7.2.8	*rs2|CT22420|*	Q949B4	3	Os04g0550500	1.00E-29	13	At _Ch	Os_Ch 0.94	CJ598624	2.00E-28	S	

N-acetyl-gamma-glutamyl-phosphate reductase	1.2.1.38	*fr1|gnl|UG|Ta_ S16058050*	Q6AV34	6	Os03g0617900	8.00E-44	23	At _Ch	Os_Ch 0.40	CJ726895	3.00E-44	S	
Acetylornithine aminotransferase	2.6.1.11	*rs1|CT2846|*	Q688Q8	4	Os05g0129100	1.00E-13	10	At _Ch Os_Mi	Os_Ch 0.78	BQ806879 (Butte 86)	1.00E-16	S	TRX
Argininosuccinate lyase	4.3.2.1	*rs3|CT48274|*	Q9LEU8	8	Os03g0305500	7.00E-63	21	At_Ch	Os_Ch 0.97	BQ743458	3.00E-59	S	TRX
Argininosuccinate synthase	6.3.4.5	*Alb CT17492*	Q9SZX3	9	Os12g0235800; At4G24830	2.00E-29	24	Var_Ch	Os_Ot 0.34; At_Ch 0.97	AL820298	4.00E-34	M/S	KCl
Glutamate synthase (ferredoxin)	1.4.7.1	*gi|121446| Zm*	Q08258	3	Os07g0658400 (incomplete) Ot cDNA BAD30339 (complete)	E.001	2	Var_Ch	Os_Ch 0.79	CJ652330	1E.033	S	
Ornithine carbamoyltransferase	2.1.3.3	*fr-2|gnl|UG|Ta_S 17882259*	Q6YVI0	3	At gene OTC	3.00E-30	14	Var_Ch	At_Ch 0.97	CK161086	3.00E-42	S	TRX
**Histidine**													
ATP phosphoribosyl transferase	2.4.2.17	rs1|CT10151|-	Q8H8D3	4	Os03g0134300	2.00E-05	13	At_Ch	Os_Ch 0.49	FGAS0163 73	3.00E-06	S	
Histidinol dehydrogenase	1.1.1.23	*fr1|gnl|UG|Ta_ S16058452*	Q9LGY8	10	Os01g0232700	1.00E-53	31	Var_Ch	Os_Ch 0.67	CJ714896	8.00E-61	S	
Imidazoleglycerol phosphate synthase hisHF	none	*rs-2|CT29316|-*	Q9SZ30	4	Os03g0256400	4.00E-13	8	Var_Ch	Os_Ch 0.86	CD453914	2.00E-04	S	TRX
Phosphoribosyl-ATP diphosphatase	3.6.1.31	*Alb CT13590*	Q9SDG1	3	Os01g0276500	2.00E-19	13	At_Ch	Os_Ch 0.80	BF473258	6.00E-27		
1-(5-phosphoribosyl)-5- (5-phosphoribosylamino)methylideneamino)imidazole-4-carboxamide isomerase. EC 5.3.1.16	5.3.1.16	*fr2|gnl|UG|Ta_ S16058240*	O82782	7	At gene BBM II partial rice gene Os05g0400800	2.00E-38	27	At_Mi Os_Ch	At_Mi/Ch 0.64/0.63	CJ672507	7.00E-58	S	
**Nucleotide synthesis**													
**General**													
Inorganic pyrophosphatase	3.6.1.1	*fr3|gnl|UG|Ta_ S17885601*	Q6ZGJ8	4	Os02g0768600	8.00E-29	14	At, Os_ Pl	At_Ch 0.56	CJ540344	1.00E-28	M/S	TRX
quinolinate synthetase A	none	*rs2|CT42589|*	Q9FGS4	1	At gene AT5G50210	8.00E-04	2	At_Ch	At_Ch 0.93	CJ690262	2.00E-05	S	
Nucleoside diphosphate kinase	2.7.4.6	*rs2|CT8274|*	Q762A0	6	Os12g0548300	4.00E-53	37	Nt, So_Ch	Os_Ch 0.88	BQ804366 (Butte 86)	5.00E-57	S	KCl
**Purine synthesis**													
Adenylate kinase A	2.7.4.3	Alb CT18143	Q08479	5	Os11g0312400	5.00E-36	30	Os_Ch	Os_Ot/Ch 0.66/0.5	CJ575536	2.00E-39	S	
Adenylosuccinate synthetase	6.3.4.4	*gi|7546404| Ta*	O24396	14	Ta gene LOC543243	9.00E-136	44	Var_Ch	Ta_Mi/Ch 0.73/ 0.69			M/S	
Phosphoribosylaminoimidazolecarboxamide formyltransferase	2.1.2.3	*gi|28412960| Vu*	Q6ZKK5	4	Os08g0206600	3.00E-11	9	At_Ch	Os_Ch 0.90	CJ669686	5.00E-10	S	
Phosphoribosylformylglycinamidine cyclo-ligase	6.3.3.1	*rs1|CT10519|*	Q850Z8	8	Os03g0831500	2.00E-65	29	At_Ch/Cy	Os_Ch 0.83	CJ650030	8.00E-68	M/S	
Phosphoribosylaminoimidazolesuccinocarboxamide synthase	6.3.3.1	Alb CT13327	Q6YXG8	4	Os09g0467100	3.00E-24	9	Os_Ch At_Ch/Cy	Os_Ch 0.86	CJ650030	1.00E-65	M/S	TRX
**Pyrimidine biosynthesis**													

Aspartate carbamoyltransferase	2.1.3.2	*gi|1085650| Ta; replaced by gi:75313092*	Q9S983	3	Ta cDNA AAB31820 (partial); Os08g0248800	4.00E-06	10	At,Ps_Ch	Os_Mi/Ch 0.69/ 0.49			S	
Carbamoyl phosphate synthase, large subunit	6.3.5.5	*rs2|CT34837|*	Q8S1A5	4	Os01g0570700	5.00E-31	7	At_Ch/Cy	At_Ch 0.96	CJ518658	2.00E-29	S	KCl
Carbamoyl phosphate synthase, small subunit, glutamine dependent form	6.3.5.5	*rs1|CT9662|*	Q8L6J8	3	Os02g0708100	1.00E-06	3	At_Ch/Cy	Os_Ch 0.85	CJ657154	1.00E-18	S	KCl
Dihydroorotate dehydrogenase	1.3.99.11	*rs3|CT4034|*	Q8S3J6	2	Os02g0736400	1.00E-13	11	At_Ch/Cy	Os_Ch 0.66	CJ535017	8.00E-15	S	
**Porpyrins**													
Coproporphyrinogen III oxidase	1.3.3.3	*rs2|CT1798|*	Q42840	8	Os04g0610800	1.00E-29	36	Var_Ch	Nt_Ch 0.88	CK161514	1.00E-32	M/S	
Ferritin	none	*rs2|CT4887|*	Q6DQK1	7	Ta cDNA AAW68440	1.00E-73	55	Var_Ch	Ta_Ch/Mi 0.47/0.54				
Ferrochelatase	4.99.1.1	*gi|7437214| Hv*	P42045	6	Os05g0361200	2.00E-19	16	Var_Ch	Os_Ch 0.73	CK206705	8.00E-31	M	
Glutamate-1-semialdehyde 2,1-aminomutase.	5.4.3.8	*rs2|CT1159|*	P18492	8	Os08g0532200	4.00E-32	30	Var_Ch	Os_Ch 0.95	CK206705	4.00E-26	S	
Heme oxygenase 1	1.14.99.3	*rs1|CT3162|*	Q94FW9	2	Os06g0603000	E.068	9	not reported	Os_Ch 0.92	CJ689813.	E-004	S	
Hydroxymethylbilane synthase	2.5.1.61	*gi|19849543| Ta*	Q8RYB1	3	Ta gene LOC543063	3.00E-06	15	Var_Ch	Ta_Ch 0.69	DR740900	3.00E-05	S	
Porphobilinogen synthase	4.2.1.24	rs2|CT14832|	Q42836	6	Os06g0704600	5.00E-29	23	Var_Ch	Os_Ch 0.78	CK161514	5.00E-35	S	
Uroporphyrinogen decarboxylase	4.1.1.37	*rs2|CT8115|*	Q9AXB0	1	Os01g0622300	2.00E-06	3	Var_Ch	Os_Ch 0.86	CJ614868	5.00E-05	S	
**Isoprenoid synthesis**													
2-C-methyl-D-erythritol 4-phosphate cytidylyltransferase	2.7.7.60.	*rs2|CT19230|*	Q9RR90	2	Os gene BAD82245	2.00E-05	8	At_Ch	Os_Ch 0.52	CA633905	3.00E-05	S	
4-hydroxy-3-methylbut-2-en-1-yl diphosphate synthase	1.17.4.3	*rs2|CT744|*	Q6K8J4	2	Os02g0603800	3.00E-09	2	At_Ch	Os_Ch 0.42	CJ690765	6.00E-08	S	
Isopentenyl diphosphate DELTA DELTA 2 isomerase	5.3.3.2	*rs-1|CT6120|*	Q71RX2	5	Os05g0413400	1.00E-47	25	Var_Plas	Os_Ch 0.83	CD863555	7.00E-50	S	
Phytoene synthase	2.5.1.32	*fr3|gnl|UG|Ta_ S16058220*	Q6ET88	6	Os02g0668100	3.00E-44	33	At_Ch	Os_Ch 0.78	CK161427	6.00E-31	M/S	
Phytoene desaturase	1.14.99.3x	*rs1|CT22686|*	Q9ZTN9	1	Os gene Q9ZTN9.	E-0.54	2	Var_Ch	Os_Ch 0.87	CJ610570	9.00E-06	S	
**Vitamin and cofactor synthesis**													
Pyridoxamine 5'-phosphate oxidase-related domain containing protein	1.4.3.5 (related)	*rs2|CT752|*	Q6ZL16	3	Os07g0573800	2.00E-07	9	unk	Os_Ch/Mi 0.64/0.65	CJ603715	2.00E-06	M/S	
Riboflavin synthase, alpha chain	2.5.1.9	*rs3|CT4884|*	Q9SKU8	1	Nt AAQ04061	2.00E-05	7	So_Ch	Nt_Ch 0.88	CK210661	3.00E-06	M	
Thiamine biosynthesis protein ThiC	none	*fr2|gnl|UG|Ta_ S16058347*	Q9AXS1	17	Os03g0679700	4.00E-26	27	At_Ch	Os_Ch 0.57	CV775284.	4.00E-05	M/S	TRX
Tocopherol cyclase	none	*fr2|gnl|UG|Ta_ S13256486*	Q6K7V6	1	Ta gene	2.00E-08	4	Var_Ch	Ta_Ch 0.83			M/S	
Tocopherol O-methyltransferase	2.1.1.95	*CT17368*	Q6ZIK0	10	Ta gene LOC780621	2.00E-133	48	At,Os_Ch	Ta_Ch 0.98			M/S	
**Fatty acid synthesis**													
3-oxoacyl-[acyl-carrier-protein] reductase	1.1.1.100	*rs2|CT2559|*	Q7XMI8	7	Os02g0503500	5.00E-96	16	Var_Ch	Os_Ch 0.88	No hits		M	TRX
beta-ketoacyl-acyl-carrier-protein synthase	2.3.1.41	rs2|CT5103|	Q69YA2	4	Hv gene Kas12	3.00E-28	15	At,Os_Ch	Hv_Ch 0.82	BQ788803.	1.00E-29	S	

<table>
<tr><td>Acetyl-CoA carboxylase</td><td>6.4.1.2</td><td>gi|7438101|</td><td>O48959</td><td>3</td><td>Ta gene LOC543414</td><td>2.00E-06</td><td>1</td><td>Var_Ch/Cy</td><td>Ta_Ch 0.93</td><td></td><td></td><td>S</td><td>TRX</td></tr>
<tr><td>Oleoyl-[acyl-carrier protein] hydrolase</td><td>3.1.2.14</td><td>rs3|CT16680|-5</td><td>Q8L6B1</td><td>6</td><td>Ta gene fatA missing N-terminus AT4G13050 has N-terminus</td><td>2.00E-17</td><td>18</td><td></td><td>Ta_Ot 0.74
At_Ch 0.87</td><td></td><td></td><td>S</td><td></td></tr>
<tr><td>3-Hydroxydecanoyl-[acyl-carrier-protein] dehydratase</td><td>4.2.1.60</td><td>fr3|gnl|UG|Ta_ S13256486</td><td>Q6I5L0</td><td>4</td><td>Os05g0435700</td><td>6.00E-22</td><td>29</td><td>At_Ch</td><td>Os_Ch 0.78</td><td>CD936803</td><td>3.00E-36</td><td>M/S</td><td></td></tr>
<tr><td>Enoyl-[acyl-carrier-protein] reductase (NADPH, B-specific)</td><td>1.3.1.10</td><td>Alb CT14929</td><td>Q6H5J0</td><td>7</td><td>Os08g0327400</td><td>7.00E-71</td><td>34</td><td>Var_Ch</td><td>Os_Ch 0.94</td><td>J675426</td><td>2.00E-63</td><td>M/S</td><td></td></tr>
<tr><td>[acyl-carrier-protein] S-malonyltransferase</td><td>2.3.1.39</td><td>rs2|CT8692|</td><td>Q8RU07</td><td>5</td><td>At gene AT2G30200</td><td>1.00E-47</td><td>25</td><td>Var_Ch</td><td>At_Ch 0.82</td><td>CJ575376</td><td>3.00E-49</td><td>M/S</td><td></td></tr>
<tr><td>Acyl-[acyl-carrier-protein] desaturase</td><td>1.14.19.2</td><td>rs-1|CT16308|-3</td><td>Q8S059</td><td>6</td><td>Os01g0919900</td><td>2.00E-58</td><td>24</td><td>At,Rc_Ch</td><td>Os_Mi/Ch 0.85/0.33</td><td>CJ685947</td><td>1.00E-63</td><td>M/S</td><td></td></tr>
<tr><td>Red-ox systems</td><td></td><td></td><td></td><td></td><td></td><td></td><td></td><td></td><td></td><td></td><td></td><td></td><td></td></tr>
<tr><td>Ferredoxin-Thioredoxin System</td><td></td><td></td><td></td><td></td><td></td><td></td><td></td><td></td><td></td><td></td><td></td><td></td><td></td></tr>
<tr><td>Ferredoxin III</td><td>none</td><td>gi|119958 Zm|</td><td>P27788</td><td>1</td><td>Os03g0835900</td><td>1.00E-06</td><td>9</td><td>Var_Ch</td><td>Os_Ch 0.90</td><td>CJ551981</td><td>2.00E-05</td><td>S</td><td></td></tr>
<tr><td>Ferredoxin-NADP reductase</td><td>1.18.1.2</td><td>rs3|CT8894|-7</td><td>Q41736</td><td>9</td><td>Os03g0784700</td><td>6.00E-60</td><td>32</td><td>Var_Ch</td><td>Os_Ch 0.87</td><td>DR736456</td><td>2.00E-60</td><td>S</td><td>TRX</td></tr>
<tr><td>Ferredoxin-thioredoxin reductase, variable subunit</td><td>none</td><td>rs2|CT10845|-3</td><td>P80680</td><td>2</td><td>Os04g0528800</td><td>8.00E-09</td><td>17</td><td>Os_Ch</td><td>Os_Ch 0.94</td><td>CV782292</td><td>4.00E-03</td><td>S</td><td></td></tr>
<tr><td>Free-radical scavenger system</td><td></td><td></td><td></td><td></td><td></td><td></td><td></td><td></td><td></td><td></td><td></td><td></td><td></td></tr>
<tr><td>Ascorbate peroxidase</td><td>1.11.1.11</td><td>rs1|CT8244|</td><td>Q7XJ02</td><td>3</td><td>Os04g0434800</td><td>9.00E-25</td><td>14</td><td>Var_Ch</td><td>Os_Ch 0.89</td><td>AAS80158*</td><td>2.00E-22</td><td>S</td><td>KCl</td></tr>
<tr><td>Cu/Zn superoxide dismutase</td><td>1.15.1.1</td><td>gi|7433318|</td><td>O24400</td><td>4</td><td>Ta gene SOD1.1</td><td>2.00E-61</td><td>38</td><td>Var_Ch</td><td>Ta_Ch 0.70</td><td></td><td></td><td>S</td><td></td></tr>
<tr><td>Glutaredoxin</td><td>1.20.4.1</td><td>Alb CT12247</td><td>Q84Z96</td><td>5</td><td>Os08g0565800</td><td>2.00E-30</td><td>36</td><td>Var_Ch</td><td>At_Ch 0.85</td><td>CJ666204</td><td>3.00E-42</td><td>M/S</td><td></td></tr>
<tr><td>Monodehydroascorbate reductase</td><td>1.6.5.4</td><td>rs-2|CT23027|-1</td><td>Q84PW3</td><td>1</td><td>Os08g0151800</td><td>4.00E-06</td><td>3</td><td>Var_Ch/Mi</td><td>Os_Ch 0.82</td><td>BT009531</td><td>4.00E-05</td><td>S</td><td></td></tr>
<tr><td>Peroxiredoxin</td><td>1.11.1.15</td><td>rs2|CT3383|-7</td><td>O81480</td><td>8</td><td>Ta gene TSA-WHEAT</td><td>2.00E-116</td><td>70</td><td>Var_Ch</td><td>Ta_Ot 0.69</td><td></td><td></td><td>S</td><td>TRX</td></tr>
<tr><td>Peroxiredoxin (Type 2)</td><td>1.11.1.15</td><td>rs3|CT14844|</td><td>Q7F8S5</td><td>2</td><td>Os06g0625500</td><td>8.00E-08</td><td>13</td><td>At_Ch</td><td>Os_Ch 0.96</td><td>CJ684095</td><td>3.00E-07</td><td>S</td><td></td></tr>
<tr><td>Photosystem I and II thylakoid proteins</td><td></td><td></td><td></td><td></td><td></td><td></td><td></td><td></td><td></td><td></td><td></td><td></td><td></td></tr>
<tr><td>Apocytochrome f precursor</td><td>none</td><td>gi|7524647| Pinus thunbergia</td><td>P14619</td><td>2</td><td>Ta plastid gene petA</td><td>9.00E-11</td><td>12</td><td>Ta_Ch</td><td>Ta_Ot 0.52</td><td></td><td></td><td>S</td><td></td></tr>
<tr><td>ATP synthase alpha chain</td><td>3.6.3.14</td><td>gi|14017569| Ta</td><td>P12112</td><td>10</td><td>Ta plastid gene atpA</td><td>2.00E-98</td><td>33</td><td>Ta_Ch</td><td>Ta_Ot 0.98</td><td></td><td></td><td>M/S</td><td></td></tr>
<tr><td>Chlorophyll a/b binding protein</td><td>none</td><td>rs3|CT5687|-14; Alb contig CT18112</td><td>Q9SDM1</td><td>4</td><td>Os06g0320500</td><td>6.00E-37</td><td>30</td><td>Var_Ch</td><td>Os_Ch 0.56</td><td>CV776045</td><td>2.00E-42</td><td>M</td><td></td></tr>
<tr><td>23kDa oxygen evolving protein of photosystem II</td><td>none</td><td>Alb CT13790</td><td>Q9LHE5</td><td>3</td><td>Ta gene PsbP 23kDa</td><td>3.00E-25</td><td>17</td><td>Ta_Ch</td><td>Ta_Ch 0.45</td><td></td><td></td><td>M</td><td></td></tr>
<tr><td>23kDa oxygen evolving protein of photosystem II</td><td>none</td><td>gi|131394| Ta</td><td>Q00434</td><td>8</td><td>Ta gene PsbP 23kDa</td><td>9.00E-119</td><td>61</td><td>Ta_Ch</td><td>Ta_Ch 0.45</td><td></td><td></td><td>M</td><td></td></tr>
<tr><td>33kDa oxygen evolving protein</td><td>none</td><td>rs1|CT3806|-10</td><td>Q943W1</td><td>11</td><td>Ta gene PsbO</td><td>2.00E-119</td><td>53</td><td>Var_Ch</td><td>Ta_Ch 0.84</td><td></td><td></td><td>M</td><td></td></tr>
<tr><td>Photosystem I reaction center subunit II</td><td>none</td><td>rs2|CT770|-4; gi|548603|</td><td>P36213</td><td>1</td><td>Os08g0560900</td><td>9.00E-09</td><td>8</td><td>Var_Ch</td><td>Os_Ch 0.69</td><td>CJ569824</td><td>2.00E-07</td><td>M</td><td></td></tr>
</table>

<table>
<tr><td>Signaling</td><td></td><td></td><td></td><td></td><td></td><td></td><td></td><td></td><td></td><td></td><td></td><td></td><td></td></tr>
<tr><td>Inositol phosphate phosphatase</td><td>3.1.3.25</td><td>fr2|gnl|UG|Ta_ S16058157</td><td>Q6Z4I1</td><td>5</td><td>Os07g0191000</td><td>1.00E-42</td><td>20</td><td>Var_Ch</td><td>Os_Ch 0.95</td><td>CJ656407</td><td>2.00E-21</td><td>S</td><td></td></tr>
<tr><td>Inositol monophosphatase family protein</td><td>3.1.3.25</td><td>rs3|CT14606|</td><td>Q94F00</td><td>2</td><td>Os02g0169900</td><td>2.00E-22</td><td>10</td><td>At_Ch</td><td>Os_Ch/Mi 0.87/0.75</td><td>BI479906</td><td>2.00E-23</td><td>S</td><td></td></tr>
<tr><td>Substrate transport</td><td></td><td></td><td></td><td></td><td></td><td></td><td></td><td></td><td></td><td></td><td></td><td></td><td></td></tr>
<tr><td>ABC-type transporter, ATPase component</td><td>none</td><td>rs3|CT8040|-4</td><td>Q9CAF5</td><td>8</td><td>Os03g0332700</td><td>6.00E-42</td><td>23</td><td>Var_Ch</td><td>Os_Ch 0.98</td><td>CV764265</td><td>2.00E-64</td><td>S</td><td></td></tr>
<tr><td>ABC-type transporter, putative</td><td>none</td><td>fr2|gnl|UG|Ta_ S16058174</td><td>Q941V2</td><td>1</td><td>Os01g0830000</td><td>1.00E-10</td><td>4</td><td>Var_Ch</td><td>Os_Ch 0.95</td><td>CJ595613</td><td>3.00E-12</td><td>S</td><td></td></tr>
<tr><td>ADP-glucose transporter</td><td>none</td><td>fr1|gnl|UG|Ta_ S16058484</td><td>Q6E5A5</td><td>14</td><td>Os02g0202400</td><td>7.00E-142</td><td>56</td><td>Var_Ch/Mi</td><td>Os_Ot 0.54</td><td>AL813381</td><td>6.00E-113</td><td>M/S</td><td></td></tr>
<tr><td>Amino acid selective channel protein</td><td>none</td><td>rs-2|CT2019|-3</td><td>O82688</td><td>3</td><td>Os05g0111200</td><td>6.00E-17</td><td>29</td><td>Hv_Ch</td><td>Os_Ot 0.78</td><td>BQ805756</td><td>3.00E-16</td><td>M</td><td></td></tr>
<tr><td>Protein synthesis, import, assembly and</td><td></td><td></td><td></td><td></td><td></td><td></td><td></td><td></td><td></td><td></td><td></td><td></td><td></td></tr>
<tr><td>Ribosomes</td><td></td><td></td><td></td><td></td><td></td><td></td><td></td><td></td><td></td><td></td><td></td><td></td><td></td></tr>
<tr><td>29 kDa ribonucleoprotein A</td><td>none</td><td>rs3|CT3850|</td><td>Q8LHN4</td><td>4</td><td>Os07g0631900</td><td>2.00E-60</td><td>61</td><td>At,Os_Ch</td><td>Os_Ch 0.89</td><td>CJ723388</td><td>8.00E-65</td><td>M/S</td><td></td></tr>
<tr><td>40S ribosomal protein S20</td><td>none</td><td>rs1|CT1106|-10 and Alb CT12496</td><td>P35686</td><td>3</td><td>Os06g0134000</td><td>5.00E-08</td><td>33</td><td>Os_Ch</td><td>Os_Ot/Ch 0.57/0.43</td><td>BG313017</td><td>9.00E-13</td><td>M</td><td>KCl</td></tr>
<tr><td>Hypothetical protein Os10g0463800</td><td>none</td><td>CT13923</td><td>Q8H916</td><td>4</td><td>Os10g0463800</td><td>3.00E-25</td><td>19</td><td>At_Ch</td><td>Os_Ch/Mi 0.86/0.48</td><td>CJ594881</td><td>9.00E-26</td><td>S</td><td></td></tr>
<tr><td>Cp31BHv (31 kDa ribonucleoprotein, chloroplast).</td><td>none</td><td>rs1|CT5131|</td><td>O81988</td><td>2</td><td>Ta gene LOC543186</td><td>5.00E-15</td><td>14</td><td>Var_Ch</td><td>Ta_Ch 0.80</td><td>BQ806098</td><td>1.00E-13</td><td>S</td><td></td></tr>
<tr><td>Ribosome recycling factor</td><td>none</td><td>Alb CT10441</td><td>Q6YTV7</td><td>3</td><td>Os07g0570700</td><td>4.00E-32</td><td>20</td><td>Var_Ch</td><td>Os_Ch 0.90</td><td>CJ710345</td><td>1.00E-33</td><td>S</td><td></td></tr>
<tr><td>Translational elongation factor Tu</td><td>none</td><td>rs3|CT8439|</td><td>Q8W2C3</td><td>4</td><td>Os DNA AAF15312</td><td>4.00E-15</td><td>13</td><td>Var_Ch</td><td>Os_Ch 0.98</td><td>CJ721551</td><td>2.00E-16</td><td>S</td><td></td></tr>
<tr><td>Translational inhibitor protein, putative</td><td>none</td><td>rs3|CT63|</td><td>Q8H4B9</td><td>10</td><td>Os07g0516200</td><td>2.00E-98</td><td>69</td><td>At_Ch</td><td>Os_Ch 0.54</td><td>CJ568582</td><td>1.00E-91</td><td>M/S</td><td></td></tr>
<tr><td>Protein import</td><td></td><td></td><td></td><td></td><td></td><td></td><td></td><td></td><td></td><td></td><td></td><td></td><td></td></tr>
<tr><td>Translocon Tic40-like protein, inner envelope</td><td>none</td><td>rs2|CT8112|</td><td>Q7XQG5</td><td>3</td><td>Os04g0439900</td><td>4.00E-20</td><td>9</td><td>Var_Ch</td><td>Os_Ch 0.94</td><td>CV776314</td><td>5.00E-25</td><td>M/S</td><td></td></tr>
<tr><td>Translocon Tic40, inner envelope</td><td>none</td><td>rs-1|CT49334|</td><td>Q7XQG8</td><td>4</td><td>Os04g0439900</td><td>3.00E-46</td><td>15</td><td>Var_Ch</td><td>Os_Ch 0.94</td><td>CJ943213</td><td>3.00E-54</td><td>M/S</td><td></td></tr>
<tr><td>Transloccon Tic 110, inner envelope</td><td>none</td><td>rs2|CT36440|</td><td>O24293</td><td>3</td><td>Os10g0492300</td><td>5.00E-14</td><td>9</td><td>Var_Ch</td><td>Os_Ch 0.51</td><td>BF484090</td><td>8.00E-23</td><td>M/S</td><td></td></tr>
<tr><td>Translocase Toc34-1, Outer envelope</td><td>none</td><td>fr-2|gnl|UG|Ta_S 17898259</td><td>Q9SBX0</td><td>7</td><td>Os03g0240500</td><td>2.00E-81</td><td>17</td><td>Os, Zm_Ch</td><td>Os_Ot 0.40</td><td>CK170428</td><td>9.00E-101</td><td>M</td><td></td></tr>
<tr><td>Translocase Toc 75 outer envelope</td><td>none</td><td>rs2|CT14373|</td><td>Q84Q83</td><td>9</td><td>Os03g0271200</td><td>2.00E-89</td><td>15</td><td>Var_Ch</td><td>Os_Ch 0.94</td><td>BU100477</td><td>9.00E-95</td><td>M/S</td><td></td></tr>
<tr><td>Translocase Toc 75 outer envelope</td><td>none</td><td>Alb CT2202</td><td>Q9STE8</td><td>5</td><td>Os03g0271200</td><td>6.00E-60</td><td>9</td><td>At,Ps,Os_Ch</td><td>Os_Ch 0.94</td><td>CJ653827</td><td>1.00E-58</td><td></td><td></td></tr>
<tr><td>Assembly, folding, proteolysis and turnover</td><td></td><td></td><td></td><td></td><td></td><td></td><td></td><td></td><td></td><td></td><td></td><td></td><td></td></tr>
<tr><td>Aminopeptidase C</td><td>3.4.22.40</td><td>Alb CT5152</td><td>Q69Y12</td><td>1</td><td>Os06g0215300</td><td>0.56</td><td>1.5</td><td>Var_Ch</td><td>Os_Ch 0.69</td><td>CA688590</td><td>0.49</td><td>S</td><td></td></tr>
<tr><td>Chapereonin, 20 kDa, chloroplast</td><td>none</td><td>rs3|CT4686|</td><td>Q69Y99</td><td>12</td><td>Os02g0781400</td><td>3.00E-92</td><td>65</td><td>Var_Ch</td><td>Os_Ch 0.82</td><td>CJ604193</td><td>3.00E-123</td><td>M/S</td><td></td></tr>
<tr><td>Chaperone GrpE</td><td>none</td><td>fr1|gnl|UG|Ta_ S16198688</td><td>Q6K635</td><td>7</td><td>Os02g0612000</td><td>4.00E-57</td><td>33</td><td>At_Ch</td><td>Os_Ch 0.97</td><td>CD866256</td><td>3.00E-72</td><td>S</td><td></td></tr>
<tr><td>ClpB, heat shock protein, putative</td><td>3.4.21.92</td><td>gi|9651530| Pl</td><td>Q75GT3</td><td>6</td><td>Os03g0426900</td><td>3.00E-28</td><td>13</td><td>Var_Cy,Ch</td><td>Os_Ch 0.58</td><td>BQ168080</td><td>5.00E-28</td><td>S</td><td>TRX</td></tr>
</table>

ClpC protease, ATP-binding subunit (No ClpA in plants)	3.4.21.92	*gi|399213| Sl*	P31542	22	Os12g0230100	5.00E-129	41	Os-Ch/Mi	Os_Ch/Mi 0.50/0.50	BQ295032	3.00E-100	M/S	TRX
ClpC protease, ATP-binding subunit (No ClpA in plants)	3.4.21.92	*rs1|CT34673|*	Q7XL03	2	At gene ERD1	9.00E-09	3	At_Ch	At_Ch 0.97	BU099342	7.00E-16	M	
Heat shock cognate 90 kDa protein, putative	3.4.21.92	*gi|1076758| Sc*	Q43638	16	Os08g0487800	4.00E-103	33	At_Var	Os_Ch 0.90	CJ621943	1.00E-83	S	
Oligopeptidase A-like	3.4.24.70	*Alb CT16916*	Q6K9T1	11	Os02g0830100	4.00E-83	15	At_Ch	Os_Ch 0.84	CA497946	3.00E-94	M/S	
Peptidylprolyl isomerase	5.2.1.8	*rs-1|CT4165|*	Q7X7E8	3	Ta gene LOC542967, no N-term Os06g0708500	2E-25; 1E-22	Os 20	Ta_ER	Os_Mi 0.82			S	
Peptidylprolyl isomerase, cyclophilin-like	5.2.1.8	*rs1|CT2427|*	Q6XPZ6	7	Ta cDNA AAP44535	2.00E-78	52	Os_Ch	Ta_Ch 0.91			M/S	KCl TRX
Peptidyl-prolyl cis-trans isomerase-like protein	5.2.1.8	*fr3|gnl|UG|Ta_S17986625*	Q69WA8	4	Os07g0687500	9.00E-17	18	At_Ch	Os_Mi 0.78	No hits		S	
Plastid-lipid associated protein	none	*fr-1|gnl|UG|Ta_S17988208*	Q8W3I2	4	Os10g0575700	2.00E-42	17	Var_Ch	Os_Ch 0.94	CJ720990	3.00E-59	M	
Rubisco subunit binding-protein, alpha subunit	none	*rs1|CT11106|*	Q7X9A7	17	Os03g0859600	E-0	59	Var_Ch	Os_Ot/Ch 0.73/0.36	CA501712	8.00E-142	M/S	TRX
Rubisco subunit binding-protein, beta subunit	none	*rs2|CT633|*	Q43831	28	Os06g0114000	E-0	75	Var_Ch	Os_Ch 0.95	CK166615	2.00E-173	M/S	TRX
Zinc metalloprotease	3.4.21.74	*rs2|CT7819|*	Q8RUN6	1	Os02g0761400	1.00E-09	18	At_Ch/Mi	Os_Other 0.86	CD490840	3.00E-08	S	
**Plastid division**													
FtsZ protein	none	*rs3|CT2333|*	Q9SDW5	2	Os03g0646100	2.00E-12	7	Var_Ch	Os_Ch 0.90	CJ506346	4.00E-11	S	
Plastid division protein [Arc6]	none	*rs3|CT43125|*	Q7PC78	1	Os02g0122400	2.00E-06	2	Var_Ch	Os_Ch 0.96	CD903230	3.00E-08	M/S	
**Function is unknown or not verified;**													
**protein is probably located in the amyloplast**													
Coenzyme F420 hydrogenase-like	1.12.99.1	*rs1|CT2221|*	Q7XTG7	2	Blast of the wheat EST gives Os04g0320100		unk	Os_Pl	Os_Ch 0.96	CJ703050	2.00E-06	M	
Cytochrome b5-like heme/steroid binding domain containing protein	none	*fr1|gnl|UG|Ta_S16265227*	Q9FVZ9	6	Os10g0502500	2.00E-46	31	Var_Ch	Os_Ch 0.42	CV773691	2.00E-58	M	
Isoflavone reductase related protein	1.3.1.45	*rs-2|CT27818|*	Q8H124	2	Blast of the wheat EST gives Os06g0360300	5.00E-36	unk	At_Ch	Os_Ch 0.77	BQ162226	1.00E-19	M	
Hypothetical protein At1g26160 putative metal-dependent phosphohydrolase	none	*fr1|gnl|UG|Ta_S16260984*	Q93ZV1	7	AT1G26160	3.00E-67	40	Var-Pl	At_Ch 0.88	CJ660806	7.00E-80	S	
Hypothetical protein At2g43940	none	*rs-2|CT32369|*	O80564	3	Os01g0338600	4.00E-25	15	At_Ch	Os_Ch 0.92	CO349434	6.00E-24	S	
Hypothetical protein At5g08540	none	*Alb CT16131*	Q93VT6	4	Os04g0527800	4.00E-17	19	At_ Ch	Os_Ch 0.96	CJ586625	6.00E-16	M/S	
Hypothetical protein F24D7.19	none	*rs2|CT262|*	Q9CAC8	2	Os02g0833400	E-13	9	At_Ch	Os_Mi/Ch 0.80/0.79	CJ705089	2.00E-14	S	
OSJNBa0038O10.22	none	*fr1|gnl|UG|Ta_S17988349*	Q7XKI6	4	Os04g0527800	3.00E-34	18	At_Ch	Os_Ch 0.96	CJ590508	4.00E-38	S	

Hypothetical protein OSJNBa0054L14.15, DUF573 family protein	none	*rs1|CT48148|*	Q5QMU3	1	Os02g0771200	E-.064	4	unk	Os_Ch 0.68	CJ560872	8E-.08	M	
OSJNBa0095E20.4 CBS domain containing protein; predicted transcriptional regulator. CBS domains are found in otherwise unrelated proteins and enzymes however.	none	*fr1|gnl|UG|Ta_S16222188*	Q7XRN1	7	Os04g0136700	4.00E-72	48	unk	Ta_Ch 0.91	CD893659	6.00E-90	M	
Hypothetical protein Os06g0217700	none	*rs-2|CT12299|*	Q8LDD3	2	no hit to the peptides, but BLAST of the wheat EST gives Os06g0217700			unk	Os_Ch 0.90	AL810427	7.00E-04	M	
Hypothetical protein Os08g0254900	none	*rs3|CT3346|*	Q6UA21	4	Os08g0254900	1.00E-19	13	Os, At_Ch	Os_Ch	CJ555626	5.00E-12	S	
Hypothetical protein Os08g0254900 (Same gene as above but different peptides and different 2DE spots)	none	*Alb CT14839*	Q8LAE4	4	Os08g0254900	2.00E-19	21	Os, At_Ch	Os_Ch 0.95	no hits		S	
**Function is unknown or not verified;**													
**protein may be located in plastid and/or mitochondrion**													
Hypothetical protein At3g55760	none	*fr1|gnl|UG|Ta_S16256641*	Q8L722	4	Os protein ABA94451.	8.00E-22	7	unk	Os_Mi/Ch 0.71/0.52	CD928165	2.00E-31	M	
Hypothetical protein P0450E05.20	none	*rs2|CT7356|*	Q69IM6	3	Os09g0514600	5.00E-15	20	unk	Os_Mi/Ch 0.37/0.32	CK213747	3.00E-19	S	
RNA-bnding protein	none	*Alb CT9295*	Q7F2X8	2	Os01g0916600	8.00E-04	19	At_Mi	Os_Ch 0.51	CJ796423	4.00E-04	M/S	KCl
**Unknown cellular location and function; may or may not be plastid.**													
Serine/threonine protein kinase	2.7.11.1	*rs2|CT16233|*	Q69ML2	3	Os09g0237600	5.00E-32	16	unk	Os_Ot 0.87	BQ805107 (Butte 86)	3.00E-08	M	
Hypothetical protein F24I3.170	none	*rs3|CT6113|*	Q9M1J1	5	Os05g0383000	1.00E-47	48	unk	Os_Ot 0.86	CJ524270	1.00E-49	S	
Hypothetical protein At5g42960	none	*Alb CT9530*	Q8H0Y1	3	Zm gene LOC542205 DANA2	1.00E-11	13	no	Zm_Ot 0.73	CJ698918	5.00E-15	M	
Hypothetical protein OJ1007_H05.1	none	*Alb CT15399*	Q6L5C9	2	BLAST of peptides give no hits, but BLAST of wheat EST gives Os01g0621300	3.00E-63	unk	Os_Mi	Os_Ot 0.83	BE497136	1.00E-04	M	
Hypothetical protein OSJNBa0027L23.4	none	*rs3|CT1115|*	Q8W3H8	2	Os10g0576000 partial	4.00E-04	4	unk	unk	CJ790709 (Butte 86)	1.00E-06	M	
Hypothetical protein P0534A03.117	none	*rs1|CT15567|*	Q84ZC8	2	Os07g054880	7.00E-14	12	unk	Os_Ot 0.90	BQ838854	5.00E-18	M	
Hypothetical protein OSJNBa0068L06.10	none	*rs3|CT4817|*	Q7XTB3	1	No hits			unk	unk	CJ957672	0.017	M	
Unknown: was called Pore protein of 24 kD (OEP24)	none	*rs2|CT31951|*	Q75IQ4	9	Zm gene LOC542205 DANA2	2.00E-63	50	unk	Zm_Ot 0.76	BE419335	7.00E-64	M	
Unknown	none	*rs-2|CT5858|*	Q7XZF8	2	No hits		unk	unk	no	BE488570	3.00E-04	S	
Hypothetical protein At5g26990 Dif19-like	none	*rs3|CT32774|*	Q6NM26	1	Blast of wheat est gives		unk	unk	Os_Ot 0.56	CJ802195	4.00E-21	S	

**Cytoplasmic proteins, including large non/organellar complexes**													
**Carbohydrate**													
Phosphoglucomutase	5.4.2.2	*gi|18076790| Ta*	Q8VX48	10	Ta gene PGM	2.00E-34	30	Var_Ch/Cy	Ta_Ot 0.97			M/S	KCl
Phosphoglycerate mutase	5.4.2.1	*rs2|CT11033|*	Q7XYD2	2	Os01g0817700	2.00E-11	6	Var_Cy	Os_Ot 0.82	LOC543487	2.00E-11	S	KCl
Lactoylglutathione lyase	4.4.1.5	*rs2|CT303|*	O49818	9	Os05g0295800	8.00E-79	62	At_ Cy/Mi	Os_Ot 0.76	BQ579556	5.00E-94	S	
6-phosphogluconate dehydrogenase	1.1.1.44	*rs3|CT1036|*	O81237	4	Os06g0111500	1.00E-19	13	Var_Cy	Os_Sp 0.46	CJ9510844	2.00E-18	M/S	
**Cytoskeleton/division**													
Actin	none	*gi|3219769| St*	P93587	3	Zm cDNA AAB40102	2.00E-10	16	cytoskeleton	Zm_Ot 0.89	CD936404	2.00E-09	M/S	KCl
Kinetochore protein	none	*rs1|CT878|*	Q9M3X1	4	Ta gene LOC543011	3.00E-57	43	cytoskeleton	Ta_Ot 0.91			M	
Tubulin alpha chain	none	*rs2|CT16090|*	Q9ZRB7	2	As cDNA Q38771	9.00E-13	8	cytoskeleton	As_Ot 0.65	CJ678198	2.00E-11	S	KCl
**Protein Synthesis**													
Elongation factor 1-alpha	none	*rs1|CT1964|*	Q03033	4	Os sequence EAZ25787	6.00E-35	19	Var_Cy	Os_Ot 0.89	BJ266027	4.00E-39	M/S	KCl
Elongation factor 1-beta	none	*rs2|CT3406|*	P29546	2	Ta gene LOC543180	5.00E-13	13	Var_Cy	Ta-Ot 0.82	BQ805553 (Butte 86)	2.00E-13	S	KCl
60S acidic ribosomal protein P0	none	*rs2|CT5653|*	O24573	2	Os08g0130500	2.00E-04	10	Var_Cy	Os_Ot 0.59	CJ501310	1E-0.22	M	
60S ribosomal protein L12	none	*rs2|CT5492|*	Q6Z8E0	6	Os02g0699600	1.00E-55	51	Var_Cy	Os_Ot 0.98	BQ805624 (Butte 86)	2.00E-42	M	
40S ribosomal protein S13	none	*rs1|CT1786|*	Q05761	2	Ta gene LOC543174	3.00E-14	20	Var_Cy	Ta_Mi 0.61			M	
40S ribosomal protein S19	none	*rs1|CT2329|*	P40978	4	Os03g0424500	2.00E-18	28	Var_Cy	Os_Ot 0.94	CJ517180	1.00E-25	M	KCl
40S ribosomal protein S2	none	*rs3|CT1988|*	Q84M35	2	Os03g0807800	4.00E-13	9	unknown	Os_Ot 0.74	AL813301	1.00E-15	M	
Ribosomal protein S7 family protein	none	*rs1|CT3537|*	O24111	2	Os11g0482000	3.00E-14	14	cytoplasmic and organellar	Os_Ot 0.75	AL814121	1.00E-15	M	
40S ribosomal protein SA (p40)	none	*rs1|CT8806|*	Q8H3I3	2	Os07g0616600	6.00E-13	11	At_Cy	Os_Ot 0.87	AL813511	2.00E-12	M	
Arabidopsis Ribosomal protein L7Ae-like	none	*rs1|CT1795|*	Q8LBE4	4	Os03g0241200	3.00E-21	32	Os says nuclear but At says ribosomal	Os_Ot 0.82	CJ793263	1.00E-23	M	
**Regulatory Proteins**													
14-3-3-like protein B	none	*rs3|CT1688|*	Q43470	1	Os02g0580300	5.00E-07	6	Var_Cy	Os_Ot 0.86	LOC543463	9.00E-06	S	KCl
**Proteases/turnover**													
Leucyl aminopeptidase	none	*Alb CT17263*	Q6K669	8	Zm unigene AY103547; Os12g0434400	1.00E-13	22	Var_Cy	Os_Ot 0.92	CJ671816	9.20E-49	S	TRX
Proteasome, 20 S alpha 2 subunit	none	*rs1|CT428|*	Q6H852	8	Os02g0634900	8.00E-53	56	animal cyto	Os_Ot 0.75	BQ804160	2.00E-51	S	
Proteasome, 20S alpha 6 subunit	none	*rs1|CT1550|*	Q9FER4	3	Os03g0180400	8.00E-07	15	various? Uncertain?	Os_Mi 0.60	CJ786135	1.00E-08	S	KCl
Proteasome, 20S beta 3 subunit	none	*Alb CT16089*	Q93X33	2	Os06g0643100	6.00E-22	16	Os_Cy	Os_Sp 0.73	BQ167993	4.00E-23	S	
Proteasome, 20S beta 4 subunit	none	*rs1|CT12864|*	Q851D9	3	TA gene LOC543169	2.00E-30	24	animal cyto	Ta_Ot 0.52			S	KCl

Ubiquitin	none	*rs2|CT17460|*	Q40164	1	Os09g0483400	2.00E-08	12	no	Os_Ot 0.88	No hits		S	
**Mitochondria**													
**Carbohydrate metabolism**													
**Citric acid cycle**													
Succinyl-CoA ligase (GDP-forming), beta-chain	6.2.1.4	*rs3|CT7836|*	Q6K9N6	9	Os02g0621700	4.00E-69	33	Var_Mi	Os_Mi 0.94	CJ561187	4.00E-69	M/S	
Succinyl-CoA ligase, alpha subunit, putative	6.2.1.4	*rs3|CT16592|*	Q6ZL94	2	Os07g0577700	1.00E-05	9	Os _Mi	Os_Mi 0.88	1st peptide CJ776922; 2nd peptide BQ806605	0.00004; E-0.2	S	
Fumarate hydratase 2	4.2.1.2	*rs1|CT5252|*	Q9FI53	4	Os03g0337900	2.00E-27	11	Var_Cy/Mi	Os_Mi 0.84	CJ796073	3.00E-27	S	
**Malate**													
Malate dehydrogenase	1.1.1.37	*Alb CT18245*	Q94JA2	6	Os01g0649100	2.00E-36	20	Var_Gl/Mi	Os_Mi 0.83	CJ655783		M/S	KCl
**Sterols**													
**Misc**													
Carboxymethylenebutenolidase	3.1.1.45	*rs2|CT25422|*	Q8LQS5	3	Os01g0531500	2.00E-22	14	unk	Os_Mi 0.90	CJ794103	3.00E-50	S	
**Protein processing**													
FtsH protease		*CT5300*	O80983	2	Os01g0574500	1.00E-05	5	At_Mi	Os_Mi 0.78	CJ726153	0.029	M	
Peptidylprolyl isomerase	5.2.1.8	*rs1|CT2427|*	Q6XPZ6	3	Ta gene LOC542967	1.00E-26	20	Var_Ch/Cy/ Mi	Os_Mi 0.82			M/S	KCl TRX
**Energetics**													
H+-transporting two-sector ATPase, beta subunit	3.6.3.14	*rs3|CT9549|*	Q41534	20	Ta gene atp2	E-0	59	Var_Mi	Ta_Mi 0.83			M	
H+-transporting two-sector ATPase 24 kDa subunit, putative CF0	3.6.3.14	*rs3|CT9549|*	Q6IY71	10	Ta gene LOC543158	1.00E-106	68	At,Os,Ta_ Mi	Ta_Mi 0.93			M	
H+-transporting two-sector ATPase F0 ATP synthase, D chain, putative	3.6.3.14	*rs2|CT1500|*	Q7XXS0	10	Os08g0478200	2.00E-84	70	Var_Mi	Os_Ot 0.74	CJ796546	1.00E-95	M	
NADH dehydrogenase (ubiquinone)	1.6.5.3	*rs3|CT14413|*	Q9FLX7	5	Os03g0313000	2.00E-53	51	At, Os_Mi	Os_Mi 0.83	CD876944	4.00E-45	M/S	
Succinate dehydrogenase (ubiquinone) alpha subunit	1.3.5.1	*rs1|CT735|*	Q6ZDY8	4	Os07g0134800	3.00E-13	10	At, Os_ Mi	Os_Mi 0.83	CJ673678	4.00E-13	M/S	
**Membrane**													
Stomatin-like protein	none	*rs1|CT4875|*	Q7EZD2	7	Os08g0158500	3.00E-61	29	At_Mi	Os_Mi	BQ839331	4.00E-76	M	
Mitochondrial import inner membrane translocase	none	*rs1|CT2429|*	Q7XL02	4-Feb	Os04g0405100	3.00E-14	19	At,Os_Mi	Os_Mi 0.73	CV775750	3.00E-05	M	
Inner membrane protein (translocase)	none	*rs2|CT15095|*	Q9FWD5	1	Os03g0114900	3.00E-06	14	At, Os_ Mi	Os_Mi 0.48	no hits		M	
Mitochondrial import inner membrane translocase	none	*rs1|CT16664|*	Q8H7W5	1	Os03g0194500	5.00E-08	11	possible mito	Os_Ot 0.93	CJ497227	9.00E-14	M	
Outer mitochondrial membrane protein porin	none	*rs2|CT11435|*	P46274	6	Ta cDNA	6.00E-23	29	Var_Mi	Ta_Ot 0.84	CK208838	5.00E-27	M	
Porin-like protein	none	*rs2|CT16037|*	Q84P97	9	Os03g0202200	2.00E-72	49	mito or unknown	Os_Ot 0. 89	CJ730389	2.00E-68	M	
Processing peptidase alpha-chain	none	*fr2|gnl|UG|Ta_ S16057838*	Q9FNU9	5	Os01g0191500	3.00E-29	14	Var_Mi	Os_Mi 0.81	CK163397	4.00E-51	M	
Processing peptidase, beta subunit	none	*CT17686*	Q9AXQ2	4	Os03g0212700	7.00E-14	11	At, Os_Mi	Os_Mi 0.77	CK209510	9.00E-22	M	
Prohibitin	none	*Alb CT18142*	Q6AVQ4	5	Os07g0262200	1.00E-13	23	Os , Zm_ Mi	Os_Mi 0.58	BQ807199	2.00E-07	M	
Vesicle transport-related protein	none	*rs3|CT36099|*	Q851W1	1	Os03g0620800	0.009	2	Os_Mi	Os_Mi 0.67	No hits		M	
**Endomembrane**													

**Chaperones**													
Calnexin homolog	none	*Alb CT13783*	Q7XV86	6	Os04g0402100	2.00E-64	18	En	Os_Sp 0.97	CJ673676	3.00E-79	M	
Calreticulin	none	*rs2|CT10181|*	Q40041	16	Ta gene LOC606330	E-0.0	64	Os_Sp	Ta_Sp 0.99	CK208745	2.00E-108	M/S	
Coated vesicle membrane protein-like	none	*rs2|CT10991|*	Q6YZD2	5	Os08g0532900 and wheat gene ABB90548	2E-57 and 5E-55	38	Os_Sp	Os_Sp 0.98	CJ796357	2.00E-58	M	
Endoplasmin homolog	none	*rs3|CT5020|*	P36183	12	Hv gene LOC548247	4.00E-32	16	Os-Sp	Hv_Sp 0.95	CJ797429	7.00E-30	S	TRX
Lumenal binding protein cBiPe2	none	*rs2|CT16736|*	Q40058	15	Zm gene LOC732808	1.00E-119	33	Var_En	Zm_Sp 0.90	BQ838739 (Butte 86)	5.00E-33	M/S	TRX
Heat shock cognate 70 kDa protein 2	none	*gi|6969976|*	P22954	7	Os03g0276500	3.00E-30	19	Var_En	Os_Ot 0.87	CJ700388	4.00E-19	S	KCl
Peptidyl-prolyl cis- trans isomerase	5.2.1.8	*rs2|CT45|*	Q70YJ6	7	Hv cDNA CAD42633	2.00E-92	77	Var_En/Pl	Hv_Sp 0.98	CJ790157	5.00E-85	M	
Protein disulfide isomerase	5.3.4.1	*rs1|CT4544|*	Q9FE55	23	Ta genes PDI2 and PDI3	E-0	64	Var_En	Ta_Sp 0.99	BQ805352 (Butte 86)	7.00E-131	M/S	KCl TRX
**GTP-binding proteins**													
GTP-binding protein Rab6	none	*rs1|CT13289|*	Q8H4Q9	3	Os07g0496000	1.00E-10	19	Golgi face	Os_Ot 0.59	CV772236	2.00E-09	M	
Guanine nucleotide-binding protein, beta subunit	none	*rs3|CT8551|-*	P49027	12	Os01g0686800	8.00E-133	55	unk	Os_Ot 0.63	CK166764	1.00E-132	M/S	KCl
RAN GTPase activating protein 2	none	*fr1|gnl|UG|Ta_ S17973821*	Q9XEN1	5	Os05g0543200	2.00E-57	15	At nuclear envelope	Os_Ot 0.55	FGAS0043 92	1.00E-63	M	
Ras-related protein Rab11B or Ara2	none	*gi|3024502|*	Q40521	3	At gene Ara2	6.00E-16	16	Var_sp	At_Ot 0.79	Ta gene LOC54316 4	2.00E-14	M/S	
Ras-related protein Rab7	none	*rs3|CT763|*	Q40787	3	Os01g0227300	4.00E-20	17	other	Os_Mi 0.69	CJ791302	7.00E-20	S	
Ras-related protein RIC1	none	*rs1|CT10949|*	Q68V18	5	Os01g0558600	2.00E-26	36	Var_Go/Sp	Os_Sp 0.85	CJ515929	2.00E-26	M	
Ras-related protein RIC2	none	*rs3|CT8694|-*	Q9ATK6	7	Os06g0551400	2.00E-64	43	unk	Os_Ot 0.68	CJ522000	3.00E-66	M	
Ras-related protein RGP2	none	*rs2|CT15721|-*	Q40723	2	Os05g0280200	1.00E-06	13	plasma membrane?	Os_Ot 0.73	CJ797651	E-0.002	S	
Ras-related protein YPT3	none	*gi|267527|*	Q01111	2	Sl gene Rab11a	6.00E-15	13	sp	Sl_Ot 0.88	Ta gene LOC54316 4	3.00E-09	M	
**Proteases/turnover**													
Cathepsin B-like cysteine proteinase	3.4.22.1	*rs1|CT2860|*	Q03106	2	Ta gene LOC543298	2.00E-27	11	endo?	Ta_Sp 0.94			S	
**Lipid metabolism**													
Ent-kaurene oxidase	1.14.13.78	*rs3|CT45456|*	Q673G1	2	Hv cDNA AAT49064, no N-term; Os06g0569500	0.0000005; 0.000004	5	At_Ch,outer membrane Os_Sp	Os_Sp 0.94	CD921882	7.00E-08	M	
**Electron transport**													
Putative cytochrome P-450LXXIA1 (Cyp71A1) family	none	*gi|13661774|*	Q8W0B2	2	Os01g0627500	4.00E-15	5	Sorghum microsome	Os_Sp 0.83	cDNA in NCBI nr AAQ13549	9.00E-14	M	
**Transport**													

Translocon-associated protein, alpha subunit	none	*rs-2|CT3909|*	P45434	2	Os06g0715500	7.00E-14	9	Os_en	Os_Sp 0.98	CJ547227	2.00E-15	M	
**Storage proteins**													
Alpha/beta-gliadin	none	*rs2|CT743|*	P02863	5	Ta gene LOC543192 is missing N-terminal peptides	1.00E-22	unk	Ta_En	MISSING n-TERM			M	
Alpha-amylase inhibitor 0.19	none	*rs3|CT15368|*	P01085	5	Ta gene LOC780684	3.00E-35	21	Ta_En	Ta_Mi/Ot 0.33/0.31			M/S	
Alpha-amylase/trypsin inhibitor CM16	none	*rs2|CT4421|*	P16159	7	Ta gene LOC543286	3.00E-51	15	Ta_En	Ta_Sp 0.99			M/S	
Alpha-amylase/trypsin inhibitor CM2	none	*rs3|CT4349|*	P16851	4	Ta gene LOC543282 ? Problem? Emailed NCBI	8.00E-32	50	Ta_En	Ta_Sp 0.98			M/S	
Alpha-amylase/trypsin inhibitor CM3	none	*rs2|CT14882|*	P17314	8	Ta gene LOC543281	1.00E-94	73	Ta_En	Ta_Sp 0.99			M/S	
Chymotrypsin inhibitor WCI	none	*rs3|CT15100|*	P83207	2	Ta gene WCI N-terminus is missing	4.00E-18	25	Ta_En	Missing N-term			M	
Gamma 3 hordein-like	none	*fr3|gnl|UG|Ta_S16238145*	Q6EEY6	9	Hc gene AAQ63842	1.00E-51	40	Hv_En	Hc_Sp 0.53	CD909761	4.00E-121	M	
Gamma-gliadin	none	*rs2|CT1907|*	Q94G97	5	Ta genomic DNA P06659	1.00E-36	45	Ta_En	Ta_Sp 0.79			M	
Puroindoline-B	none	*rs3|CT727|*	Q10464	7	Ta gene LOC543301	6.00E-67	65	Ta_En	Ta_Sp 0.91			M	
Purothionin A-I	none	*rs-1|CT11520|*	P01543	3	Ta gene thionin	5.00E-25	27	Ta_En	Ta_Sp 0.92			M	
Storage protein	none	*rs3|CT4813|*	Q38794	3	Ta gene LOC542891	7.00E-17	25	Ta_En	Ta_Sp 0.97			M/S	
**Vacuole**													
vacuolar proton-ATPase subunit A	none	*rs2|CT16599|*	Q9FS11	13	Ta gene LOC780589	1.00E-96	37	cytoplasmic side of vacuole membrane, endo-membranes	Ta_Ot 0.96	CJ701868	1.00E-52	M/S	
Phytepsin	none	*rs2|CT15572|*	P42210	18	Ta gene WAP1	3.00E-111	46	Hv vacuole	Ta_Sp 0.91			M	
**Peroxisome**													
Catalase.	1.11.1.6	*rs1|CT15658|*	P55313	2	Ta gene LOC542902	2.00E-05	3	Ta_Pe	Ta_Ot 0.63			S	KCl
**Nucleus**													
Putative fibrillarin	none	*rs3|CT11730|*	Q6K701	3	At gene FIB1	5.00E-29	15	At gene FIB1	5.00E-29	BQ804990 (Butte 86)	3.00E-19	M	

Histone H2A.1	none	*Alb CT1281*	P02275	2	Blast of peptides gives no hits; blast of wheat est gives Ta gene WHTHIH2A	None; E-0	unk	Blast of peptides gives no hits; blast of wheat est gives Ta gene WHTHIH2A	None; E-0	BQ804577 (Butte 86)	9.00E-28	S	
Histone acetyltransferase	2.3.1.48	*Alb CT16377*	Q9M4U5	9	Ta gene LOC780626	5.00E-54	42	Ta gene LOC780626	5.00E-54	CJ902202	2.00E-55	M	
Nucleic acid binding protein	none	*rs-2|CT23202|*	Q68Q07	1	Os04g0620700	3.00E-04	3	Os04g0620700	3.00E-04	CJ515598	3.00E-14	M	
Nucleosome/chromatin assembly factor	none	*rs3|CT42756|*	Q8L8G4	1	Os01g0710000	9.00E-07	3	Os01g0710000	9.00E-07	CJ782194	4.00E-07	S	
HMG1/2-like protein	none	*rs3|CT1932|*	P40621	2	Ta gene Hmgb3	3.00E-14	19	Ta gene Hmgb3	3.00E-14	BJ308279	2.00E-17	M	
**Unknown. May be cytoplasmic, ER, glyoxysomal, peroxisomal or mitochondrial; probably not plastid**													
Alanine transaminase	2.6.1.2	*rs2|CT961*|-	P52894	1	Os10g0390500	8.00E-05	3	Var_Cy/Gl/Mi/Pe	Os_Ot 0.65	CJ524048	E-.002	S	KCl
Calmodulin-binding protein MPCBP	none	*rs-1|CT20236|*	Q69SQ6	1	Blast of peptides gives no hits, but blast of wheat EST gives Zm gene AF250191	4.00E-29	unk	unk	Zm_Ot 0.52	CJ551308	2.00E-08	S	
Cytochrome B5, putative	none	*rs1|CT4994|*	Q94DH6	5	Os01g0971500	9.00E-53	51	Var_En/Mi	Os_Ot 0.91	BQ806923 (Butte 86)	9.00E-57	M	
DNAJ-like protein	none	*rs2|CT1298|*	Q84PD0	2	Ta gene J3 J-domain protein	7.00E-07	5	Os_Mi	Ta_Ot 0.82	CJ495906	2.00E-10	M	
Heat shock protein STI (Stress inducible protein) (GmSTI)	none	*rs1|CT205|*	Q6H660	1	Os02g0644100	7.00E-09	3	unk	Os_Ot 0.63	CV066378	3.00E-09	S	KCl
Reversibly glycosylated polypeptide	none	*rs3|CT4124|*	Q9ZR33	8	Ta gene RGP	3.00E-57	36	Ps_Go	Ta_Ot 0.76	CJ786802	5.00E-59	M/S	KCl
Ricin B-related lectin domain containing protein	none	*rs3|CT2852|*	Q9FTY4	1	Os01g0104400	2.00E-05	5	unk	Os_Ot 0.91	CJ817322	3.00E-05	M	
Ricin B lectin domain containing protein”	none	*rs3|CT13269|*	Q6Z4N6	12	Os07g0683900, also wheat gene with lower score	1.00E-65	57	unk	Os_Ot 0.62	CJ681617	1.00E-103	M/S	
TGB12K interacting protein 3 (TIP3)	none	*Alb CT17373*	Q40785	4	Os03g0851700	3.00E-14	24	probl cytoplasmic	Os_Ot 0.70	BQ806470 (Butte 86)	1.00E-18	M	

[a]Enzyme name indicated as preferred in the BRENDA database [100]; other protein names based on consensus in latest literature.

[b]Enzyme EC number from the BRENDA database [100].

[c]Identifier for sequence that was the best fit to the MS/MS used for [6]. Sequences beginning gi are from the NCBI nonredundant database [34], and are followed by initials for the Genus and speciesi. All contig data are for wheat. To retrieve the contig do as follows. Contigs prefixed by Alb are from wEST and can be obtained at: [http://wheat.pw.usda.gov/cgi-bin/westsql/contig.cgi?id=5:<CONTIG_NUMBER>]. Replace <Contig Number> including brackets with the contig number [35]. Contigs precided by rs are from HarvEST. To obtain them it is necessary to download the database archives from [http://harvest.ucr.edu/] [36]. Contigs preceded by fg are from NCBI. To obtain them it is necessary to download the NCBI *Triticum aestivum* unigene build [37] and search using the contig number beginning TA. To view the original tandem MS/MS results, download the xtml file from [http://wheat.pw.usda.gov/pubs/] under the directory 2008/Dupont and search the xtml file for the protein of interest, using the contig or gi number. An xtml viewer is available from the GPM website [http://h.thegpm.org/tandem/thegpm_upview.html].

[d]The Swiss Protein number [102] given in [6]. This number is used to correlate data in this paper with the set of peptides in the Supplementary Table of [6]. In some cases it differs from the current assigned identification for that set of peptides.

assigned identification for that set of peptides.

[e]Number of nonredundant peptides detected.

[f]Most closely related gene or cDNA found by a BLAST search of the peptides against the NCBI nr database. All accessions beginning Os are rice genes.

[g]Score for BLAST results.

[h]Percent coverage of the complete protein sequence encoded by the closest NCBI nr accession [34].

iCellular location based on annotations of the NCBI sequences, information in the BRENDA database, and in the literature. Ae tau, Aegilops tauschii; As, Avena sativa; At, Arabidopsis thaliana; Hc, Hordeum chilense; Hco, Hordeum comosum; Hv, Hordeum vulgare; Lp, Lolium perenne; Mp, Marchantia polymorpha; Mt, Medicago truncatula; Nt, Nicotiana tabacum; Os, Oryza sativa; Pl, Phaseolus lunatus Ps, Pisum sativum; Rc, Ricinus communis; Sb, Sorghum bicolor; Sc, Secale cereale; Sl, Solanum lycopersicum; So, Spinacia oleracea; St, Solanum tuberosum; Ta, Triticum aestivum; Vf, Vicia faba; Vu, Vigna unguiculata Zm, Zea mays; Var indicates various species. Ch, chloroplast; Cy, cytoplasm; En, endomembrane; Gl, glyoxysomes; Nu, nucleus; Pe, peroxisomes; Pl, plastid; Sp, secretory protein; Mi, mitochondria; Ot, other.

[j]Signal peptide prediction based on the first 200 amino acids of the protein encoded by the most closely related gene or cDNA sequence with a full N-terminus. In almost all cases it is the gene or cDNA indicated in the column "closest NCBI accession". Abbreviations as in i, above. Target P [41] scores range from 0 to 1.0, where 1.0 is the highest probability.

[k]Example chosen from the highest scoring ESTs found by a BLAST [40] search of the peptides against the NCBI wheat EST database. If the EST is from Butte 86, the wheat variety used for this study, that is also indicated.

[l]Protein was found in the membrane (M), soluble (S) or membrane and soluble (M/S) fractions [6].

[m]KCl indicates that the protein or a homolog was also identified in the KCl-extract of [42]. TRX indicates that the protein was identified as thioredoxin binding in [31].
