# Supplementary material for: Metabolic pathways of the wheat (Triticum aestivum) endosperm amyloplast revealed by proteomics
Source: BMC Plant Biol. 2008 Apr 17;8:39. doi: 10.1186/1471-2229-8-39 (PMC2383896; doi:10.1186/1471-2229-8-39)
Supplement: Additional file 2 — Figures 2-17. [file 1471-2229-8-39-S2.zip › fig 1 with links forfinal revised version/Fig2.pdf]

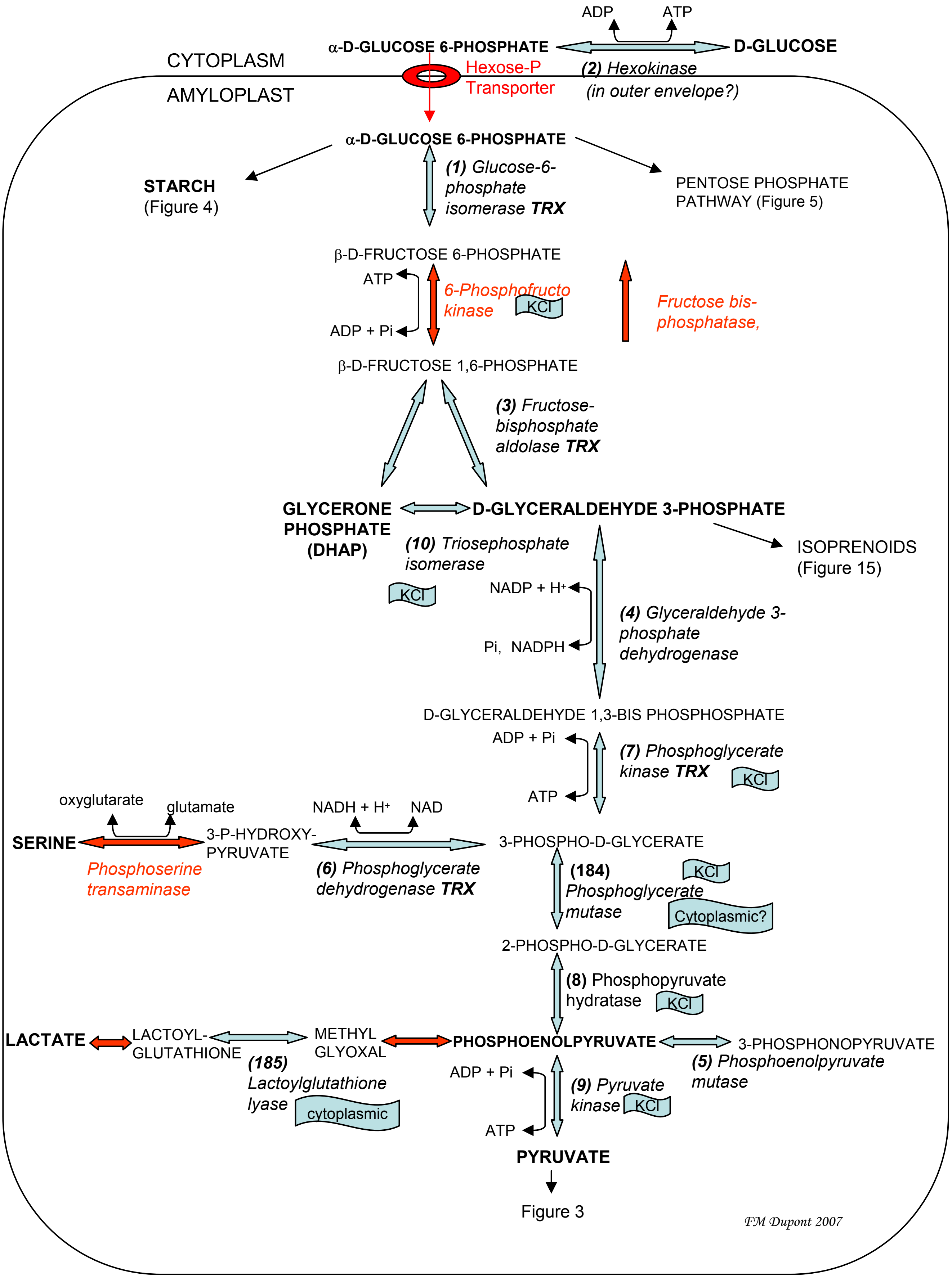

Figure 2. Glucose metabolism and glycolysis. Details of symbols and meaning of colors for all figures are given in Fig. 3. Hexokinase may be in the outer envelope, or within the plastid.
