# Supplementary material for: Metabolic pathways of the wheat (Triticum aestivum) endosperm amyloplast revealed by proteomics
Source: BMC Plant Biol. 2008 Apr 17;8:39. doi: 10.1186/1471-2229-8-39 (PMC2383896; doi:10.1186/1471-2229-8-39)
Supplement: Additional file 2 — Figures 2-17. [file 1471-2229-8-39-S2.zip › fig 1 with links forfinal revised version/Fig3.pdf]

**Pyruvate Dehydrogenase**

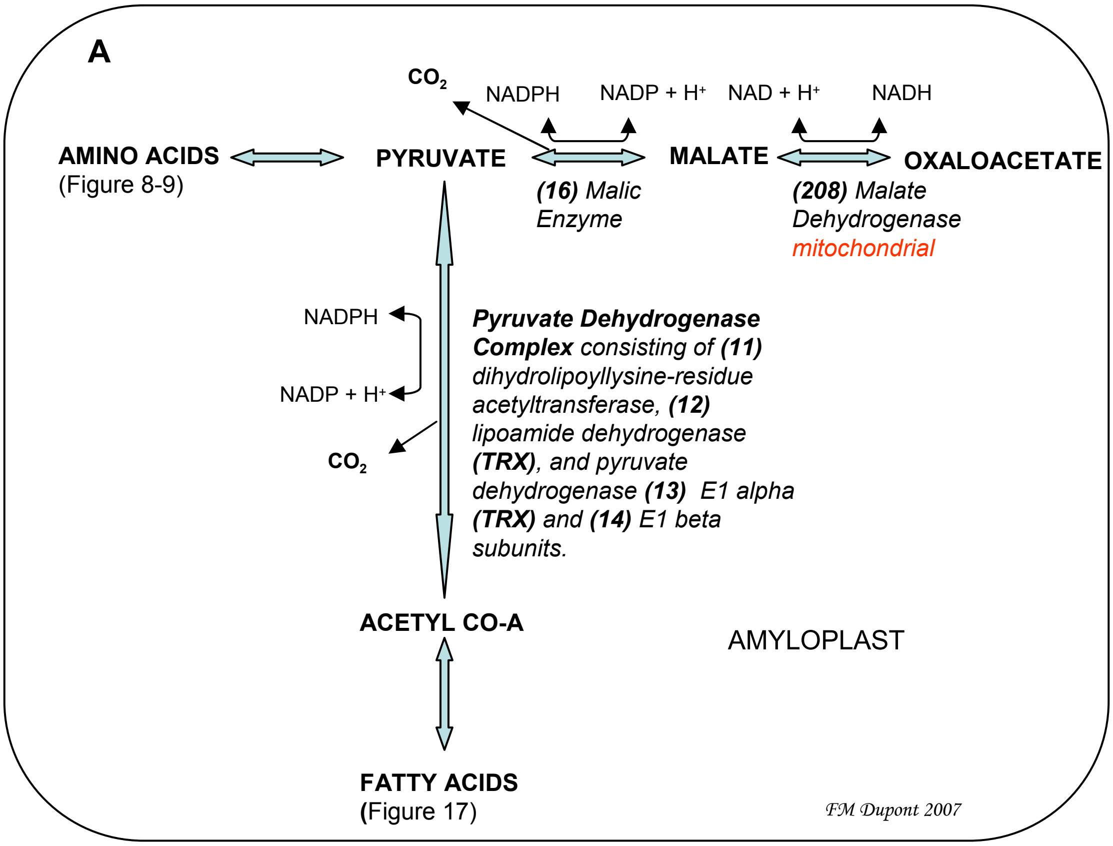


**Citric Acid Cycle**

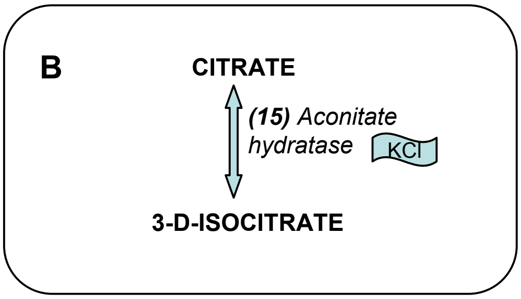


**C. Legend for All Figures**

**Black font** indicates that enzyme was detected in the amyloplast preparation, regardless of cellular location.

**Red font** indicates that enzyme was not detected in the amyloplast preparation.

KCl indicates that enzyme was previously detected in the KCl extract

Cytoplasmic indicates that enzyme is thought to be cytoplasmic.

**TRX** indicates that enzyme was identified as a thioredoxin target.

**Figure 3 . A. Pyruvate dehydrogenase. B. Citric acid cycle components C. Legend for all figures.**
