# Supplementary material for: Metabolic pathways of the wheat (Triticum aestivum) endosperm amyloplast revealed by proteomics
Source: BMC Plant Biol. 2008 Apr 17;8:39. doi: 10.1186/1471-2229-8-39 (PMC2383896; doi:10.1186/1471-2229-8-39)
Supplement: Additional file 2 — Figures 2-17. [file 1471-2229-8-39-S2.zip › fig 1 with links forfinal revised version/Figure1/s0001.htm]

Slide 1


**(****17-24****)** Starch synthesis

ADP-GLUCOSE

ADP-GLUCOSE

**(****134****)** ADPG-

transporter

D-GLUCOSE
6-PHOSPHATE

GLUCOSE-6-PHOSPHATE

**(****19-21****)**

*Hexose-P**transporters*

D-GLUCOSE

*Glucose**transporter*

D-GLUCOSE

**(136-142)** PLASTID RIBOSOMES

**(149-163)**

Protein cleavage
and folding

Proteolysis

Protein
synthesis on cytoplamic ribosomes

PLASTID
ENZYMES

**(143-148)**
Protein import complex

**(135)** Amino
Acid Selective Chanel

RIBULOSE
5-PHOSPHATE

**(****25-30****)** Pentose phosphate pathway

Other Sugars

Photorespiration

**PHENYLALANINE,****TRYPTOPHAN,** **TYROSINE**

**(****33-39****)** Aromatic amino acids

**(****83-86****)** Pyrimidine synthesis

**URIDINE-****5�PHOSPHATE**

**ATP**

**(****78-82****)** Purine synthesis

**ASPARTATE**

**NAD**

**(****82****)**

**HISTIDINE**

**(****70-74****)** Histidine

**GLUTAMINE**

FGAM

b-D-FRUCTOSE 6-PHOSPHATE

**(****3-10****)** Glycolysis

**PYRUVATE**

ACETYL CO-A

**FATTY ACIDS**

**(****11-14****)** Pyruvate dehydrogenase

**(****105-112****)** Fatty acid biosynthesis

**CAROTENOIDS**

GLYCERALDEHYDE
3-P

**(****95-97****;** **97-104****)** Isoprenoid synthesis

**ISOLEUCINE,****LEUCINE, VALINE**

**(****48-55****)** Branched chain amino
acids

**(****56-62****)** **CYSTEINE**

and other sulfur metabolism

**ALANINE,** **LYSINE AND** **THREONINE**

**(****40-47****)** Aspartate
family

**GLUTAMATE**

ORNITHINE, ARGININE

**(****63-69****)** Glutamate
family

**GLYCINE**

FORMATE

10, FORMYL TETRAHYDROFOLATE

**SERINE**

**TETRAHYDROFOLATE**

Used in
purine synthesis

**(****31-32****)** 1-CARBON METABOLISM

**(130-131)** Signaling

**(113-115)** Ferredoxin-thioredoxin
system for generation of NADPH

CITRATE

ISOCITRATE

**(****15****)**

**NADPH**

**NADPH**

**ATP**

**Thiamine,**

**Vitamins** **B1 & B2**

tRNA-Glu to biliverdin
to **PHYTOCHROME**

**(116-121)** Free
radical scavengers

**(****68****)**

**(****87-94****)** Porphyrin synthesis

**Vitamins** **E & K**

**(132-133)** ABC-type transporter

**PHOSPHOENOLPYRUVATE**

**(****9****)**

**(****1****)**

3P-GLYCERATE

**(****6****)**

**SERINE**

**SERINE**

**(****2****)**

MALATE

**(****16****)**

**(****183****)**

D-GLUCOSE-1-PHOSPHATE

GLUCOSE-1-PHOSPATE

**(****183****)**

5, 10,
METHYLENE THF

*FM Dupont 2007*

**CYTOPLASM**

**STARCH** **GRANULE**

**CYTOPLASM**

**AMYLOPLAST**
