# Supplementary material for: Metabolic pathways of the wheat (Triticum aestivum) endosperm amyloplast revealed by proteomics
Source: BMC Plant Biol. 2008 Apr 17;8:39. doi: 10.1186/1471-2229-8-39 (PMC2383896; doi:10.1186/1471-2229-8-39)
Supplement: Additional file 2 — Figures 2-17. [file 1471-2229-8-39-S2.zip › fig 1 with links forfinal revised version/Figure1/v3dcmnt.htm]

Slide 1
