# Supplementary figures and images for: Metabolic pathways of the wheat (Triticum aestivum) endosperm amyloplast revealed by proteomics
Source: BMC Plant Biol. 2008 Apr 17;8:39. doi: 10.1186/1471-2229-8-39 (PMC2383896; doi:10.1186/1471-2229-8-39)

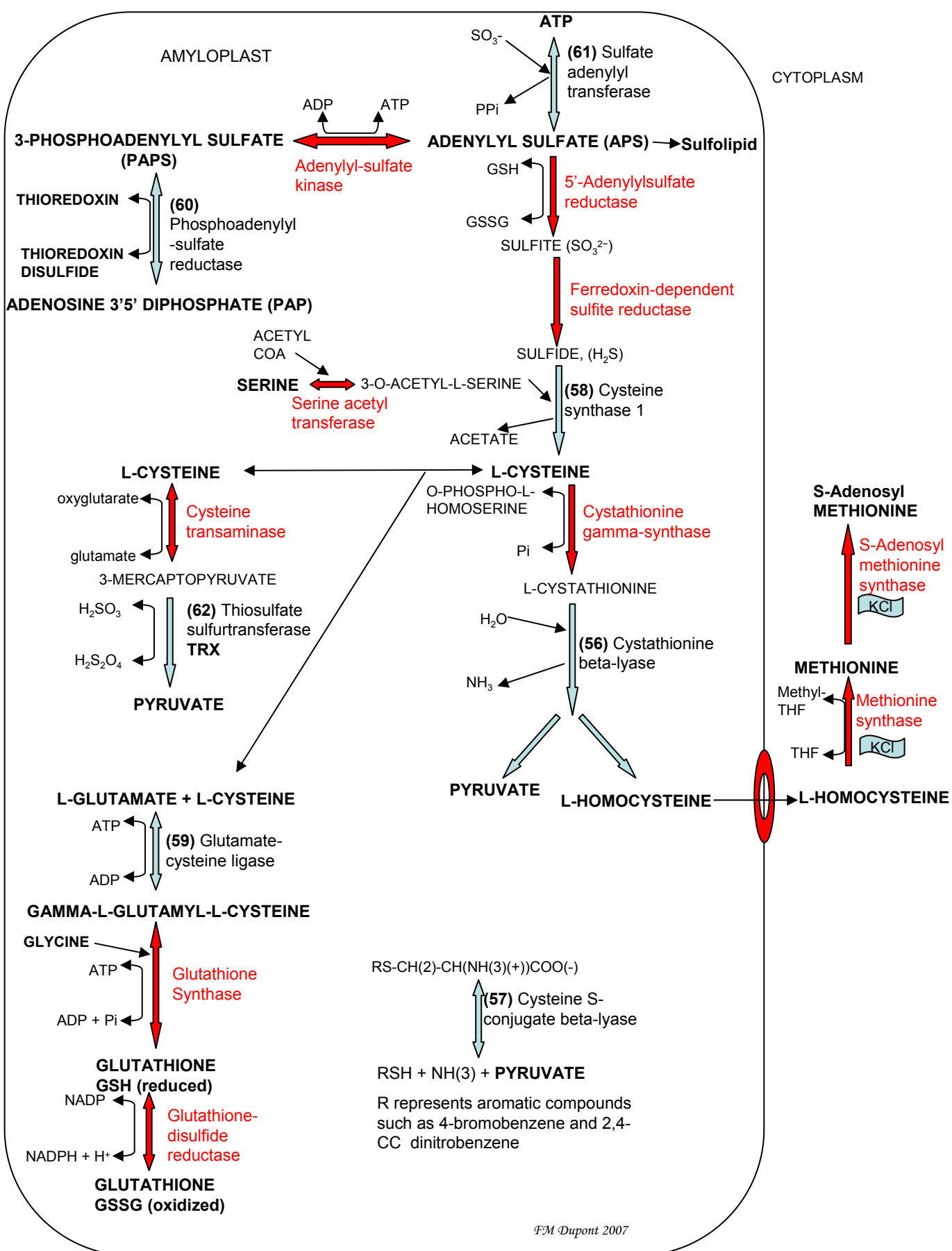

**Figure 10. Cysteine and sulfur metabolism.**

Supplement: Additional file 2 — Figures 2-17. [file 1471-2229-8-39-S2.zip › fig 1 with links forfinal revised version/Fig10.pdf]

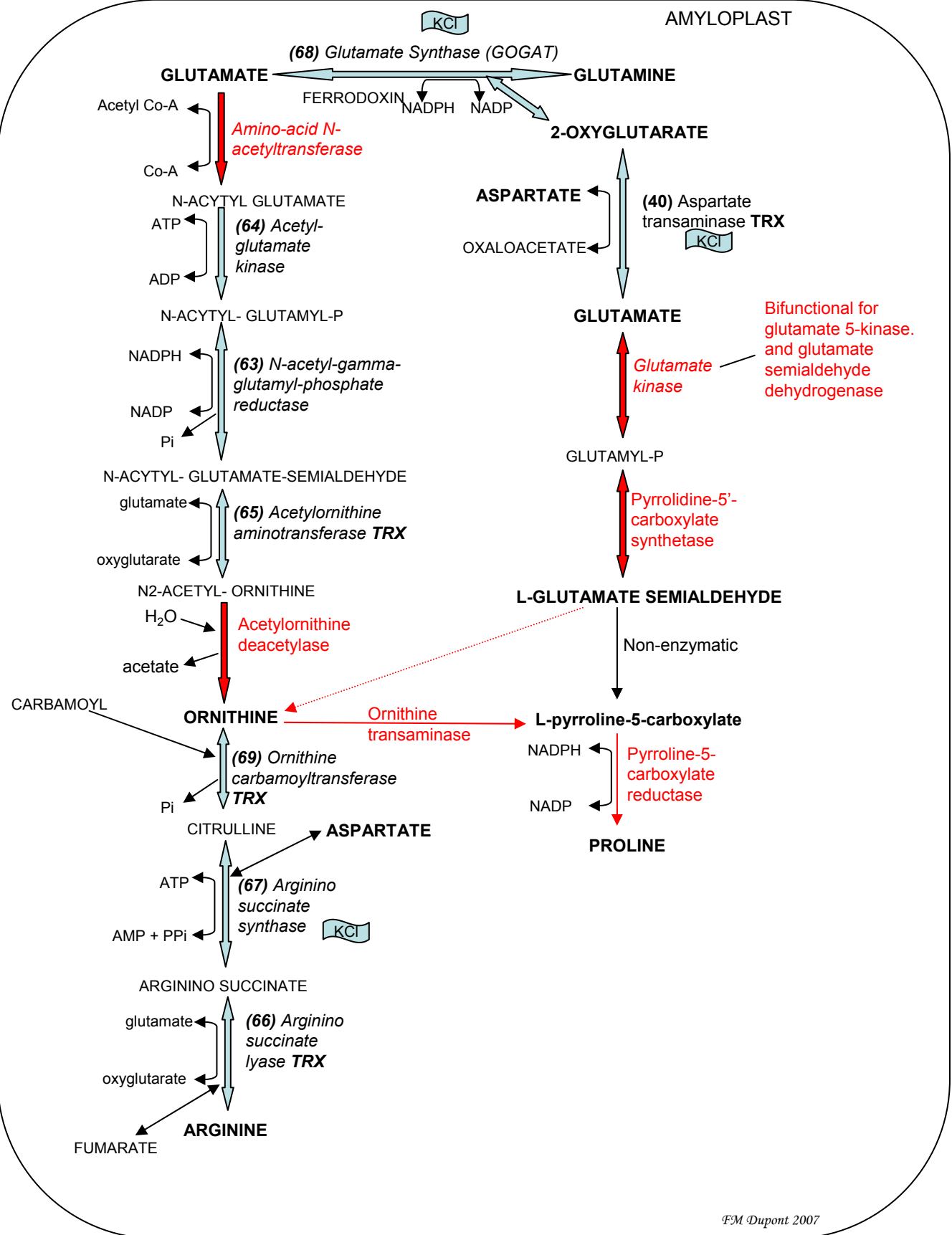

**Figure 11. Glutamate family of amino acids.**

Supplement: Additional file 2 — Figures 2-17. [file 1471-2229-8-39-S2.zip › fig 1 with links forfinal revised version/Fig11.pdf]

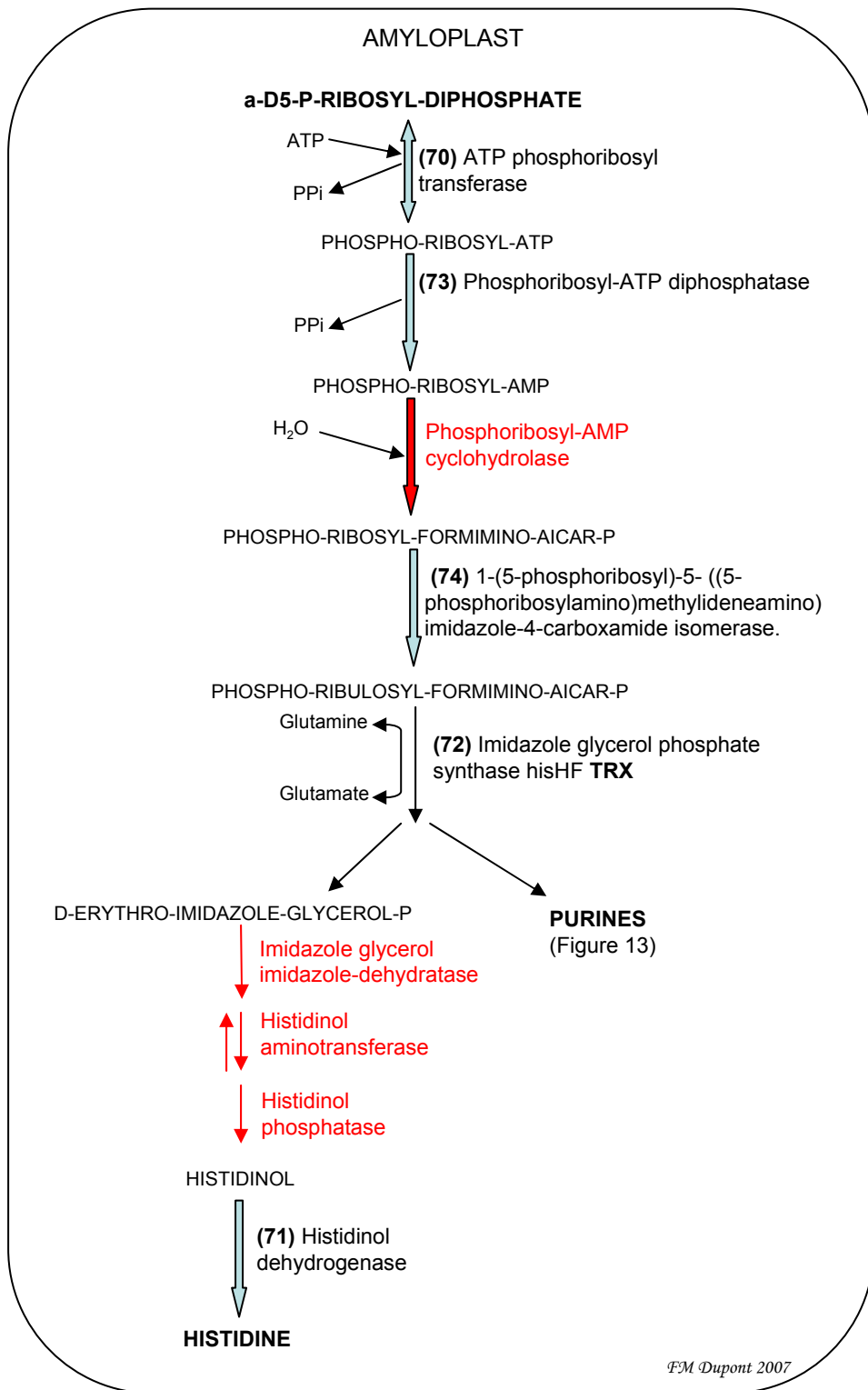

**Figure 12. Histidine synthesis.**

Supplement: Additional file 2 — Figures 2-17. [file 1471-2229-8-39-S2.zip › fig 1 with links forfinal revised version/Fig12.pdf]

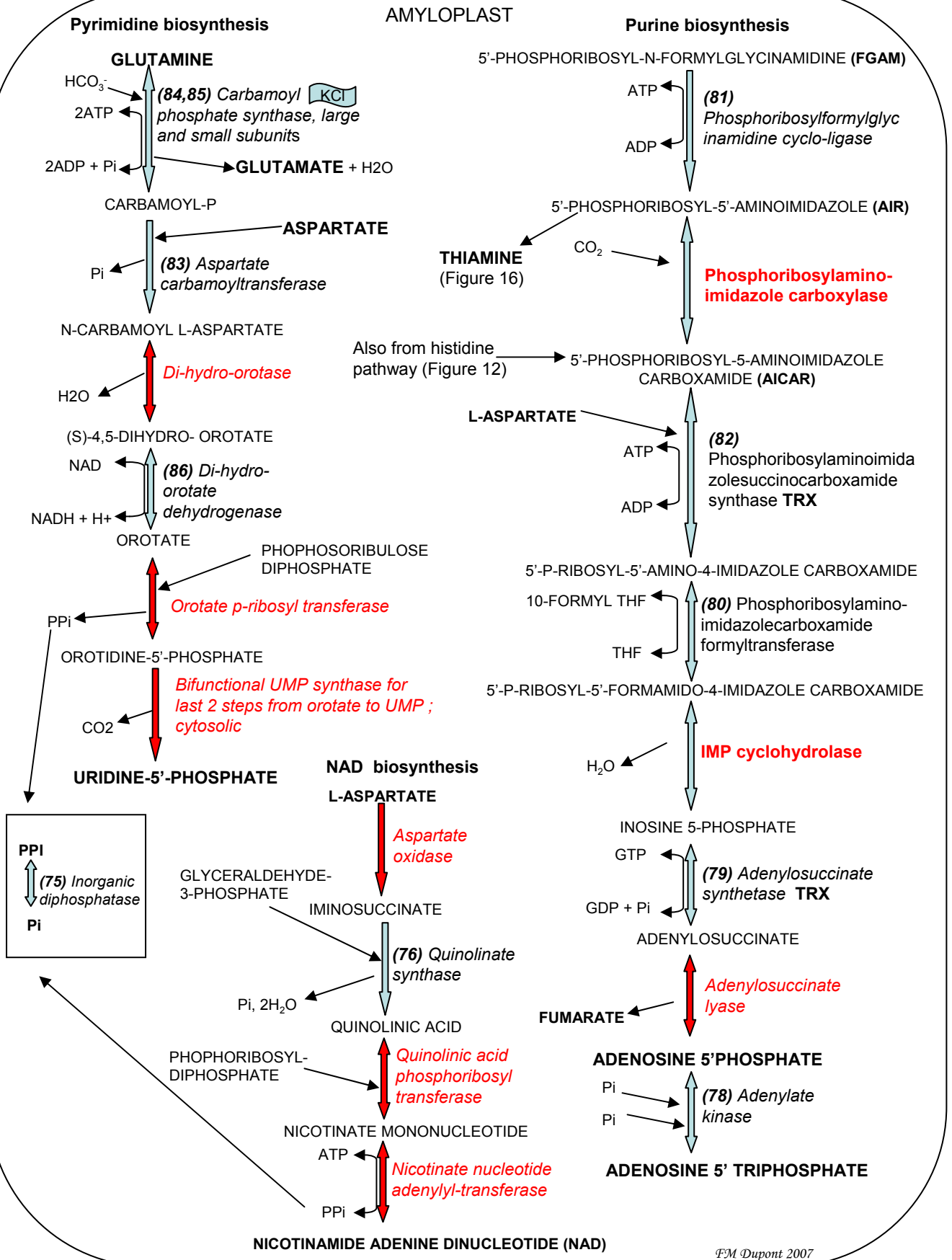

**Figure 13. Nucleic acid synthesis.**

Supplement: Additional file 2 — Figures 2-17. [file 1471-2229-8-39-S2.zip › fig 1 with links forfinal revised version/Fig13.pdf]

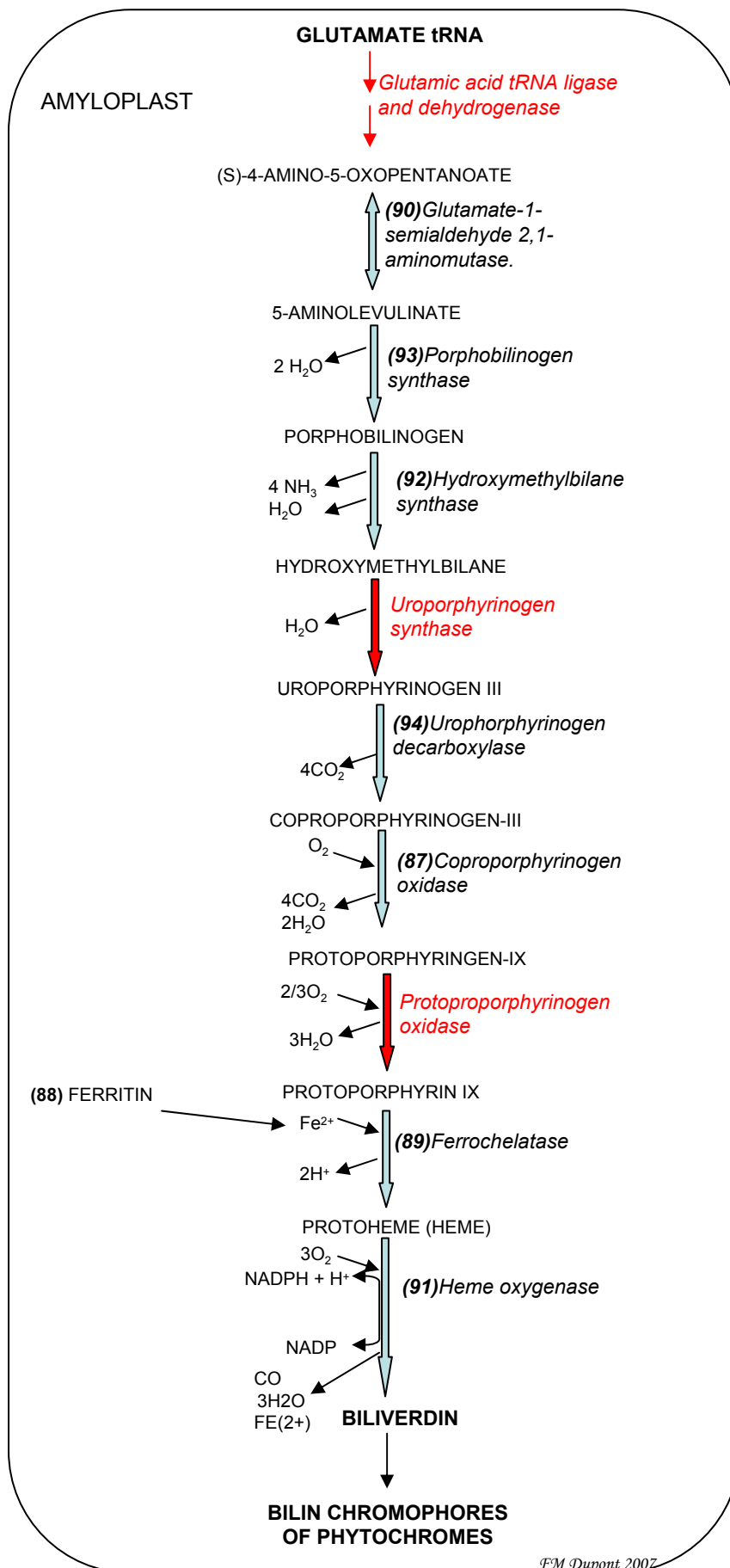

FM Dupont 2007

**Figure 14. Porphyrin synthesis.**

Supplement: Additional file 2 — Figures 2-17. [file 1471-2229-8-39-S2.zip › fig 1 with links forfinal revised version/Fig14.pdf]

## Isoprenoid Biosynthesis II

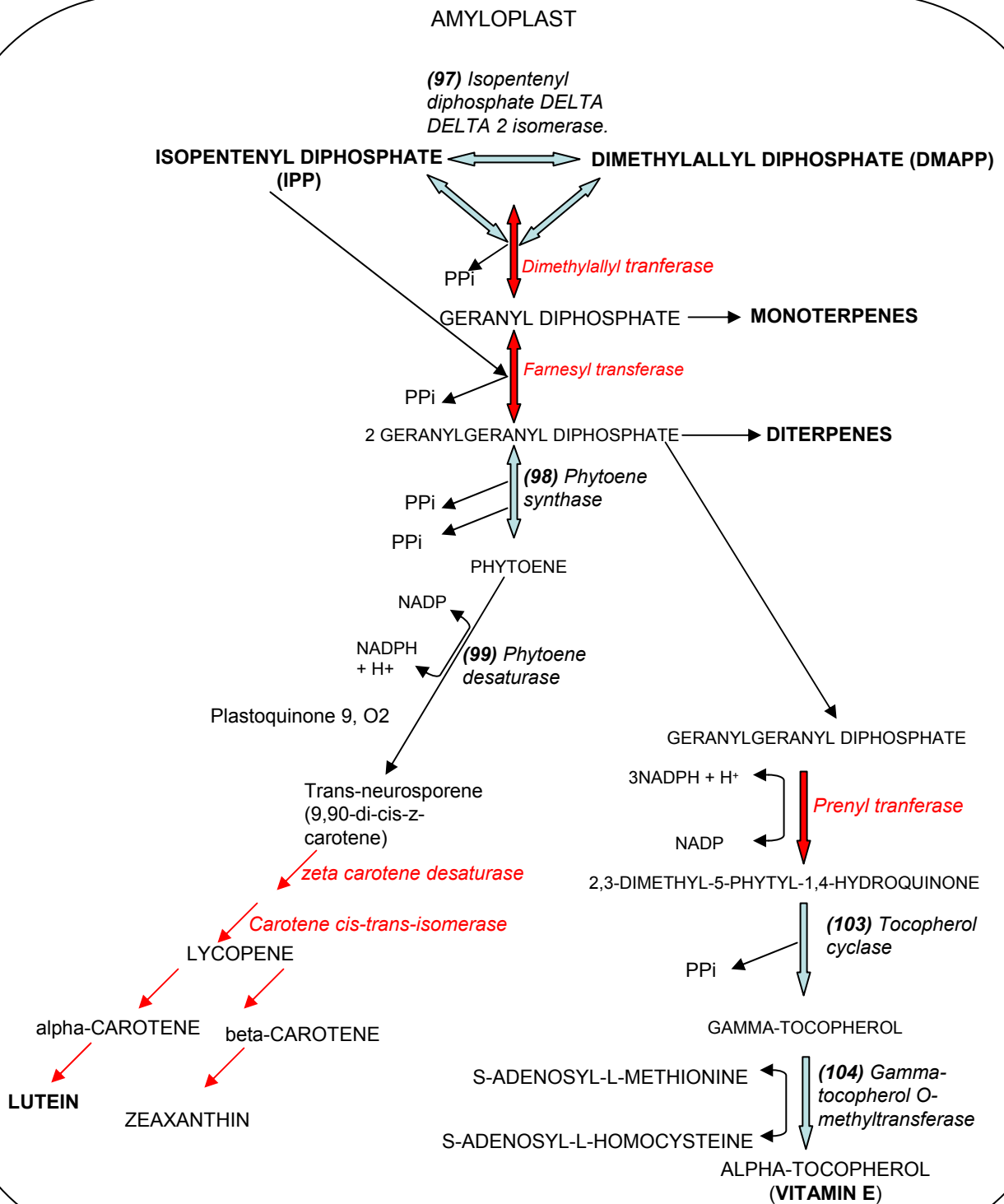

FM Dupont 2007

**Figure 15b. Isoprenoid synthesis II.**

Supplement: Additional file 2 — Figures 2-17. [file 1471-2229-8-39-S2.zip › fig 1 with links forfinal revised version/Fig15b.pdf]

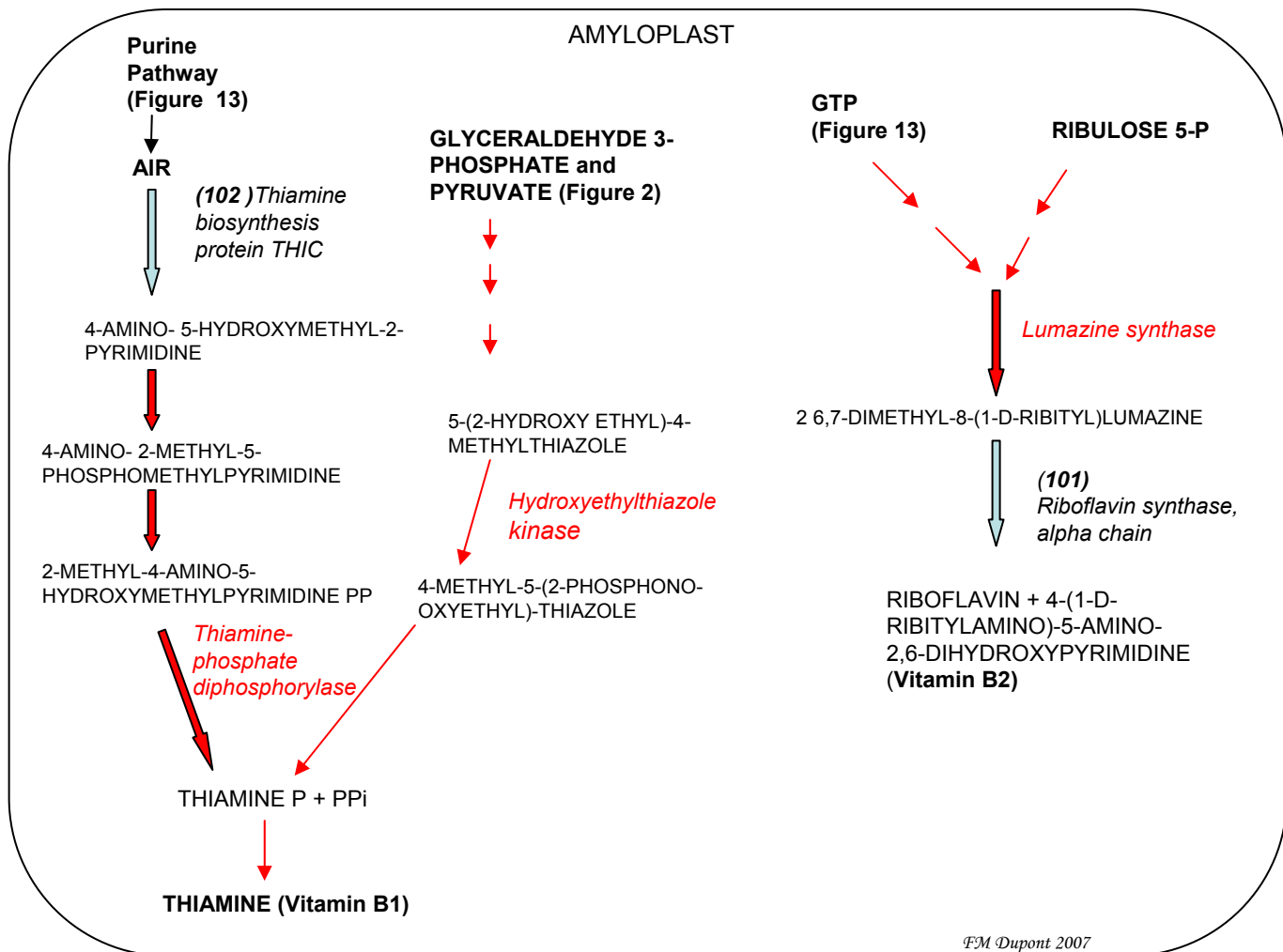

**Figure 16. Vitamin and cofactor synthesis, nucleic acid-related pathways.**

Supplement: Additional file 2 — Figures 2-17. [file 1471-2229-8-39-S2.zip › fig 1 with links forfinal revised version/Fig16.pdf]

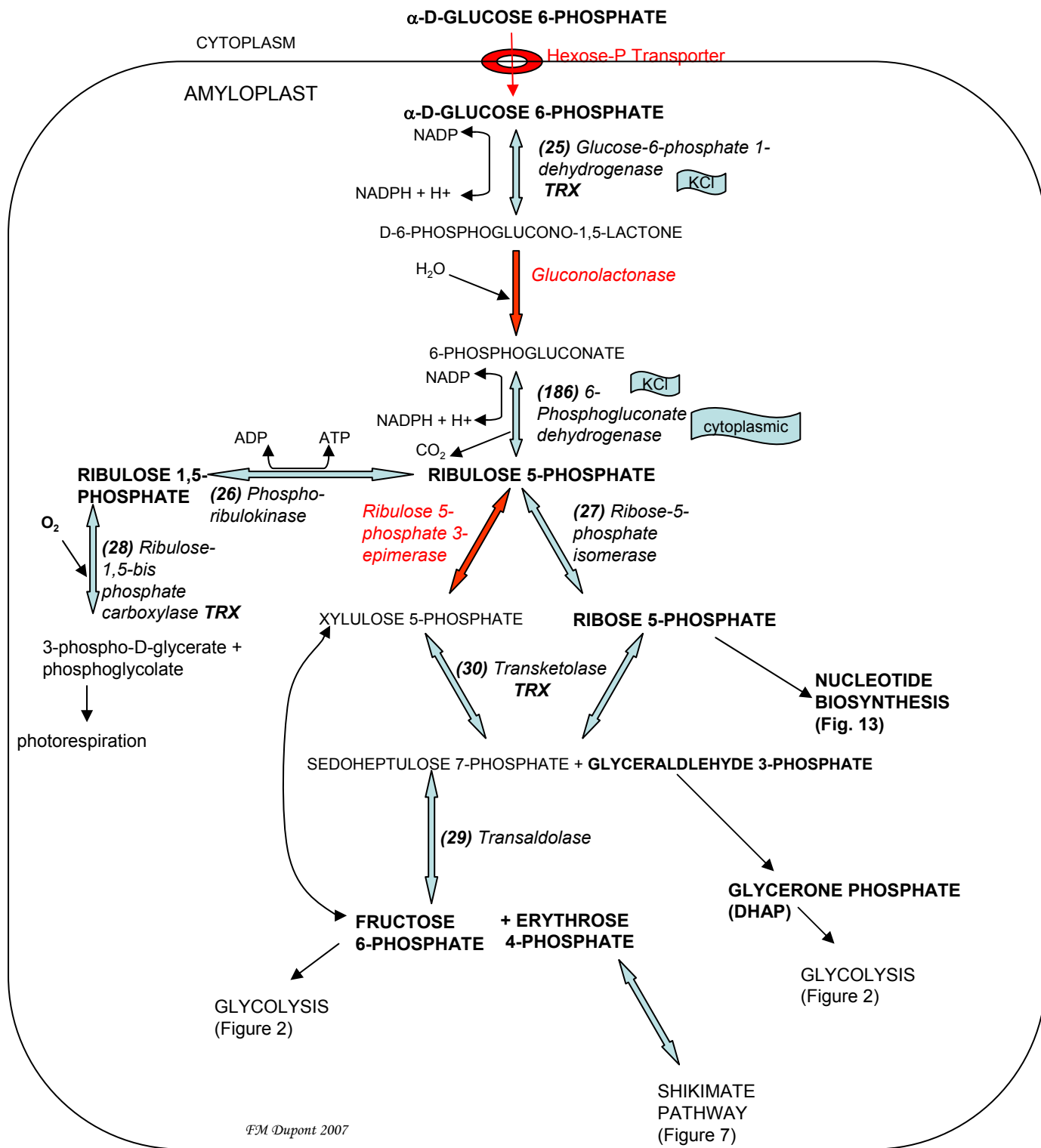

**Figure 5. Pentose phosphate pathway.**

Supplement: Additional file 2 — Figures 2-17. [file 1471-2229-8-39-S2.zip › fig 1 with links forfinal revised version/Fig5.pdf]

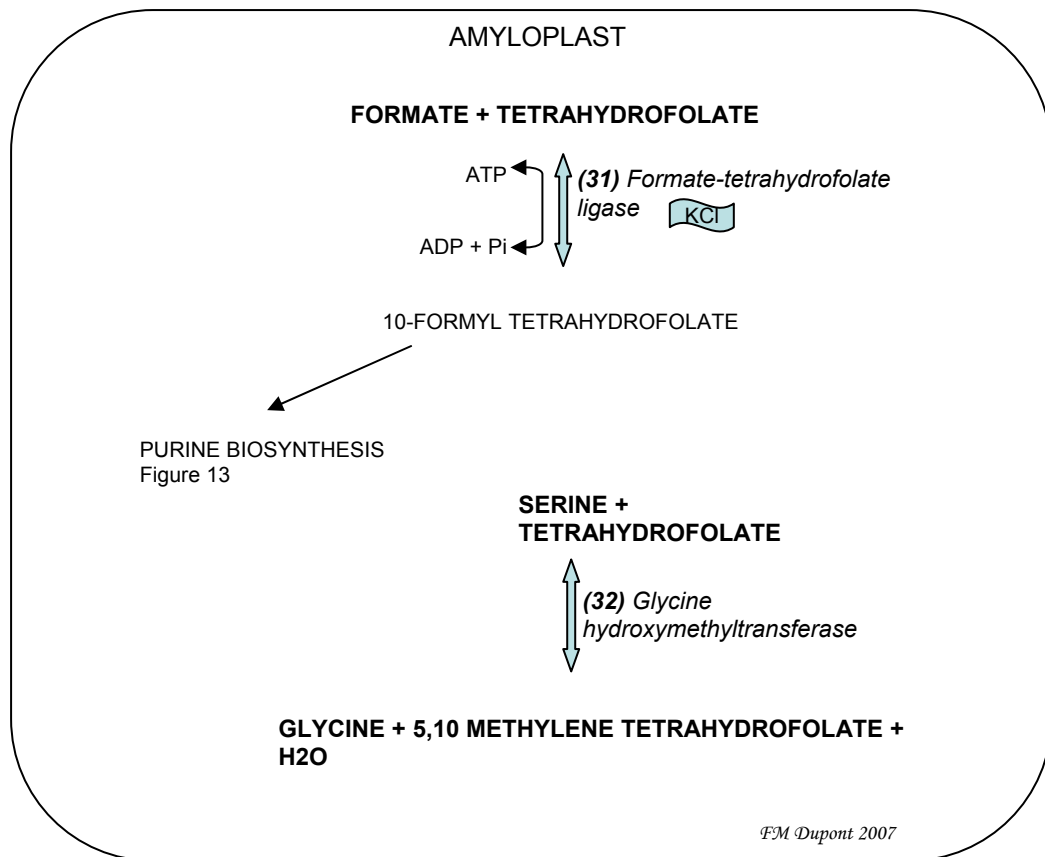

**Figure 6. Folate 1-carbon metabolism.**

Supplement: Additional file 2 — Figures 2-17. [file 1471-2229-8-39-S2.zip › fig 1 with links forfinal revised version/Fig6.pdf]

# AMYLOPLAST

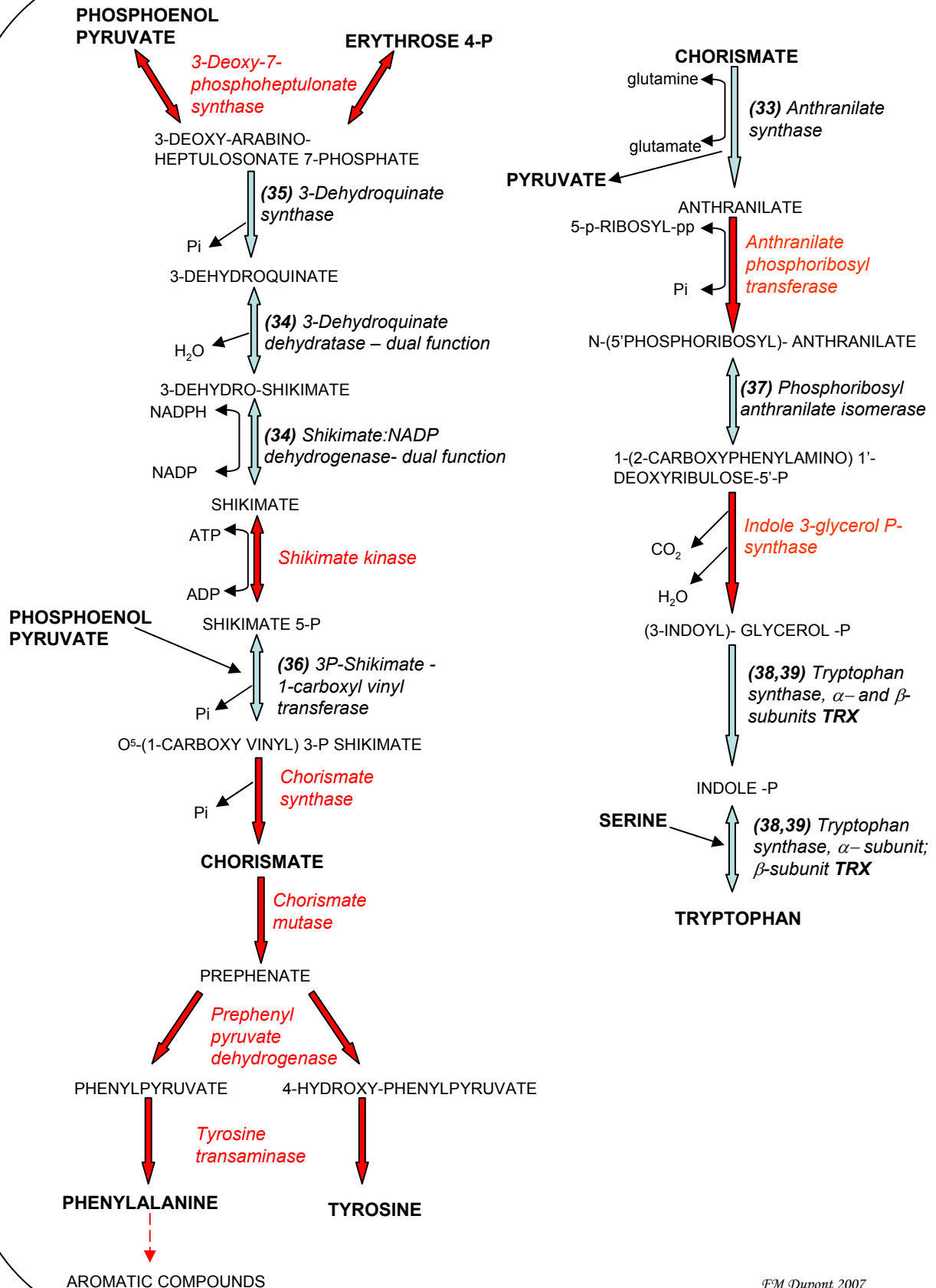

FM Dupont 2007

Figure 7. Aromatic amino acid synthesis.

Supplement: Additional file 2 — Figures 2-17. [file 1471-2229-8-39-S2.zip › fig 1 with links forfinal revised version/Fig7.pdf]

# AMYLOPLAST

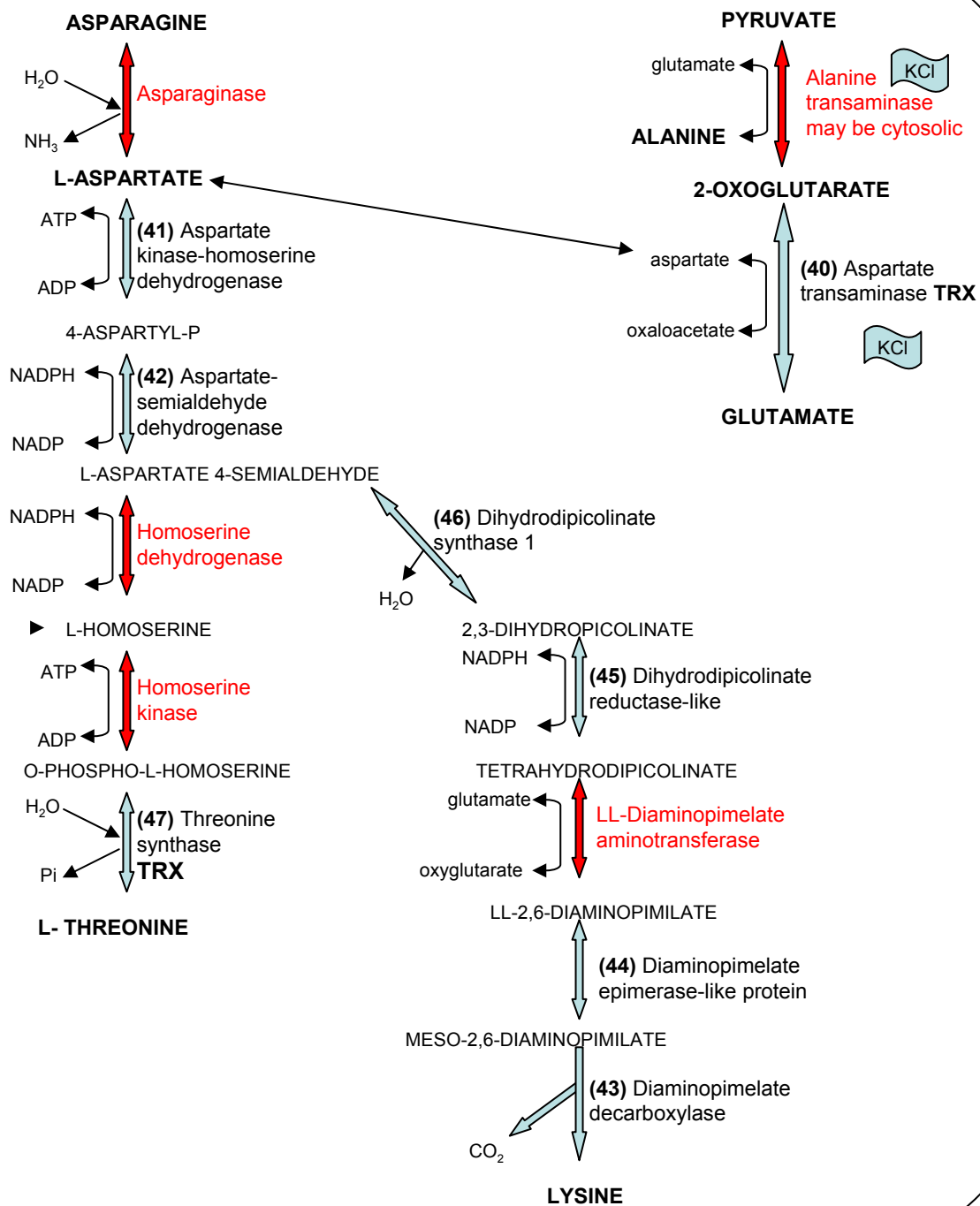

FM Dupont 2007

Figure 8. Aspartate family of amino acids.

Supplement: Additional file 2 — Figures 2-17. [file 1471-2229-8-39-S2.zip › fig 1 with links forfinal revised version/Fig8.pdf]

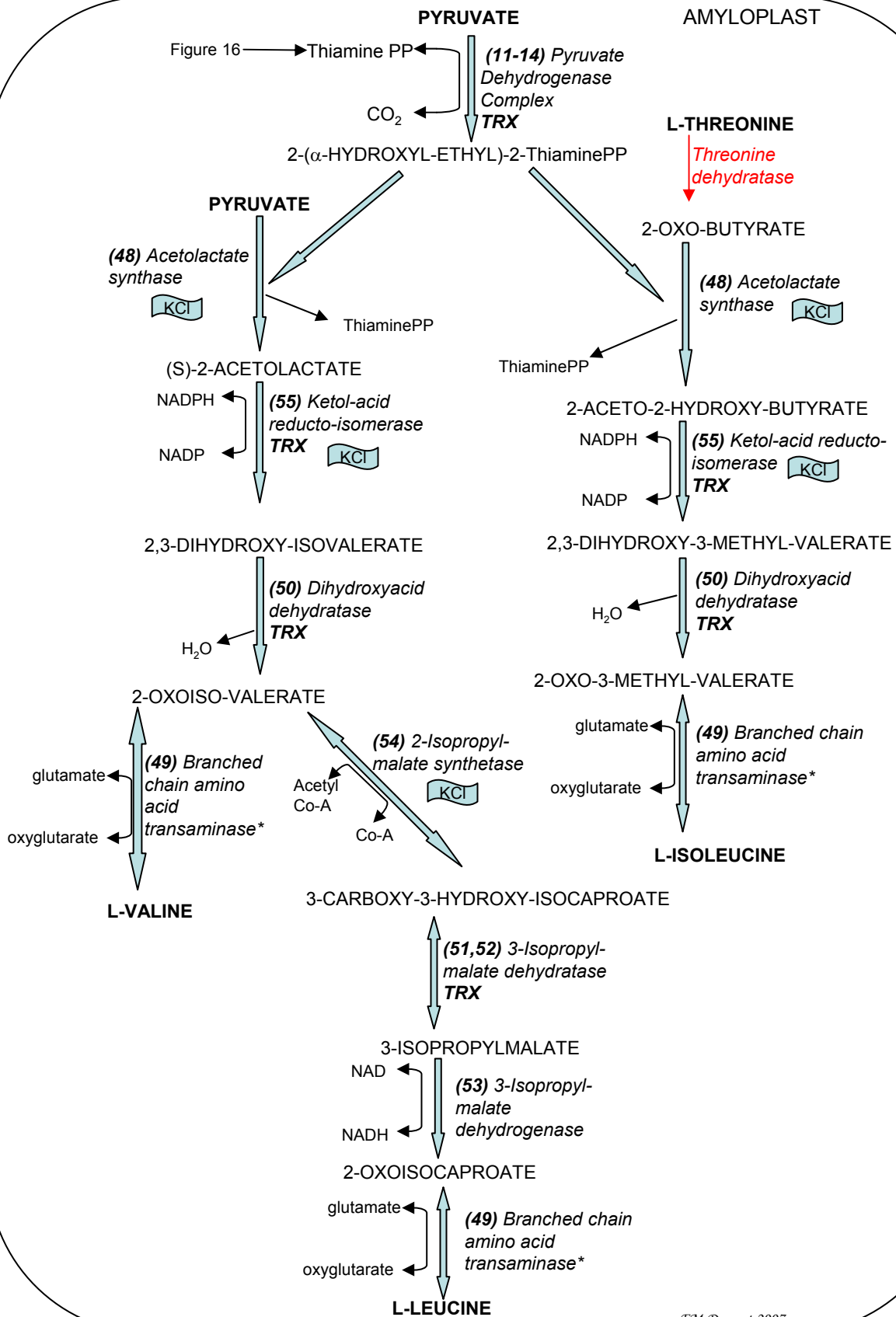

**Figure 9. Branched chain family of amino acids.**

Supplement: Additional file 2 — Figures 2-17. [file 1471-2229-8-39-S2.zip › fig 1 with links forfinal revised version/Fig9.pdf]

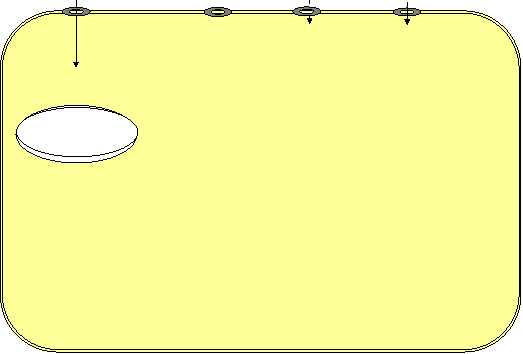

Supplement: Additional file 2 — Figures 2-17. [file 1471-2229-8-39-S2.zip › fig 1 with links forfinal revised version/Figure1/img002.gif]

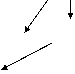

Supplement: Additional file 2 — Figures 2-17. [file 1471-2229-8-39-S2.zip › fig 1 with links forfinal revised version/Figure1/img003.gif]

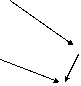

Supplement: Additional file 2 — Figures 2-17. [file 1471-2229-8-39-S2.zip › fig 1 with links forfinal revised version/Figure1/img004.gif]

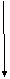

Supplement: Additional file 2 — Figures 2-17. [file 1471-2229-8-39-S2.zip › fig 1 with links forfinal revised version/Figure1/img005.gif]

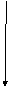

Supplement: Additional file 2 — Figures 2-17. [file 1471-2229-8-39-S2.zip › fig 1 with links forfinal revised version/Figure1/img006.gif]

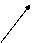

Supplement: Additional file 2 — Figures 2-17. [file 1471-2229-8-39-S2.zip › fig 1 with links forfinal revised version/Figure1/img007.gif]

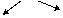

Supplement: Additional file 2 — Figures 2-17. [file 1471-2229-8-39-S2.zip › fig 1 with links forfinal revised version/Figure1/img008.gif]

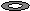

Supplement: Additional file 2 — Figures 2-17. [file 1471-2229-8-39-S2.zip › fig 1 with links forfinal revised version/Figure1/img009.gif]

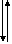

Supplement: Additional file 2 — Figures 2-17. [file 1471-2229-8-39-S2.zip › fig 1 with links forfinal revised version/Figure1/img010.gif]

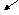

Supplement: Additional file 2 — Figures 2-17. [file 1471-2229-8-39-S2.zip › fig 1 with links forfinal revised version/Figure1/img011.gif]

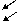

Supplement: Additional file 2 — Figures 2-17. [file 1471-2229-8-39-S2.zip › fig 1 with links forfinal revised version/Figure1/img012.gif]

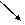

Supplement: Additional file 2 — Figures 2-17. [file 1471-2229-8-39-S2.zip › fig 1 with links forfinal revised version/Figure1/img013.gif]

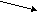

Supplement: Additional file 2 — Figures 2-17. [file 1471-2229-8-39-S2.zip › fig 1 with links forfinal revised version/Figure1/img014.gif]

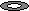

Supplement: Additional file 2 — Figures 2-17. [file 1471-2229-8-39-S2.zip › fig 1 with links forfinal revised version/Figure1/img015.gif]

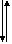

Supplement: Additional file 2 — Figures 2-17. [file 1471-2229-8-39-S2.zip › fig 1 with links forfinal revised version/Figure1/img016.gif]

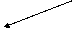

Supplement: Additional file 2 — Figures 2-17. [file 1471-2229-8-39-S2.zip › fig 1 with links forfinal revised version/Figure1/img017.gif]

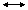

Supplement: Additional file 2 — Figures 2-17. [file 1471-2229-8-39-S2.zip › fig 1 with links forfinal revised version/Figure1/img018.gif]

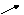

Supplement: Additional file 2 — Figures 2-17. [file 1471-2229-8-39-S2.zip › fig 1 with links forfinal revised version/Figure1/img019.gif]

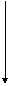

Supplement: Additional file 2 — Figures 2-17. [file 1471-2229-8-39-S2.zip › fig 1 with links forfinal revised version/Figure1/img020.gif]

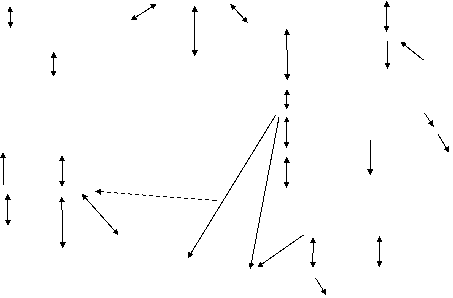

Supplement: Additional file 2 — Figures 2-17. [file 1471-2229-8-39-S2.zip › fig 1 with links forfinal revised version/Figure1/img021.gif]

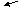

Supplement: Additional file 2 — Figures 2-17. [file 1471-2229-8-39-S2.zip › fig 1 with links forfinal revised version/Figure1/img022.gif]

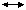

Supplement: Additional file 2 — Figures 2-17. [file 1471-2229-8-39-S2.zip › fig 1 with links forfinal revised version/Figure1/img023.gif]

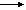

Supplement: Additional file 2 — Figures 2-17. [file 1471-2229-8-39-S2.zip › fig 1 with links forfinal revised version/Figure1/img024.gif]

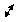

Supplement: Additional file 2 — Figures 2-17. [file 1471-2229-8-39-S2.zip › fig 1 with links forfinal revised version/Figure1/img025.gif]

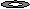

Supplement: Additional file 2 — Figures 2-17. [file 1471-2229-8-39-S2.zip › fig 1 with links forfinal revised version/Figure1/img026.gif]

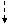

Supplement: Additional file 2 — Figures 2-17. [file 1471-2229-8-39-S2.zip › fig 1 with links forfinal revised version/Figure1/img027.gif]

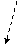

Supplement: Additional file 2 — Figures 2-17. [file 1471-2229-8-39-S2.zip › fig 1 with links forfinal revised version/Figure1/img028.gif]

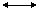

Supplement: Additional file 2 — Figures 2-17. [file 1471-2229-8-39-S2.zip › fig 1 with links forfinal revised version/Figure1/img029.gif]

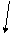

Supplement: Additional file 2 — Figures 2-17. [file 1471-2229-8-39-S2.zip › fig 1 with links forfinal revised version/Figure1/img030.gif]

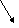

Supplement: Additional file 2 — Figures 2-17. [file 1471-2229-8-39-S2.zip › fig 1 with links forfinal revised version/Figure1/img031.gif]

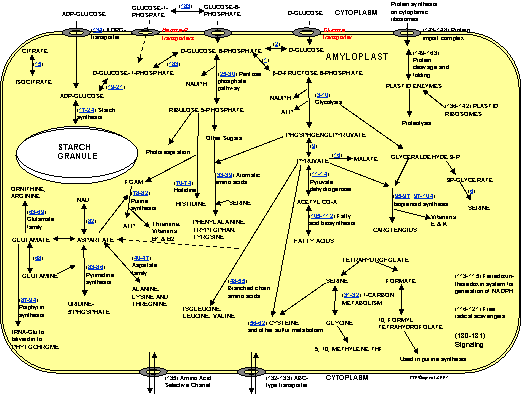

Supplement: Additional file 2 — Figures 2-17. [file 1471-2229-8-39-S2.zip › fig 1 with links forfinal revised version/Figure1/v3img001.gif]

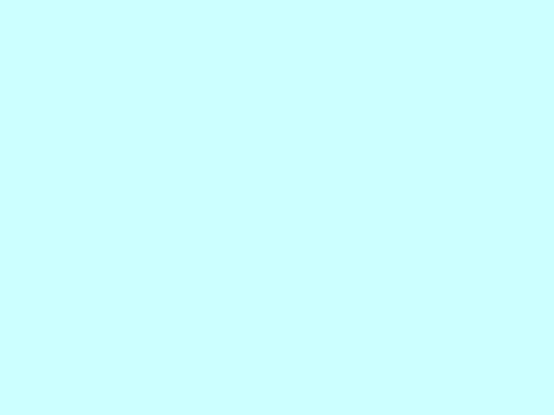

Supplement: Additional file 2 — Figures 2-17. [file 1471-2229-8-39-S2.zip › fig 1 with links forfinal revised version/Figure1/v3s0001i.gif]

Supplement: Additional file 2 — Figures 2-17. [file 1471-2229-8-39-S2.zip › fig 1 with links forfinal revised version/Figure1/v3space.gif]
