# Supplementary material for: Metabolic pathways of the wheat (Triticum aestivum) endosperm amyloplast revealed by proteomics
Source: BMC Plant Biol. 2008 Apr 17;8:39. doi: 10.1186/1471-2229-8-39 (PMC2383896; doi:10.1186/1471-2229-8-39)
Supplement: Additional file 3 — Figure 2-17. Detailed hypothetical pathways mapping enzymes of biosynthetic pathways in the wheat endosperm amyloplast. [file 1471-2229-8-39-S3.pdf]

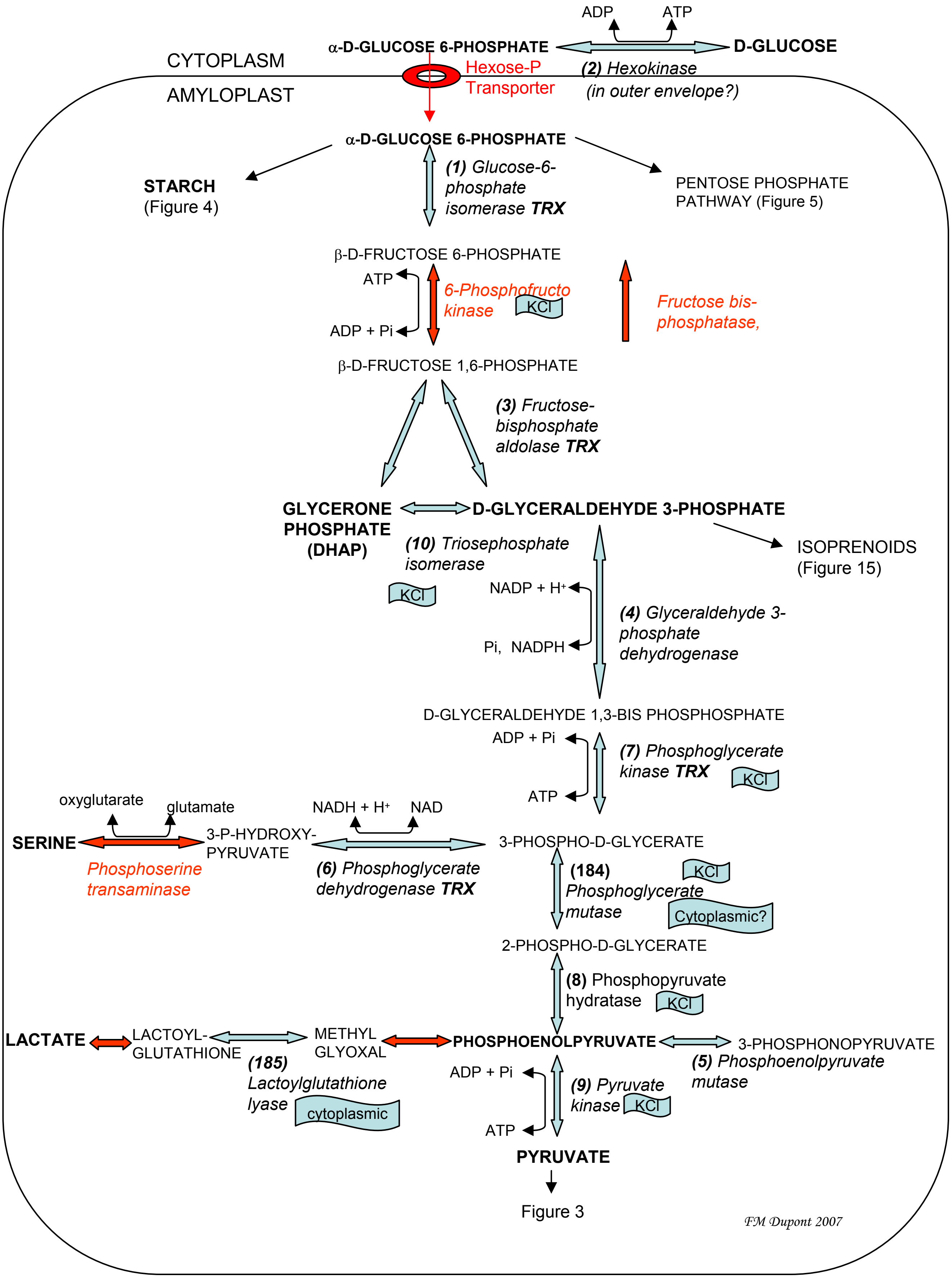

Figure 2. Glucose metabolism and glycolysis. Details of symbols and meaning of colors for all figures are given in Fig. 3. Hexokinase may be in the outer envelope, or within the plastid.

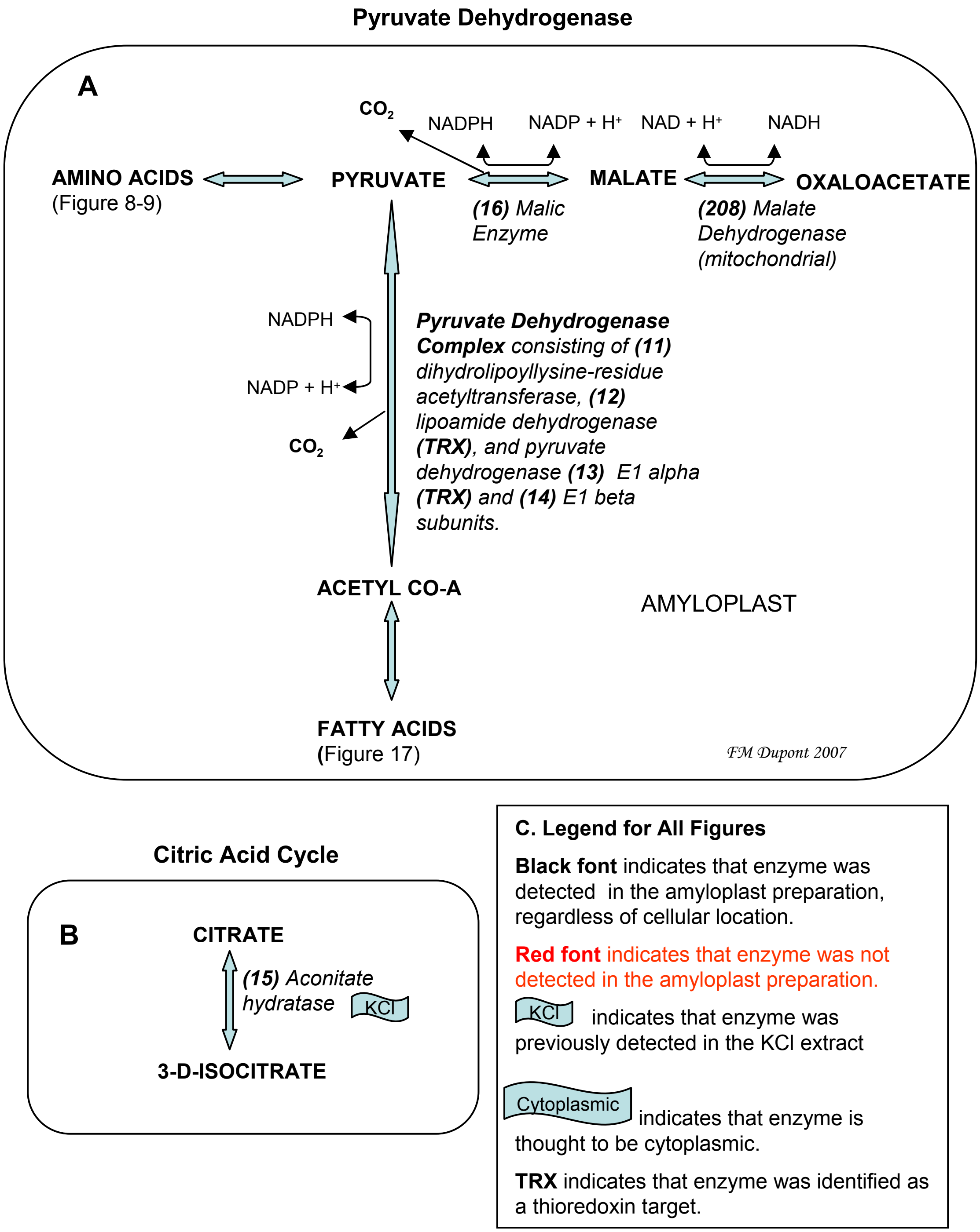

Figure 3 . A. Pyruvate dehydrogenase. B. Citric acid cycle components C. Legend for all figures.

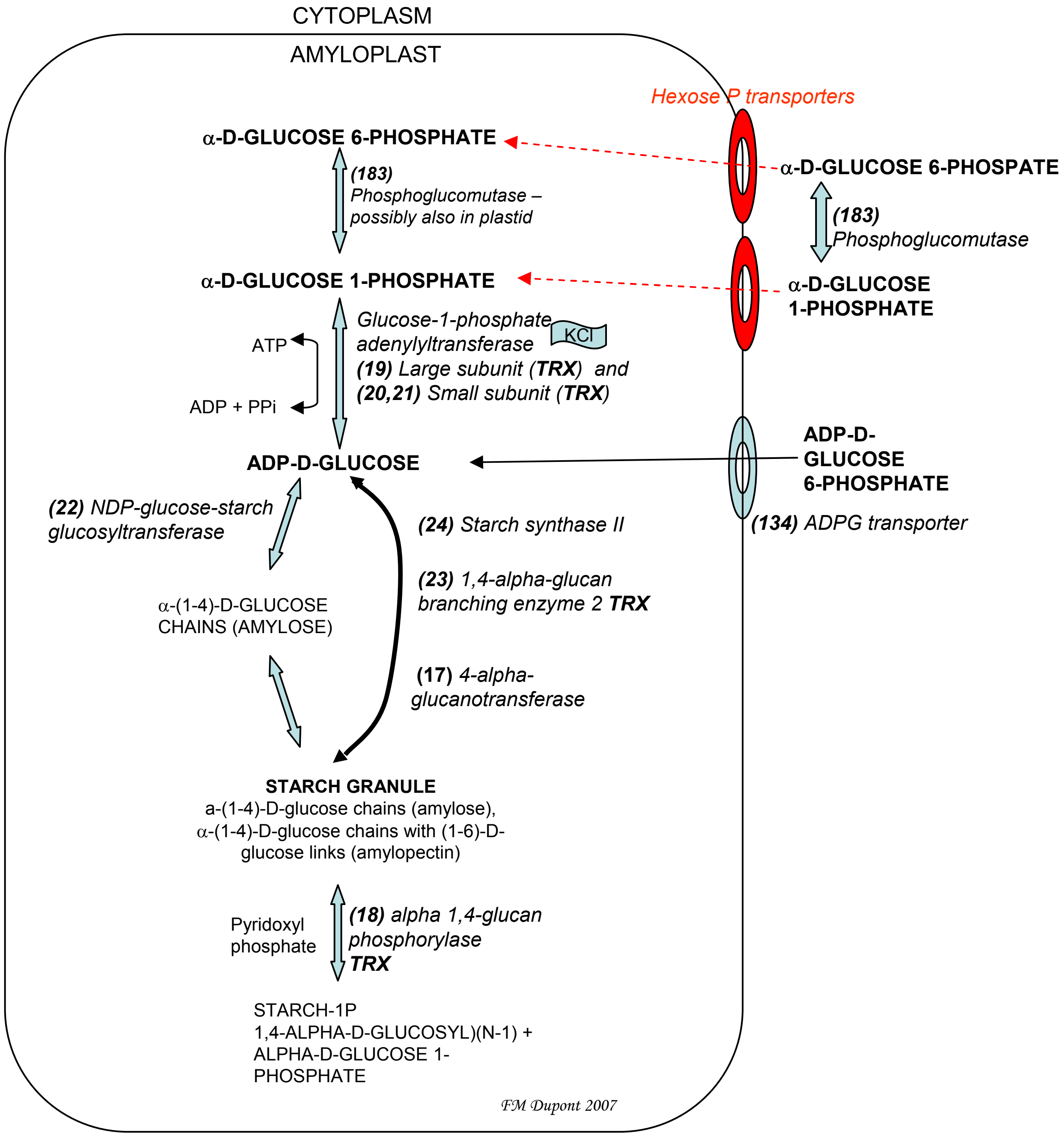

Figure 4. Starch biosynthesis.

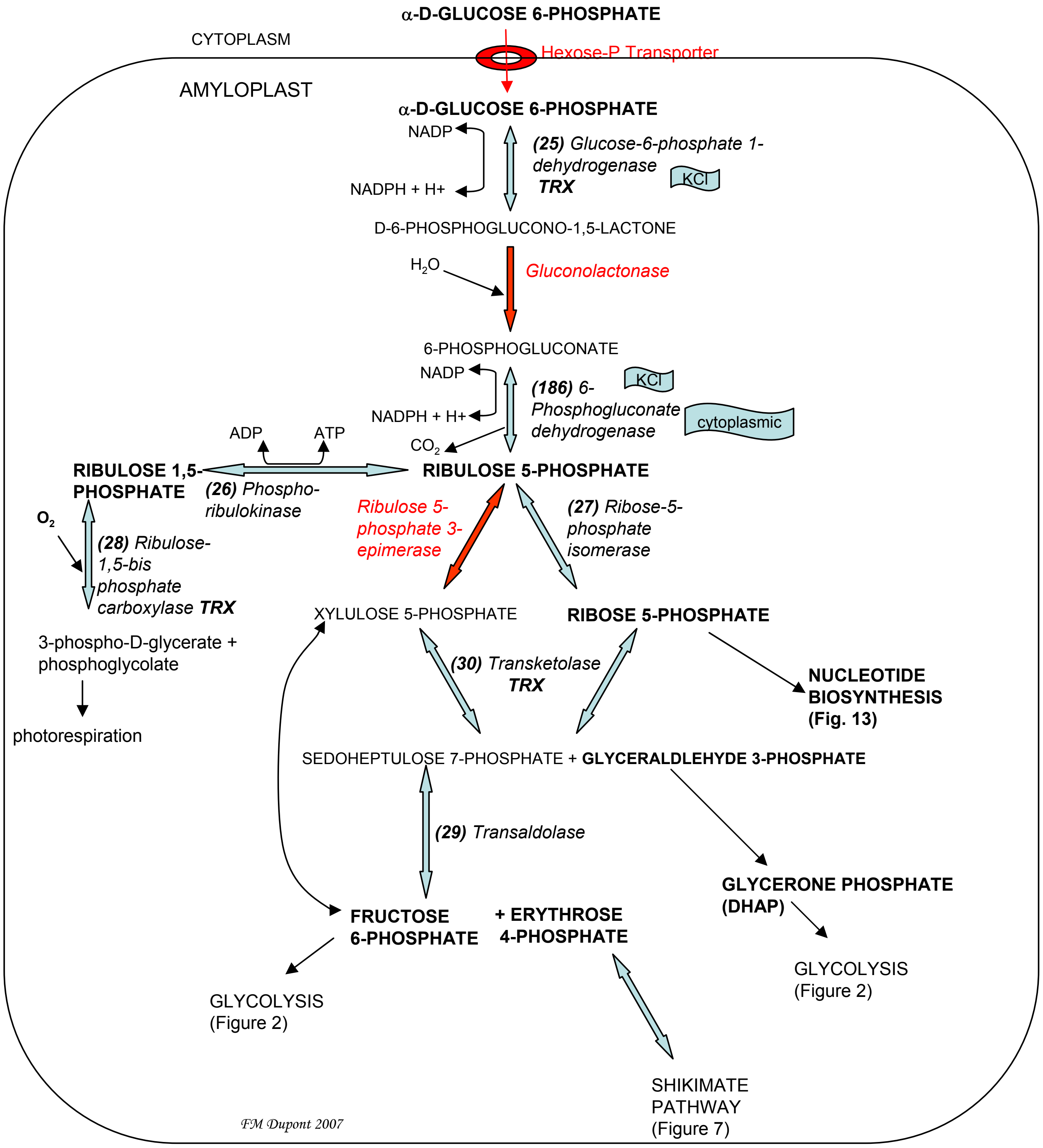

Figure 5. Pentose phosphate pathway.

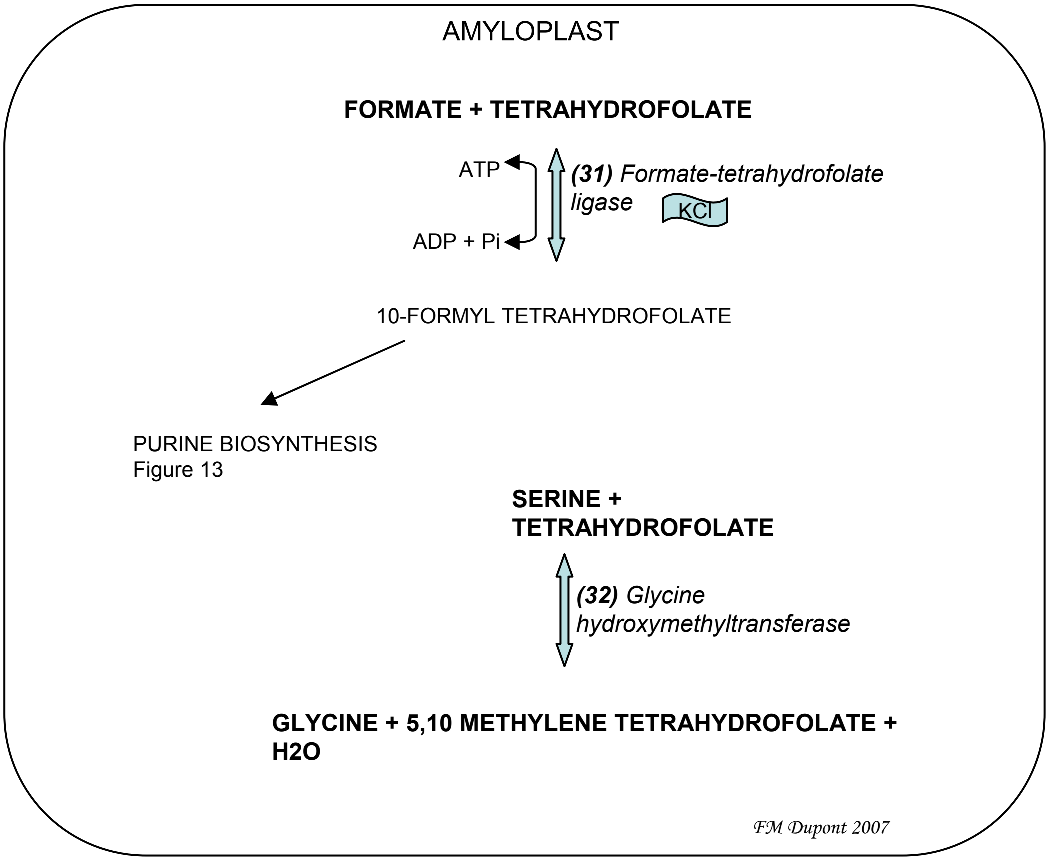

Figure 6. Folate 1-carbon metabolism.

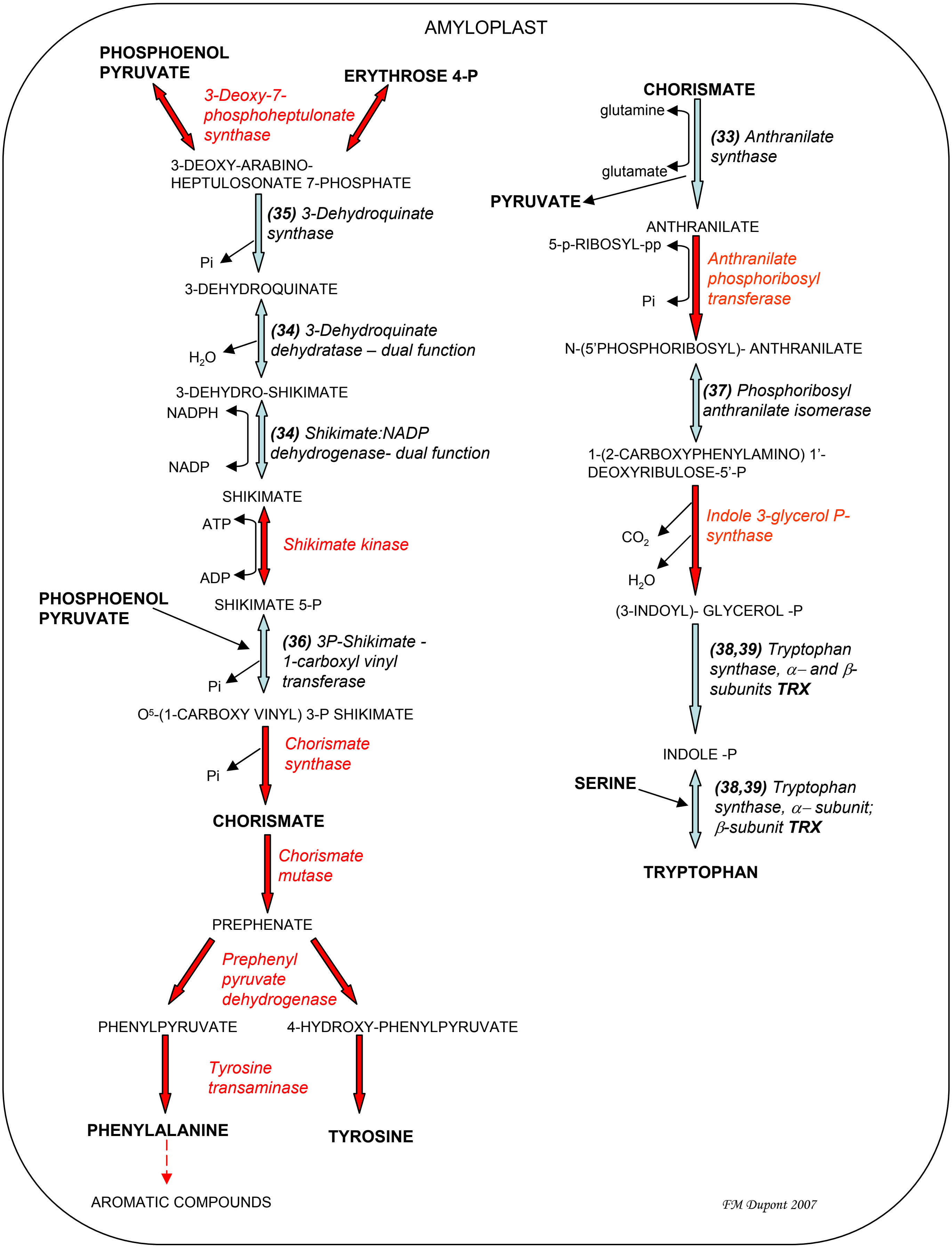

Figure 7. Aromatic amino acid synthesis.

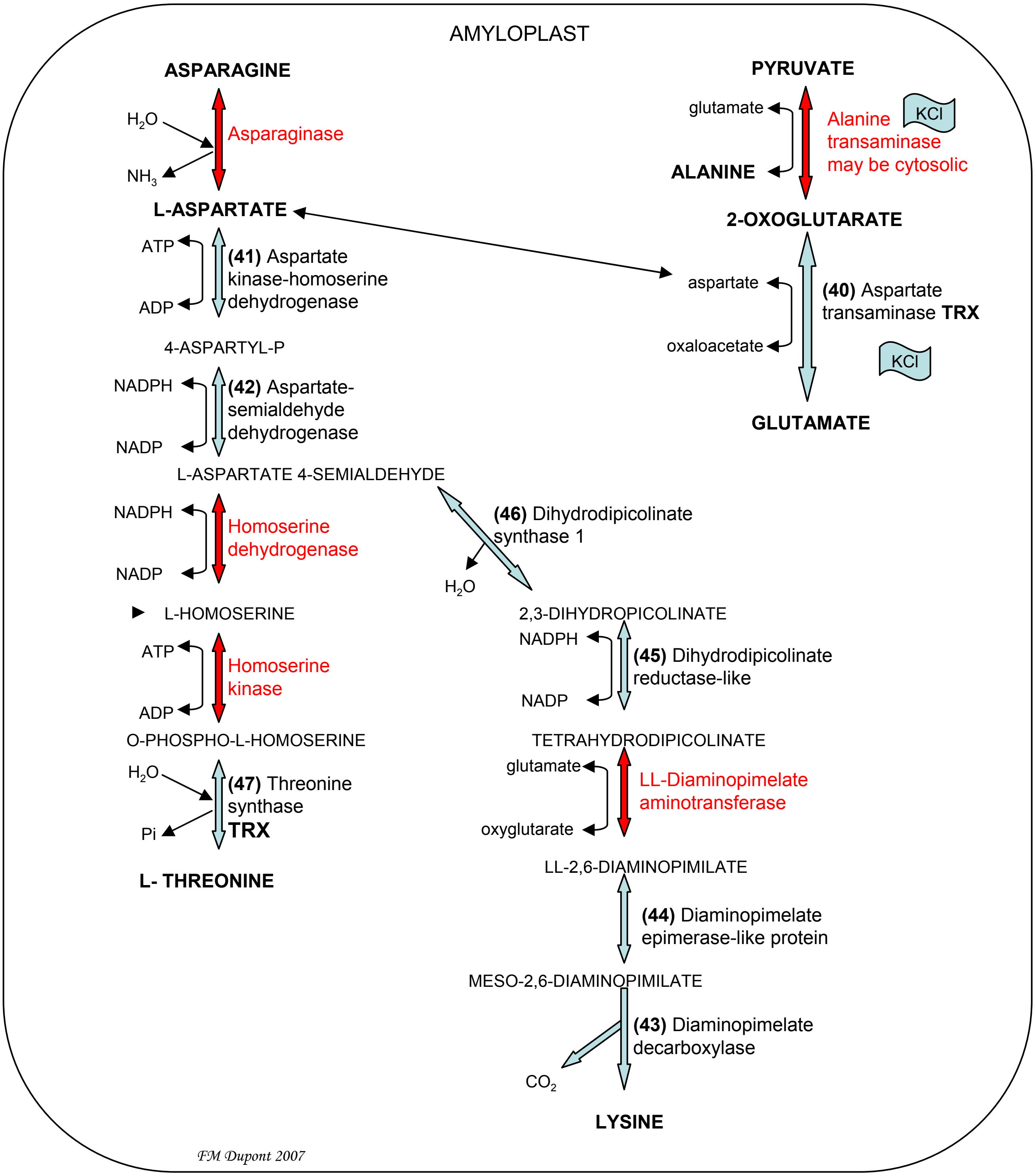

Figure 8. Aspartate family of amino acids.

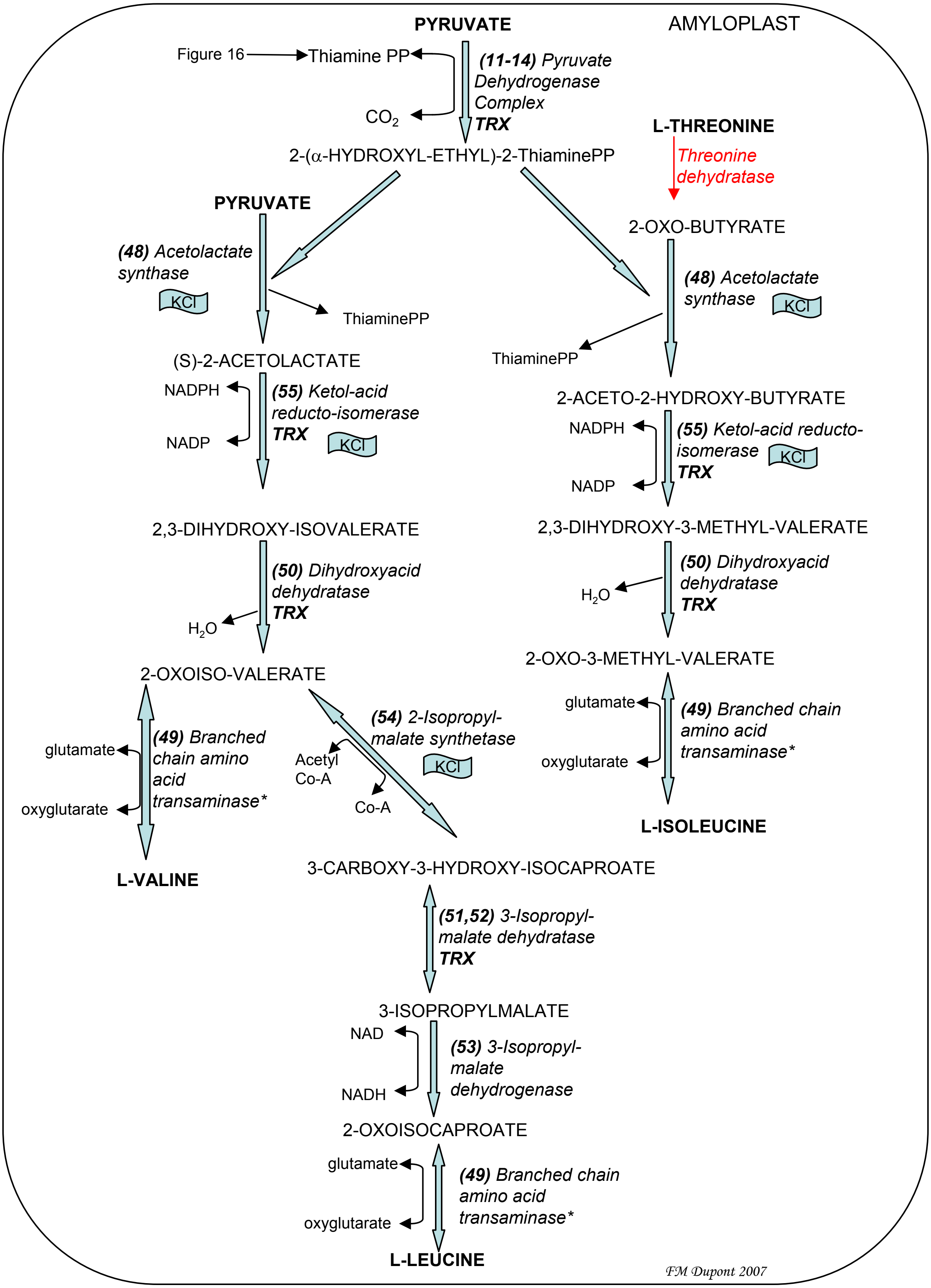

Figure 9. Branched chain family of amino acids.

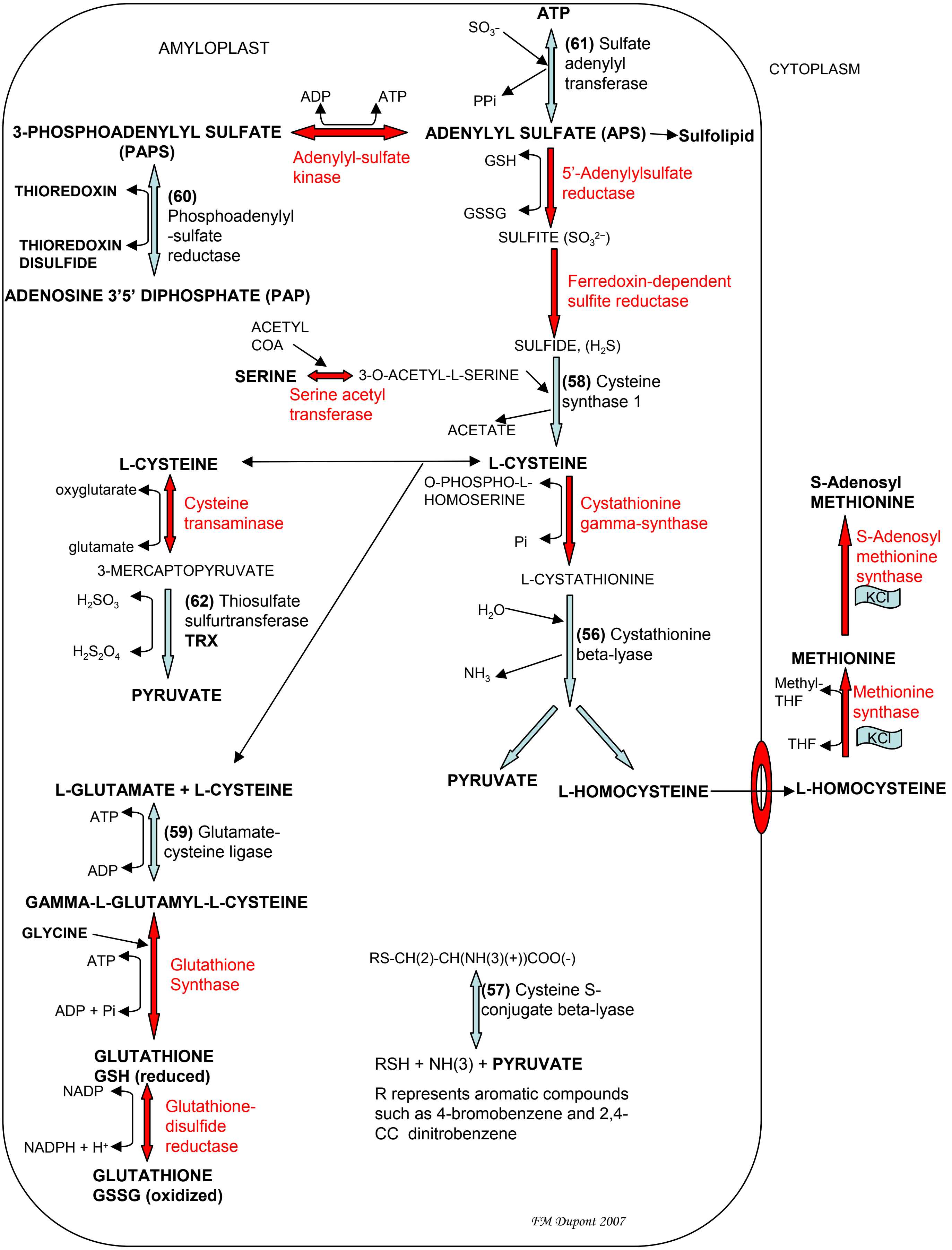

Figure 10. Cysteine and sulfur metabolism.

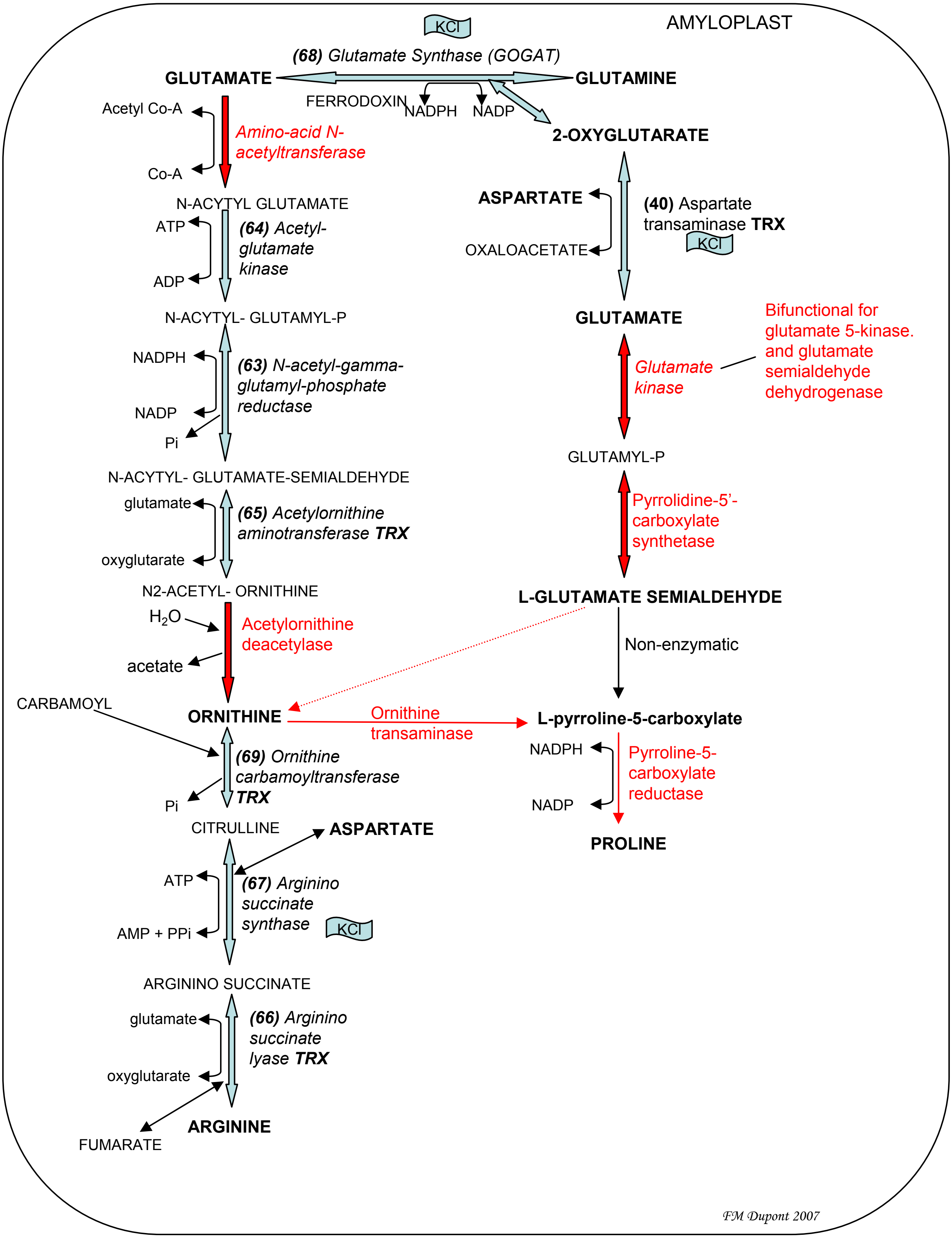

Figure 11. Glutamate family of amino acids.

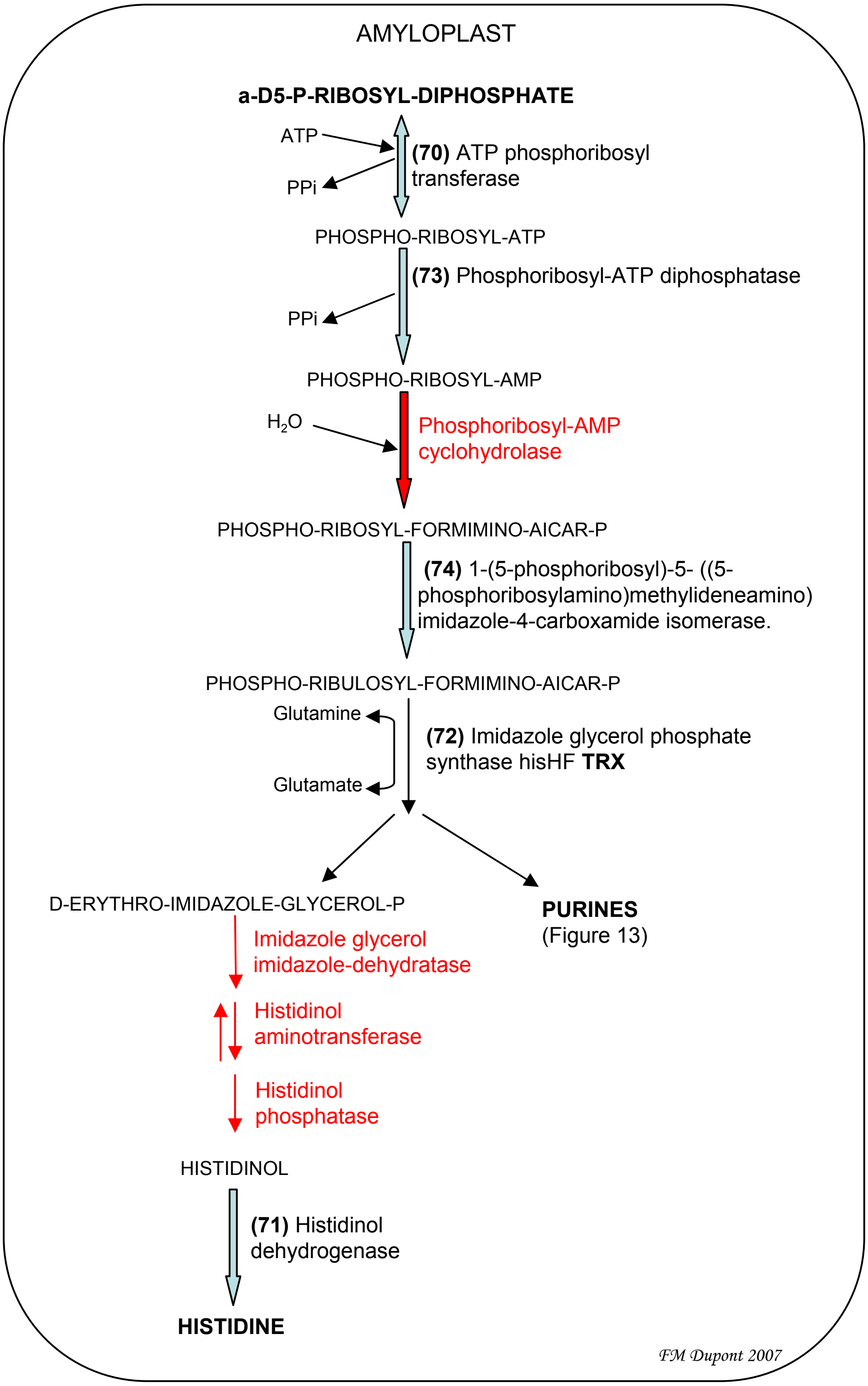

Figure 12. Histidine synthesis.

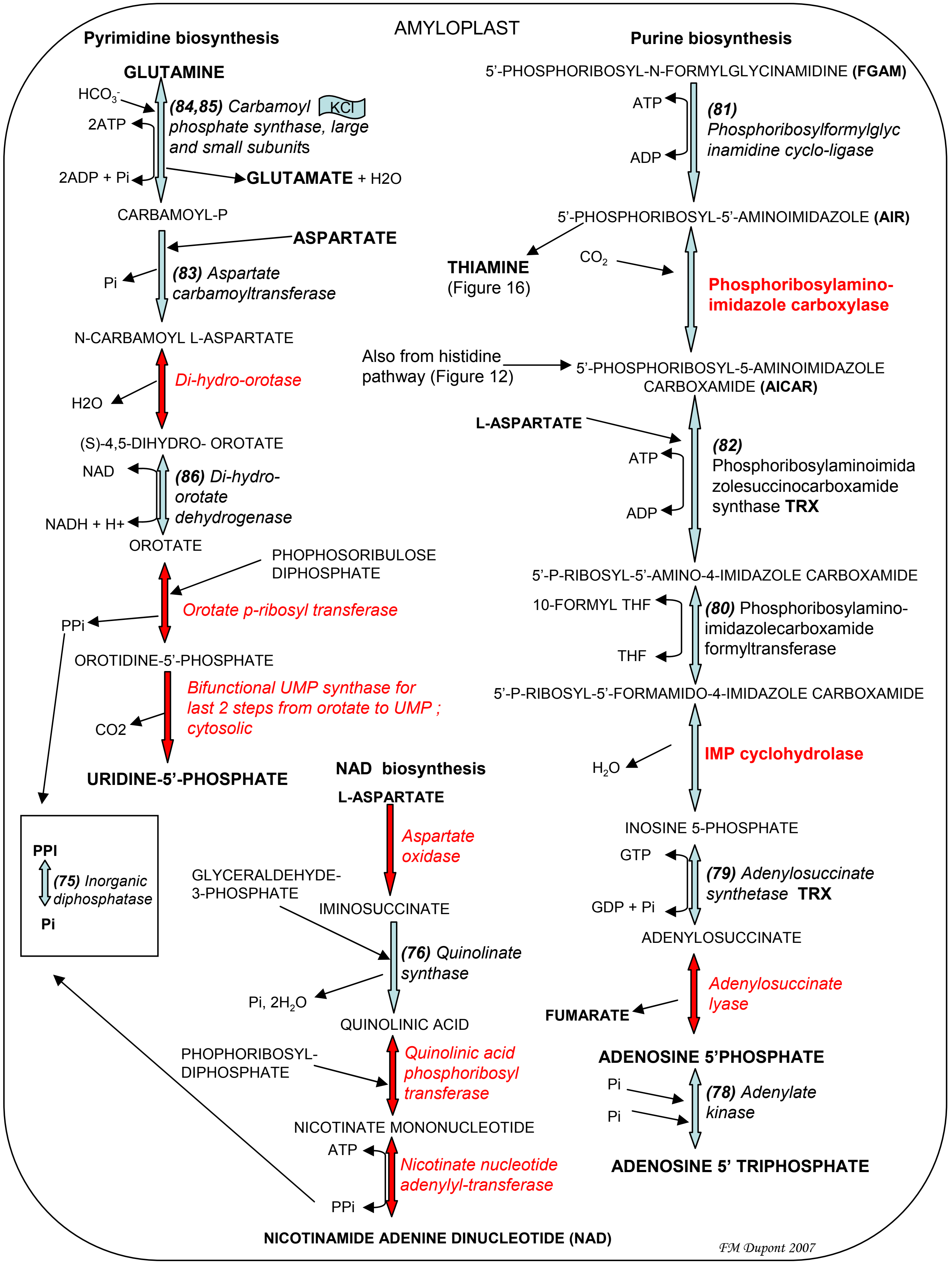

Figure 13. Nucleic acid synthesis.

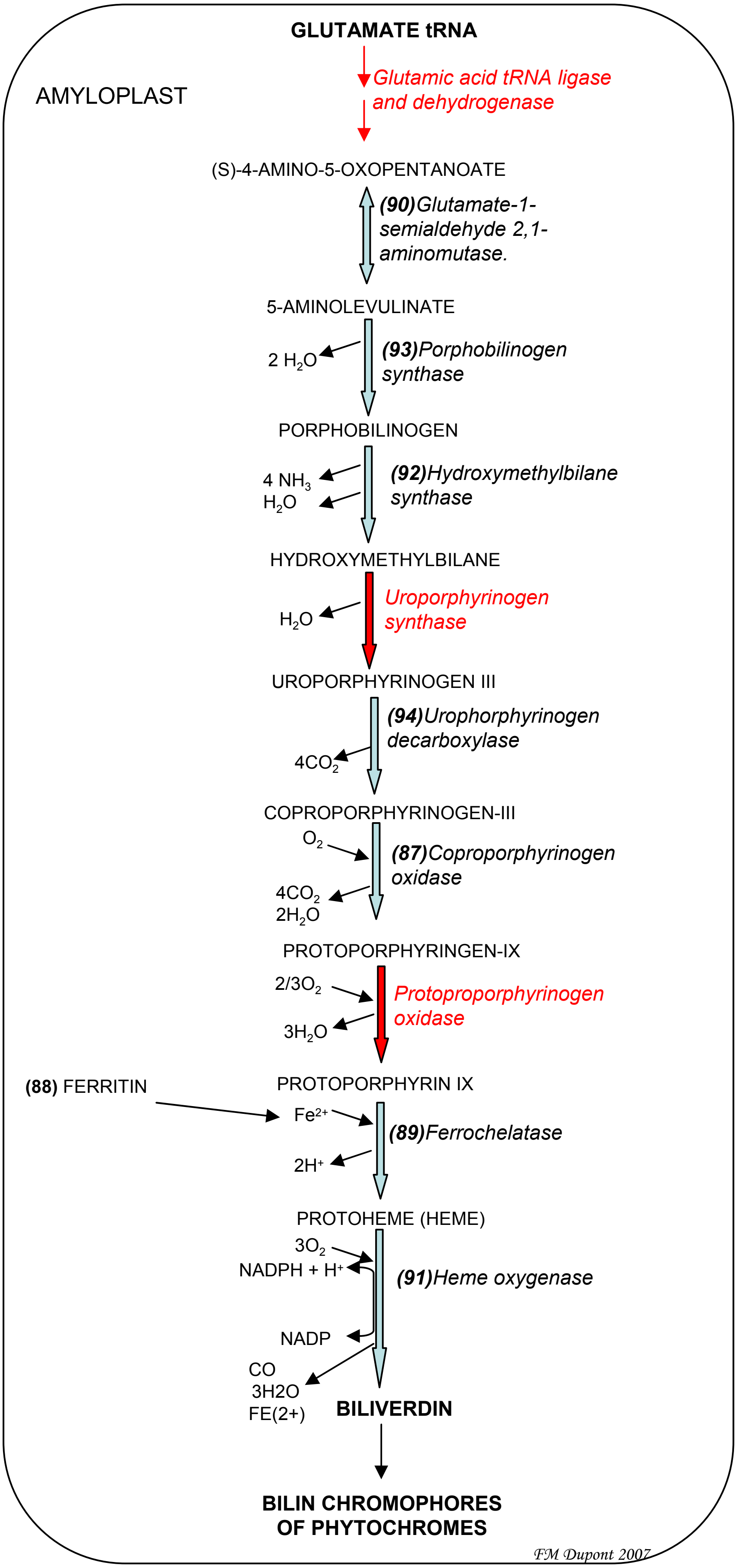

Figure 14. Porphyrin synthesis.

Isoprenoid Biosynthesis Part I: Non-mevalonate Pathway

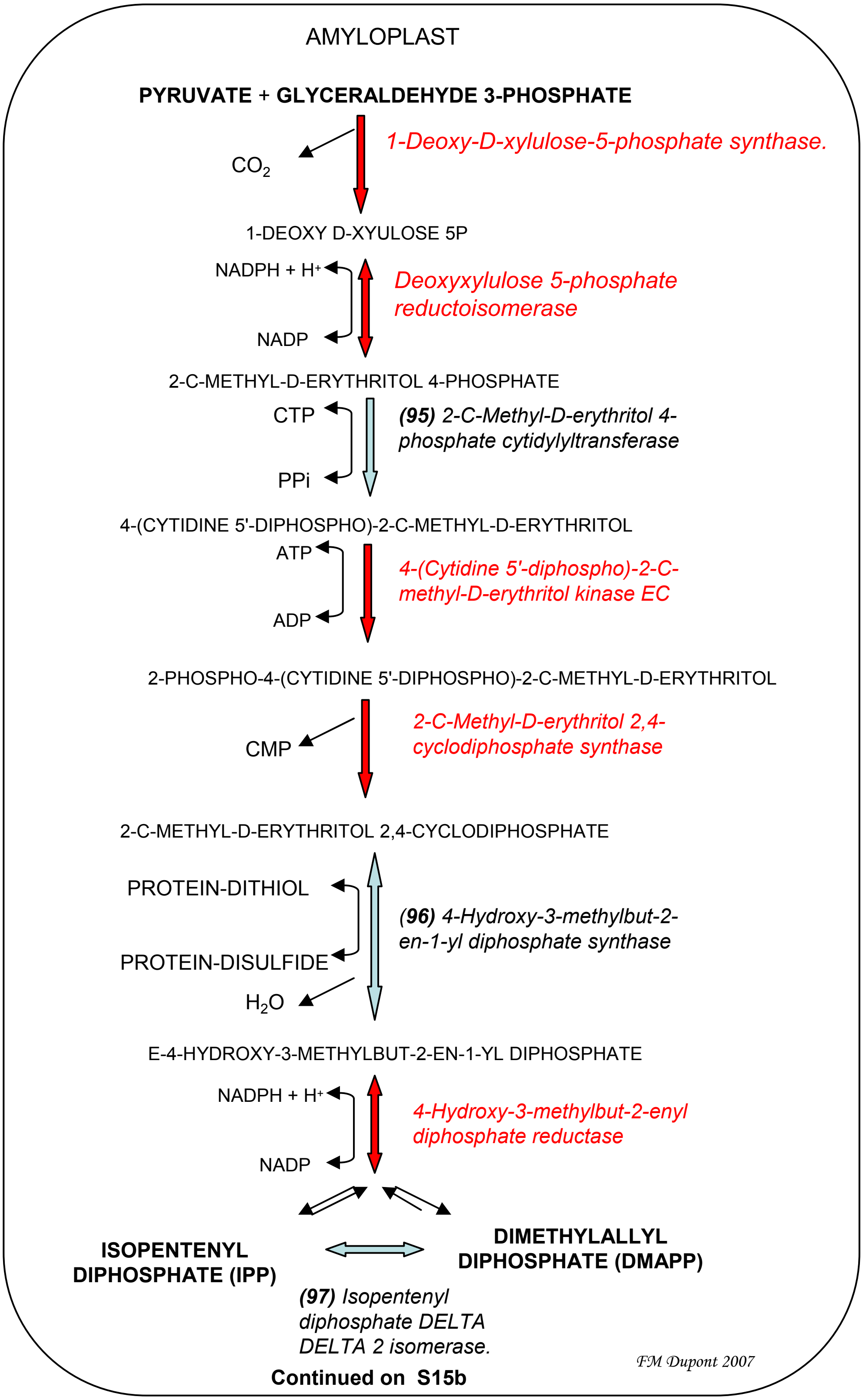

Figure 15a. Isoprenoid synthesis I, Non-mevalonate pathway.

Isoprenoid Biosynthesis II

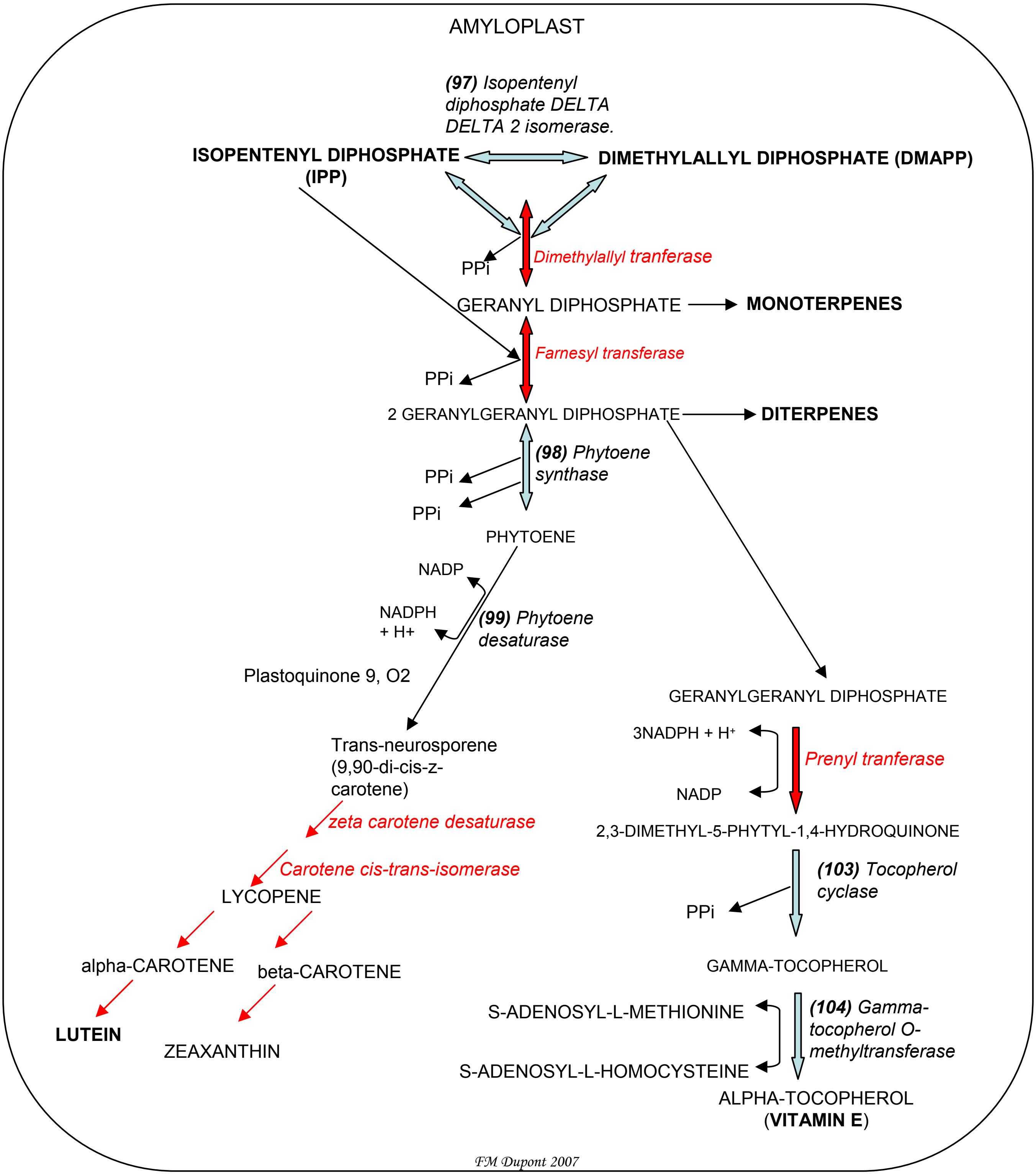

Figure 15b. Isoprenoid synthesis II.

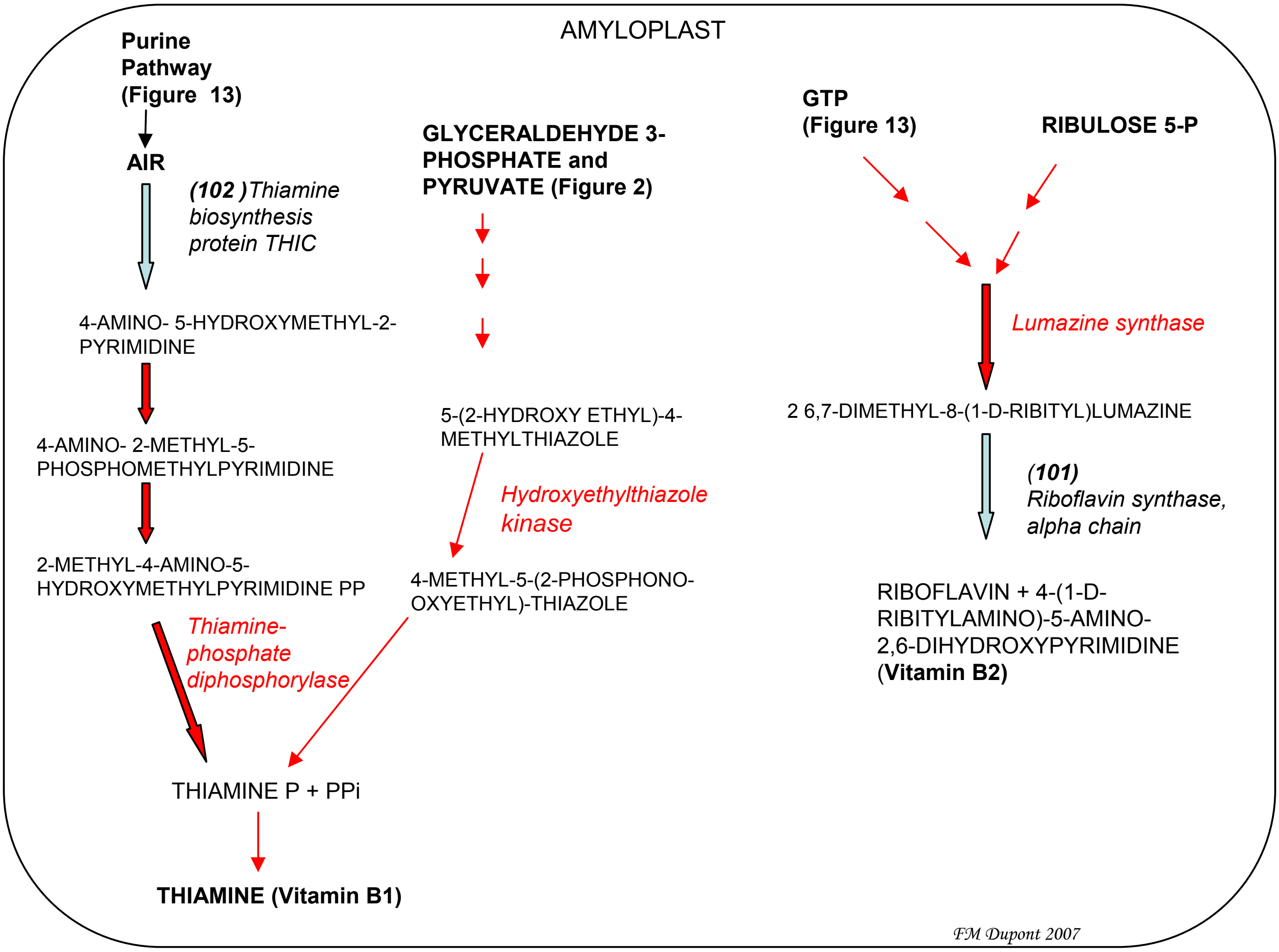

Figure 16. Vitamin and cofactor synthesis, nucleic acid-related pathways.

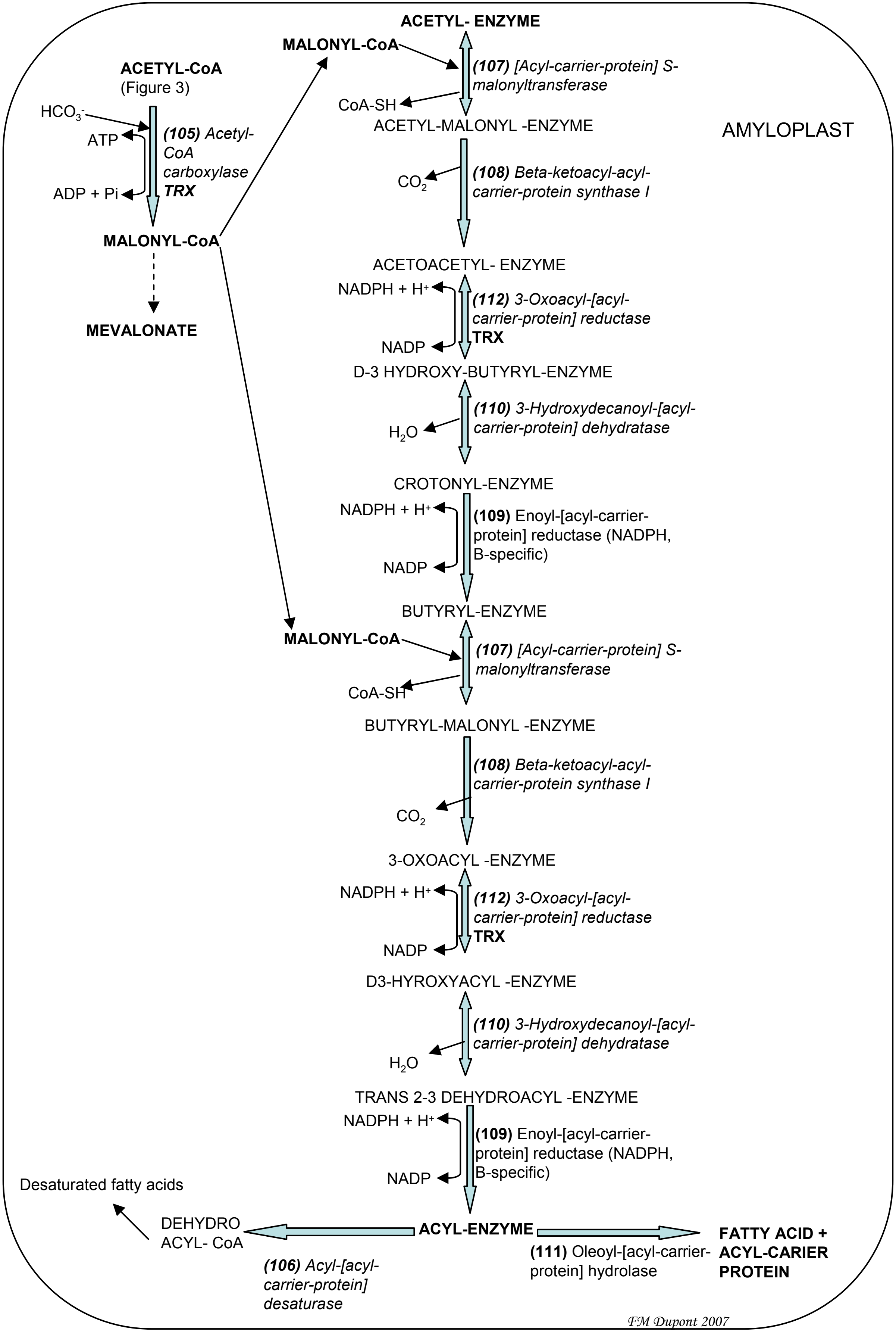

Figure 17. Fatty Acid Synthesis. Only 2 cycles from addition of malonyl-CoA to formation of the acyl-enzyme are shown. Multiple cycles are needed to form 16 and 18 carbon fatty acids.
